# Mass spectrometric assays monitoring the deubiquitinase activity of the SARS-CoV-2 papain-like protease inform on the basis of substrate selectivity and have utility for substrate identification

**DOI:** 10.1016/j.bmc.2023.117498

**Published:** 2023-11-15

**Authors:** Lennart Brewitz, H.T. Henry Chan, Petra Lukacik, Claire Strain-Damerell, Martin A. Walsh, Fernanda Duarte, Christopher J. Schofield

**Affiliations:** a Chemistry Research Laboratory, Department of Chemistry, University of Oxford, 12 Mansfield Road, OX1 3TA Oxford, United Kingdom; b The Ineos Oxford Institute for Antimicrobial Research, Department of Chemistry, University of Oxford, 12 Mansfield Road, OX1 3TA Oxford, United Kingdom; c Diamond Light Source Ltd., Harwell Science and Innovation Campus, OX11 0DE Didcot, United Kingdom; d Research Complex at Harwell, Harwell Science and Innovation Campus, OX11 0FA Didcot, United Kingdom

**Keywords:** SARS-CoV-2 papain-like protease / PL^pro^, Nucleophilic cysteine protease, Deubiquitinase / DUB, Ubiquitin, Interferon stimulated gene 15 / ISG15, Mass spectrometry, Ubiquitin-like modifier, Virus-host interactions, Interferon regulatory factor 3 / IRF3, eIF4E-homologous protein / 4EHP

## Abstract

The SARS-CoV-2 papain-like protease (PL^pro^) and main protease (M^pro^) are nucleophilic cysteine enzymes that catalyze hydrolysis of the viral polyproteins pp1a/1ab. By contrast with M^pro^, PL^pro^ is also a deubiquitinase (DUB) that accepts post-translationally modified human proteins as substrates. Here we report studies on the DUB activity of PL^pro^ using synthetic *N*^ε^-lysine-branched oligopeptides as substrates that mimic post-translational protein modifications by ubiquitin (Ub) or Ub-like modifiers (UBLs), such as interferon stimulated gene 15 (ISG15). Mass spectrometry (MS)-based assays confirm the DUB activity of isolated recombinant PL^pro^. They reveal that the sequence of both the peptide fragment derived from the post-translationally modified protein and that derived from the UBL affects PL^pro^ catalysis; the nature of substrate binding in the S sites appears to be more important for catalytic efficiency than binding in the S′ sites. Importantly, the results reflect the reported cellular substrate selectivity of PL^pro^, *i.e.* human proteins conjugated to ISG15 are better substrates than those conjugated to Ub or other UBLs. The combined experimental and modelling results imply that PL^pro^ catalysis is affected not only by the identity of the substrate residues binding in the S and S′ sites, but also by the substrate fold and the conformational dynamics of the blocking loop 2 of the PL^pro^:substrate complex. *N*^ε^-Lysine-branched oligopeptides thus have potential to help the identification of PL^pro^ substrates. More generally, the results imply that MS-based assays with *N*^ε^-lysine-branched oligopeptides have potential to monitor catalysis by human DUBs and hence to inform on their substrate preferences.

## Graphical abstract

**Figure:**
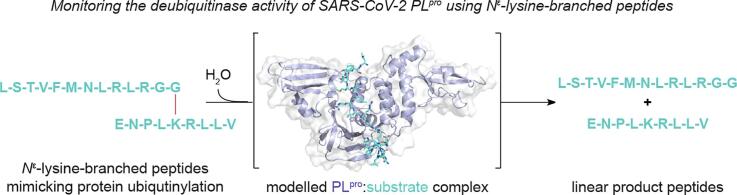

## 1 Introduction

The SARS-CoV-2 genome encodes two nucleophilic cysteine proteases that catalyze hydrolysis of the viral polyproteins pp1a/1ab into functional non-structural proteins (nsps), *i.e.* the papain-like protease (PL^pro^, a domain of nsp3) and the main protease (M^pro^, nsp5).^1^, ^2^ PL^pro^ catalyzes the hydrolysis of peptide bonds in pp1a/1ab C-terminal to three LXGG motifs (X represents a non-conserved residue) to release nsps1-3. M^pro^ catalyzes the hydrolysis of peptide bonds in pp1a/1ab C-terminal to eleven glutamine residues in (L/F/V)Q(S/A/N) motifs to release nsps 4–16.^1^ Catalysis by the two SARS-CoV-2 proteases is essential for viral replication and their inhibition is of therapeutic relevance,^3^, ^4^, ^5^, ^6^, ^7^, ^8^ *i.e.* the small-molecules nirmatrelvir and ensitrelvir, which are clinically used to treat COVID-19, inhibit M^pro^.^9^, ^10^

The proteolytic functions of both SARS-CoV-2 M^pro^ and, in particular, PL^pro^ extend beyond catalyzing the hydrolysis of pp1a/1ab to catalyzing the hydrolysis of (iso)peptide amide bonds in human proteins, in accord with the reported substrate promiscuity of other coronavirus proteases.^11^, ^12^, ^13^, ^14^, ^15^ Interestingly, only a few of the human proteins that contain (L/F/V)Q motifs in their coding sequence are validated substrates of isolated recombinant SARS-CoV-2 M^pro^, *e.g.* human nuclear factor (NF)-κB essential modulator (NEMO), the M^pro^-catalyzed hydrolysis of which has been reported to induce the death of brain endothelial cells.^16^, ^17^

PL^pro^ is proposed to catalyze the hydrolysis of peptide bonds C-terminal to LXGG motifs in the coding sequence of several human proteins, *e.g.* the serine/threonine-protein kinase ULK1 and protein S, on the basis of *in vitro* and/or cellular studies.^18^, ^19^, ^20^ Although proteomic studies with samples from COVID-19 patients indicate that PL^pro^ may accept human proteins as substrates *in vivo*,^21^ both the extent and the clinical significance of the PL^pro^-catalyzed degradation of human host proteins during SARS-CoV-2 infections are incompletely understood. Such knowledge is important with respect to understanding the consequences of therapeutic PL^pro^ inhibition.

LXGG motifs are conserved in human proteins which are post-translationally modified with ubiquitin (Ub) or ubiquitin-like modifiers (UBL), *e.g.* interferon stimulated gene 15 (ISG15),^22^, ^23^, ^24^, ^25^ neural precursor cell expressed, developmentally down-regulated 8 (NEDD8),^26^, ^27^ and ubiquitin-related modifier-1 (URM1).^28^, ^29^ Ub and UBLs are linked to one or multiple *N*^ε^-amino groups of a protein lysine residue via an isopeptide amide bond formed with their C-terminal residue, which forms a LXGG motif in the case of Ub, ISG15, NEDD8, and URM1.^30^, ^31^ The post-translational (poly)ubiquitinylation of human proteins has multiple regulatory functions, including signaling proteins for degradation via the proteasome.^32^, ^33^, ^34^ Protein (poly)ubiquitinylation is often reversible; formation of the isopeptide amide bond is catalyzed by Ub/UBL ligases while its hydrolysis is catalyzed by deubiquitinases (DUBs); both Ub/UBL ligases and DUBs may show high levels of specificity, both with respect to the substrate protein and/or the Ub/UBL.^33^, ^34^, ^35^, ^36^, ^37^, ^38^

SARS-CoV-2 PL^pro^ not only catalyzes hydrolysis of the pp1a/1ab polyproteins, but also possesses DUB activity and catalyzes the hydrolysis of isopeptide amide bonds in human proteins which are post-translationally modified with Ub/UBLs,^39^, ^40^ similar to the reported DUB activities of PL^pro^ from other coronaviruses.^11^, ^12^, ^13^, ^41^ Cellular studies reveal that SARS-CoV-2 PL^pro^ preferentially catalyzes the hydrolysis of isopeptide amide bonds C-terminal to ISG15, whereas SARS-CoV PL^pro^ prefers diubiquitin (Ub_2_) substrates over ISG15;^39^, ^40^ both Ub_2_ and ISG15 bind to the active site of PL^pro^ and, additionally, to allosteric sites.^42^, ^43^, ^44^ The apparent selectivity of SARS-CoV-2 PL^pro^ for catalyzing deISG15ylations is precedented by the substrate selectivity of the human DUB ubiquitin specific peptidase 18 (USP18) which also employs a nucleophilic cysteine to selectively catalyze protein deISG15ylations over protein deubiquitinylations.^45^ Notably, the DUB activity of SARS-CoV-2 PL^pro^ is reported to attenuate the host innate immune response via catalyzing the deISG15ylation of ISG15ylated interferon regulatory factor 3 (IRF3), thus potentially enhancing virulence.^39^, ^46^

Reported assays which monitor the DUB activity of PL^pro^ typically employ Ub/UBL derivatives conjugated to a fluorescent group via a C-terminal amide (*e.g.* Ub-AMC or ISG15-AMC; AMC: 7-amino-4-methylcoumarin), the hydrolysis of which can be spectroscopically monitored.^39^, ^40^, ^48^, ^49^ Matrix-assisted laser desorption/ionization (MALDI) mass spectrometry (MS)-based assays, originally developed to monitor the activity of human DUBs,^50^ have also been employed to monitor the DUB activity of PL^pro^; however, the MALDI-MS assay uses Ub_2_ as substrate which is apparently not the preferred PL^pro^ substrate in cells.^51^ The use of substrates which do not bind to the PL^pro^ S′ sites, the relatively high costs associated with use of the Ub-AMC, ISG15-AMC, and Ub_2_ substrates, and/or the use of artificial C-terminal fluorophores limit potential applications of these assays.

**Fig. 1:**
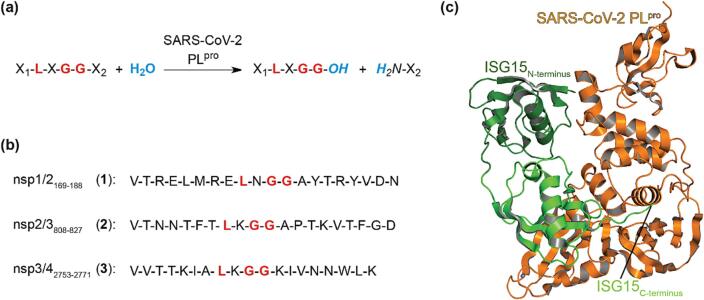
**PL^pro^ catalysis.** (**a**) PL^pro^ catalyzes substrate hydrolysis C-terminal to LXGG motifs; (**b**) sequences of the reported SARS-CoV-2 pp1a/1ab-derived linear oligopeptides nsp1/2_169-188_ (**1**), nsp2/3_808-827_ (**2**), and nsp3/4_2753-2771_ (**3**), previously used in SPE-MS assays; PL^pro^ catalyzed the hydrolysis of **2** substantially more efficiently than that of **1** and **3**.^47^ Residues of the LXGG motif are in red; (**c**) SARS-CoV-2 PL^pro^ (C111S variant, orange) in complex with ISG15 (PDB ID: 7RBS^44^), which is composed of a C- and N-terminal Ub-like domain (light and dark green, respectively), that indicates binding to both the PL^pro^ active site via the C-terminus and the C-terminal Ub-like domain of ISG15 and binding to allosteric sites of PL^pro^ via the N-terminal Ub-like domain (dark green).

We have reported solid-phase extraction coupled to mass spectrometry (SPE-MS)-based assays which directly monitor PL^pro^-catalyzed hydrolysis of oligopeptide fragments derived from the sequence of the Wuhan-Hu-1 strain^52^ of SARS-CoV-2 pp1a/1ab (Fig. 1b). The assay results revealed that PL^pro^ preferentially catalyzes hydrolysis of an oligopeptide which was based on the LXGG motif separating nsp2 and nsp3, *i.e.* nsp2/3_808-827_ (**2**; Fig. 1).^47^ Here we report the results of SPE-MS assays employing *N*^ε^-lysine branched synthetic oligopeptides as substrates, that bind to both the S and S′ sites of PL^pro^, to monitor the DUB activity of isolated recombinant SARS-CoV-2 PL^pro^. The results inform on the substrate requirements for efficient PL^pro^ DUB catalysis and reveal that not only the presence of an LXGG motif but also the entire coding sequence of the substrate which binds in proximity of the active site, in particular that of the UBL, determines the catalytic efficiency of PL^pro^.

## 2 Results and discussion

### 2.1 Assaying the DUB activity of SARS-CoV-2 PL^pro^ using mass spectrometry

SARS-CoV-2 PL^pro^ is reported to attenuate the human innate immune system by catalyzing the deISG15ylation of post-translationally ISG15ylated IRF3;^39^ however, to our knowledge, there are no reports on validation of the DUB activity of isolated recombinant PL^pro^ with oligopeptide substrates. We thus synthesized SPE-MS-compatible *N*^ε^-lysine-branched oligopeptides based on the three reported ISG15ylation sites of IRF3, *i.e.* K193, K360, and K366,^53^ using solid phase peptide synthesis (SPPS). The IRF3-derived peptide fragment was synthesized on a Rink amide resin from the C- to the N-terminus employing Fmoc strategy, *N*-Fmoc lysine with a 4-methyltrityl (Mtt) protected *N*^ε^-amine at the site of the lysine branching, and an N-terminal amino acid with a Boc-protected *N*^α^-amine. Branching was introduced via selective deprotection of the lysine *N*^ε^-Mtt group using 1%_v/v_ trifluoroacetic acid (TFA) and 2%_v/v_ triisopropylsilane (TIPS) in dichloromethane, followed by SPPS to build the ISG15 fragment from C- to N-termini starting with the *N*^ε^-amino group of the IRF3 lysine at the C-terminus. The purified branched oligopeptides were obtained following global deprotection and cleavage from the resin with TFA and HPLC purification (Supporting Figure S1).

The K_193_-branched IRF3_189-197_-ISG15 oligopeptide **4** was incubated with the isolated recombinant PL^pro^ domain of SARS-CoV-2 nsp3 (0.2 µM; enzyme/substrate ratio: 1/10) employing our reported conditions for the PL^pro^-catalyzed hydrolysis of the linear pp1a/1ab-derived oligopeptide nsp2/3_808-827_ (**2**) (50 mM Tris, pH 8.0, 37 °C).^47^ Analysis of the reaction mixture after 14 h incubation by SPE-MS indicated apparent quantitative isopeptide amide bond hydrolysis, as supported by the observed masses of the resultant N- and C-terminal product fragments. Thus, the results confirm the DUB activity of PL^pro^, which has been assigned on the basis of cellular studies and on studies with ISG15 containing a C-terminal fluorophore,^39^, ^40^, ^48^ with an *N*^ε^-lysine-branched oligopeptide substrate.

**Fig. 2:**
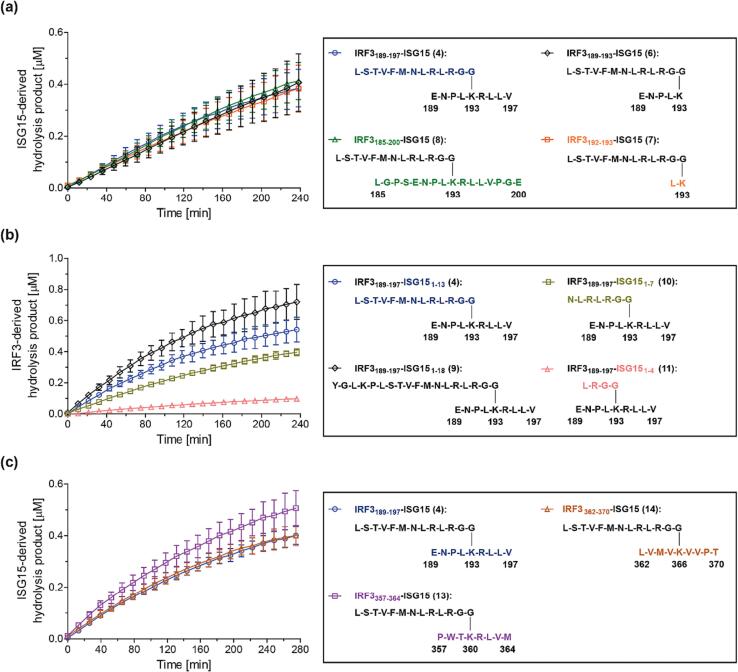
**PL^pro^ catalyzes the hydrolysis of isopeptide amide bonds in *N*^ε^-lysine-branched oligopeptides which mimic ISG15ylated IRF3.** (**a**) Levels of PL^pro^-catalyzed hydrolysis of the K_193_-branched oligopeptides IRF3_189-197_-ISG15 (**4**; blue circles), IRF3_189-193_-ISG15 (**6**; black diamonds), IRF3_192-193_-ISG15 (**7**; orange boxes), and IRF3_185-200_-ISG15 (**8**; green triangles); (**b**) levels of PL^pro^-catalyzed hydrolysis of the K_193_-branched IRF3_189-197_-derived oligopeptides bearing ISG15_1-18_ (**9**; black diamonds), ISG15_1-13_ (**4**; blue circles), ISG15_1-7_ (**10**; olive boxes), and ISG15_1-4_ (**11**; salmon triangles); (**c**) levels of PL^pro^-catalyzed hydrolysis of the K_360_-branched oligopeptide IRF3_357-364_-ISG15 (**13**; lavender boxes) and the K_366_-branched oligopeptide IRF3_362-370_-ISG15 (**14**; brown triangles) compared to that of the K_193_-branched oligopeptide IRF3_189-197_-ISG15 (**4**; blue circles). Conditions: SARS-CoV-2 PL^pro^ (0.2 μM), substrate peptide (2.0 μM), inert *N*-acetylated inert standard peptide (0.2 μM; Ac-LSTVFMNLRLRGG-NH_2_ (**5**) in (**a**) and (**c**), Ac-ENPLKRLLV-NH_2_ (**12**) in (**b**)) in buffer (50 mM Tris, pH 8.0, ambient temperature). Measurement times were normalized to the first sample injection analyzed after the addition of PL^pro^ to the Substrate Mixture (t = 0 s), by which time low levels of substrate hydrolysis were manifest. The inert *N*-acetylated hydrolysis products **5** or **12** were used as an internal standard to quantify hydrolysis (Supporting Figures S2 and S3); SPE-MS assay results are a mean of independent triplicates (n = 3; mean ± standard deviation, SD).

Time course data were subsequently recorded under the same conditions using an automated SPE-MS setup, however, at ambient temperature rather than at 37 °C. Incomplete levels of PL^pro^-catalyzed isopeptide amide bond hydrolysis were observed within ∼240 min incubations under these conditions (∼20 %; Fig. 2a), which may, at least in part, reflect the inability of **4** to efficiently bind to PL^pro^ at allosteric sites as reported for full-length folded (poly)Ub/UBLs.^42^, ^43^, ^44^ The reaction conditions were subsequently varied, with respect to, *e.g.*, buffer composition, pH, and salt additives; the tested variations did not, however, increase the rates of isopeptide amide bond hydrolysis substantially. Note that the corresponding N-terminally *N*-acetylated ISG15-derived hydrolysis product peptide, *i.e.* Ac-LSTVFMNLRLRGG-NH_2_ (**5**), was added as an inert internal standard to the reaction mixture to enable quantification of PL^pro^-catalyzed product formation; the presence of **5** in the reaction mixture did not affect PL^pro^ catalysis, at least substantially (Supporting Figure S2).

Derivatives of the K_193_-branched IRF3_189-197_-ISG15 oligopeptide **4** were synthesized to investigate whether varying the length of the IRF3- or the ISG15-derived fragments affects PL^pro^ catalysis (Fig. 2a and 2b). Interestingly, SPE-MS analysis of the PL^pro^-catalyzed hydrolysis of the oligopeptides **4**, **6**, **7**, and **8** revealed that varying the length of the IRF3-derived fragment did not affect catalysis substantially, though the hydrolysis of the K_193_-branched IRF3_185-200_-ISG15 oligopeptide **8**, which has the relatively largest IRF3-derived fragment of the tested oligopeptides, appeared to be slightly favored (Fig. 2a). By contrast, varying the length of the ISG15-derived fragment had a substantial effect on PL^pro^ catalysis; the oligopeptide **9** with the relatively largest ISG15-derived fragment bearing 18 C-terminal amino acids was hydrolyzed substantially more efficiently than oligopeptide **11** with the relatively smallest ISG15-derived fragment bearing only the essential C-terminal LRGG tetrad (Fig. 2b). Levels of PL^pro^-catalyzed substrate hydrolysis appear to increase with increasing length of the ISG15-derived fragment peptide; the rank order of PL^pro^ substrate preference was determined by quantifying product formation relative to Ac-ENPLKRLLV-NH_2_ (**12**), which was used as an internal standard in the reactions. The combined oligopeptide results indicate that the binding of the *N*^ε^-lysine-branched oligopeptides to the S′ sites is less important for catalysis than binding to the S sites. The situation may, however, be different with full-length folded protein substrates. Note that either the corresponding N-terminally *N*-acetylated ISG15-derived hydrolysis product peptide, *i.e.* Ac-LSTVFMNLRLRGG-NH_2_ (**5**), or the corresponding N-terminally *N*-acetylated IRF3_189-197_-derived hydrolysis product peptide, *i.e.* Ac-ENPLKRLLV-NH_2_ (**12**), was added as an internal standard to the reaction mixtures to enable quantification of PL^pro^-catalyzed product formation; the presence of **5** or **12** in the reaction mixture did not affect PL^pro^ catalysis, at least substantially (Supporting Figures S2 and S3).

The K_360_-branched IRF3_357-364_-ISG15 peptide **13** and the K_366_-branched IRF3_362-370_-ISG15 peptide **14** were synthesized and tested to investigate the potential of isolated recombinant PL^pro^ to catalyze regioselective deISG15ylations of post-translationally modified IRF3. Oligopeptides **4**, **13**, and **14** were incubated with PL^pro^ in the presence of the internal standard **5**, and isopeptide amide bond hydrolysis was monitored using SPE-MS (Fig. 2c). The results reveal that the sequences of the three tested IRF3-derived peptide fragments, which likely bind to the S′ sites of PL^pro^, do not affect the rate of peptide hydrolysis substantially, in accord with prior results which showed that the length of the IRF3-derived peptide fragment affects PL^pro^ catalysis less substantially than the length of the ISG15-derived peptide fragment (Fig. 2a and 2b). The observation that PL^pro^ may catalyze the deISG15ylation of oligopeptide **13** slightly more efficiently than that of **4** and **14** might be a result of the comparatively lower purity of **13** compared to that of all other tested peptides (Supporting Figure S1). Note that PL^pro^ may catalyze the regioselective deISG15ylation of full-length folded IRF3 in a cellular context.

### 2.2 The primary sequence of the PL^pro^ substrates affects catalysis

To investigate whether the substrate preference of SARS-CoV-2 PL^pro^ depends on the primary sequence of the UBLs, we synthesized the oligopeptide derivatives of K_193_-branched IRF3_189-197_-ISG15 **4**, in which the ISG15-fragment was substituted for the corresponding first 13 C-terminal amino acids of Ub (IRF3_189-197_-Ub, **15**) or the UBLs NEDD8 (IRF3_189-197_-NEDD8, **16**), URM1 (IRF3_189-197_-URM1, **17**), and small ubiquitin-related modifier 1 (SUMO1; IRF3_189-197_-SUMO1, **18**), the latter of which does not contain a C-terminal LXGG motif and should hence not be a substrate of PL^pro^ (Supporting Figure S1). K_193_-branched IRF3_189-197_ derivatives were attractive synthesis targets because the ubiquitinylation at K193 had been reported^54^; the corresponding NEDD8, URM1, and SUMO1 derivatives of **4** were also synthesized despite their unclear biological relevance. The length of the UBL fragment was kept constant to help enable comparison, as PL^pro^ catalyzes the hydrolysis of derivatives of **4**, in which the length of the ISG15 fragment was varied, with different efficiencies (Fig. 2b).

**Fig. 3:**
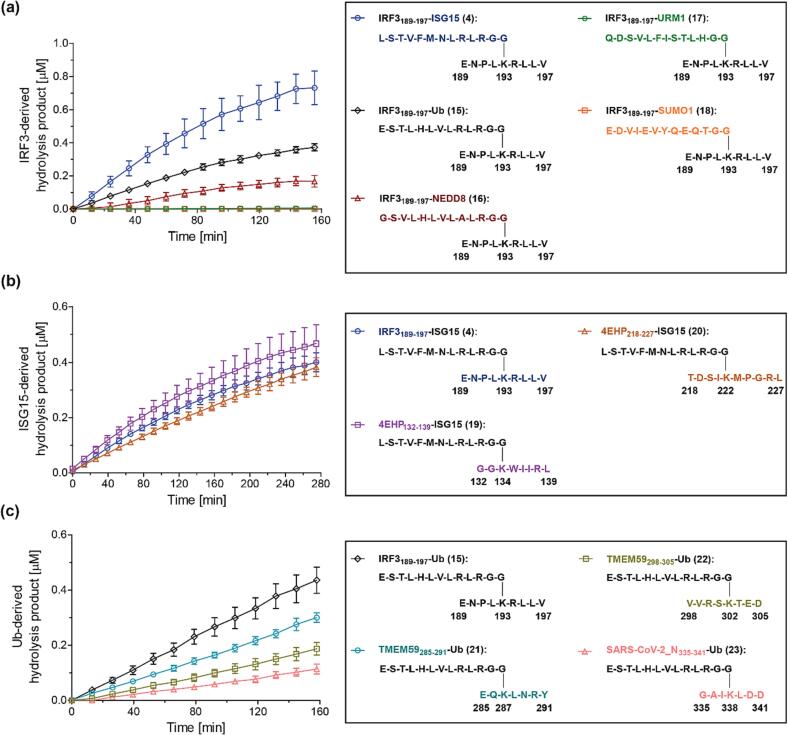
**PL^pro^ catalyzes the hydrolysis of isopeptide amide bonds in *N*^ε^-lysine-branched oligopeptides that mimic post-translational protein modification by Ub/UBLs.** (**a**) Levels of PL^pro^-catalyzed hydrolysis of oligopeptides derived from K_193_-branched IRF3_189-197_ with ISG15 (**4**; blue circles), Ub (**15**; black diamonds), NEDD8 (**16**; red triangles), URM1 (**17**; green hexagons), and SUMO1 (**18**; orange boxes). Note that SUMO1 does not have an LXGG motif at its C-terminus; (**b**) levels of PL^pro^-catalyzed hydrolysis of the K_134_-branched oligopeptide 4EHP_132-139_-ISG15 (**19**; lavender boxes) and the K_222_-branched oligopeptide 4EHP_218-227_-ISG15 (**20**; brown triangles) compared to that of the K_193_-branched oligopeptide IRF3_189-197_-ISG15 (**4**; blue circles); (**c**) levels of PL^pro^-catalyzed hydrolysis of oligopeptides derived from the *N*^ε^-lysine-branched IRF3_189-197_-Ub (**15**; black diamonds), TMEM59_285-291_-Ub (**21**; teal circles), TMEM59_298-305_-Ub (**22**; olive boxes), and SARS-CoV-2_N_335-341_-Ub (**23**; salmon triangles) peptides. Conditions: SARS-CoV-2 PL^pro^ (0.2 μM), substrate peptide (2.0 μM), inert *N*-acetylated standard peptide (0.2 μM; Ac-ENPLKRLLV-NH_2_ (**12**) in (**a**), Ac-LSTVFMNLRLRGG-NH_2_ (**5**) in (**b**), and Ac-ESTLHLVLRLRGG-NH_2_ (**24**) in (**c**)) in buffer (50 mM Tris, pH 8.0, ambient temperature). Measurement times were normalized to the first sample injection analyzed after the addition of PL^pro^ to the Substrate Mixture (t = 0 s), by which time low levels of substrate hydrolysis were manifest. The inert *N*-acetylated hydrolysis product **5**, **12** or **24** was used as an internal standard to quantify hydrolysis (Supporting Figures S2-S4); SPE-MS assay results are a mean of independent triplicates (n = 3; mean ± SD).

The oligopeptides **4**, **15**, **16**, **17**, and **18** were incubated with isolated recombinant SARS-CoV-2 PL^pro^; hydrolysis of their isopeptide amide bond was monitored in the presence of the corresponding inert N-terminally *N*-acetylated IRF3_189-197_-derived hydrolysis product peptide Ac-ENPLKRLLV-NH_2_ (**12**) using SPE-MS (Fig. 3a). The results revealed that PL^pro^ catalyzes the hydrolysis of the IRF3_189-197_-ISG15 (**4**) isopeptide amide bond more efficiently than those of IRF3_189-197_-Ub (**15**) and, in particular, IRF3_189-197_-NEDD8 (**16**) (Fig. 3a). However, **4**, **15**, and **16** were substantially more efficient PL^pro^ substrates than IRF3_189-197_-URM1 (**17**). As anticipated, IRF3_189-197_-SUMO1 (**18**) was not a substrate for isolated recombinant PL^pro^ under the tested conditions, an observation which likely reflects the lack of an LXGG motif in **18**, which instead has a QTGG motif that cannot interact efficiently with the S1-S4 sites of PL^pro^ (*vide infra*).

IRF3_189-197_-ISG15 (**4**), IRF3_189-197_-Ub (**15**), and IRF3_189-197_-NEDD8 (**16**) all have an LRGG motif, whereas IRF3_189-197_-URM1 (**17**) has an LHGG motif which may rationalize why it is a substantially poorer substrate than **4**, **15**, and **16** (Fig. 3a). Nonetheless, the assay results clearly show that the different hydrolysis rates of **4**, **15**, and **16** are not dependent on the presence of a LRGG motif or an IRF3_189-197_ fragment, suggesting that the primary/coding sequence of the UBL N-terminal to the LXGG motif is important in PL^pro^ catalysis.

Interestingly, the results with **4**, **15**, and **16** reflect reported SARS-CoV-2 PL^pro^ substrate preferences observed in cellular studies which also showed that PL^pro^-catalyzed protein deISG15ylations are more efficient than the corresponding deubiquitinylations, which are both more efficient than deNEDD8ylations.^39^, ^40^ Thus, the combined evidence suggests that not only the substrate fold affects PL^pro^ catalysis, *inter alia* via binding to allosteric sites, but also, in particular, the sequence identity of the amino acid fragment N-terminal to the LXGG motif of the substrate.

In general, the ISG15ylation sites of human proteins are poorly characterized, in part, likely because the standard workflow to localize protein ubiquitinylation sites, *i.e.* protein denaturation followed by tryptic digestion and analysis of the resultant peptide fragments by MS/MS, affords the same lysine-branched peptide fragments for both ubiquitinylated and ISG15ylated proteins. Thus, a different workflow is required, *e.g.* involving digestion using non-standard proteases with different substrate selectivities than trypsin.^35^, ^55^, ^56^, ^57^, ^58^ Apart from ISG15ylation sites of IRF3, the ISG15ylation sites of the eIF4E-homologous protein (4EHP) have been characterized, *i.e.* at K134 and K222.^59^ 4EHP binds to the mRNA 5′-cap structure and suppresses translation by competing with the eukaryotic initiation factor 4E (eIF4E) for binding to the cap structure. The ISG15ylation of 4EHP is proposed to control translation during immune responses;^59^ note that the translation initiation factor eIF4G is a reported substrate of rhinovirus 2A^pro^ and of the foot-and-mouth-disease virus (FMDV) leader protease (L^pro^), suggesting that modulation of protein translation may be a general strategy of *Riboviria* to evade the host immune system.^60^

The K_134_-branched 4EHP_132-139_-ISG15 peptide **19** and the K_222_-branched 4EHP_218-227_-ISG15 peptide **20** were synthesized to investigate the potential of isolated recombinant SARS-CoV-2 PL^pro^ to catalyze selective deISG15ylations of post-translationally modified 4EHP. Oligopeptides **4**, **19**, and **20** were incubated with PL^pro^ in the presence of the inert internal standard **5**, and isopeptide amide bond hydrolysis was monitored using SPE-MS (Fig. 3b). The results reveal that the rates of PL^pro^-catalyzed peptide hydrolyses for **4**, **19**, and **20** are similar, within experimental error (Fig. 3b). This observation is in accord with the previous results showing that PL^pro^ catalyzes the deISG15ylation of IRF3-derived fragment peptides regardless of the site of ISG15ylation (Fig. 2c) and thus supports the proposal that the substrate binding in the S sites of PL^pro^ is more important for efficient catalysis than substrate binding in the S′ sites of PL^pro^, in accord with reported studies on the substrate efficiency of oligopeptides based on SARS-CoV-2 nsps.^61^ Note that the observed variation in the absolute efficiency of PL^pro^ catalysis appeared to depend on the batch of PL^pro^; hence, we determined the rank order of substrate preference by quantifying product formation relative to internal standards, the rank order did not depend on the batch of PL^pro^. The results suggest that ISG15ylated 4EHP may be a substrate of PL^pro^ in cells.

### 2.3 N^ε^-Lysine-branched oligopeptides may help enable the identification of PL^pro^ substrates

To further dissect the effect of the UBL fragment of the tested *N*^ε^-lysine-branched oligopeptides, which binds to the PL^pro^ S sites, on the rate of isopeptide amide bond hydrolysis from that of the fragment which binds to the PL^pro^ S′ sites, and to investigate the potential of synthetic *N*^ε^-lysine-branched oligopeptides for the identification of SARS-CoV-2 PL^pro^ substrates, we synthesized a set of *N*^ε^-lysine-branched oligopeptides which mimic protein ubiquitinylation, *i.e.* via employing the 13 C-terminal residues of Ub. In general, the sites of protein (poly)ubiquitinylations have been characterized in greater detail than the sites of protein ISG15ylations. Potential SARS-CoV-2 PL^pro^ substrates were chosen on the basis of reported proteomic studies which showed, *e.g.*, that K338 of the SARS-CoV-2 nucleocapsid (N) protein can be ubiquitinylated.^62^ Reduced levels of ubiquitinylation of the autophagy-related human transmembrane protein TMEM59 (at *e.g.* K287 and K302) have been associated with SARS-CoV-2 infections, which may suggest that TMEM59 could be a substrate of SARS-CoV-2 PL^pro^.^62^ We thus synthesized the corresponding K_287_-branched TMEM59_285-291_-Ub peptide **21**, the K_302_-branched TMEM59_298-305_-Ub peptide **22**, and the K_338_-branched SARS-CoV-2_N_335-341_-Ub peptide **23** (Supporting Figure S1).

The oligopeptides **15**, **21**, **22**, and **23** were incubated with isolated recombinant SARS-CoV-2 PL^pro^ and hydrolysis of their isopeptide amide bond was monitored using SPE-MS (Fig. 3c). Note that the corresponding N-terminally *N*-acetylated Ub-derived hydrolysis product peptide, *i.e.* Ac-ESTLHLVLRLRGG-NH_2_ (**24**; Supporting Figure S1), was added as an inert internal standard to the reaction mixtures to enable quantification of PL^pro^-catalyzed product formation; the presence of **24** in the reaction mixture did not affect PL^pro^ catalysis substantially (Supporting Figure S4). The results reveal that SARS-CoV-2 PL^pro^ catalyzes the hydrolysis of the IRF3_189-197_-Ub (**15**) isopeptide amide bond more efficiently than those of **21**, **22**, and **23** (Fig. 3c). The PL^pro^-catalyzed isopeptide hydrolysis in IRF3_189-197_-Ub (**15**) was ∼2-fold more efficient as in SARS-CoV-2_N_335-341_-Ub (**23**), which was the least efficient substrate of the four substrates tested. This observation indicates that PL^pro^ catalysis can be affected by substrate binding to the S′ sites and, by implication, also by the substrate fold in proximity of the S′ sites, by contrast to previous results with peptide fragments mimicking the ISG15ylation of IRF3 and 4EHP (Fig. 2).

The combined results suggest that PL^pro^ may be promiscuous (including compared to M^pro^) with respect to its *in vivo* protein substrates, and that it likely has the capability to catalyze a broad range of deISG15ylation and deubiquitinylation reactions of both host and viral proteins. The results also highlight the potential of *N*^ε^-lysine-branched oligopeptides to help enable identification of PL^pro^ substrates, including post-translationally modified viral proteins.

### 2.4 Single amino acid substitutions in PL^pro^ substrates can affect catalysis

**Fig. 4:**
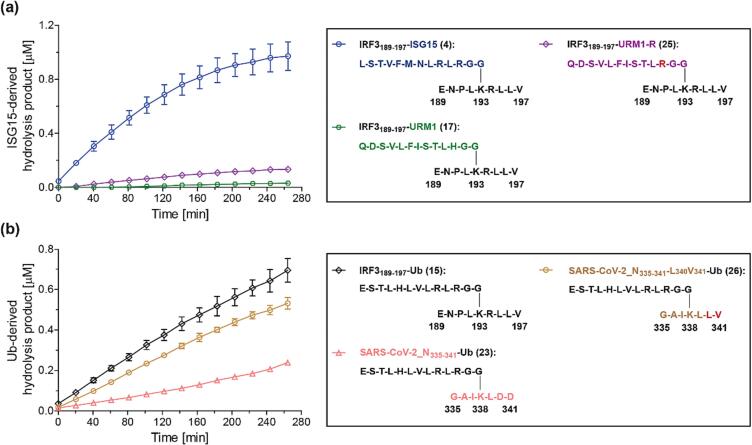
**The primary sequence of *N*^ε^-lysine-branched oligopeptides affects the efficiency of PL^pro^ catalysis.** (**a**) PL^pro^-catalyzed hydrolysis of the H-to-R variant of the K_193_-branched IRF3_189-197_-URM1 peptide **17** (green hexagons), *i.e.***25** (lavender diamonds), compared to that of IRF3_189-197_-ISG15 (**4**; blue circles); (**b**) PL^pro^-catalyzed hydrolysis of SARS-CoV-2_N_335-341_-Ub_L_340_V_341_ (**26**; brown circles) compared to that of SARS-CoV-2_N_335-341_-Ub (**23**; salmon triangles) and IRF3_189-197_-Ub (**15**; black diamonds). Substituted residues are in red. Conditions: SARS-CoV-2 PL^pro^ (0.2 μM), substrate peptide (2.0 μM), inert *N*-acetylated standard peptide (0.2 μM; Ac-ENPLKRLLV-NH_2_ (**12**) in (**a**), Ac-ESTLHLVLRLRGG-NH_2_ (**24**) in (**b**)) in buffer (50 mM Tris, pH 8.0, ambient temperature). Measurement times were normalized to the first sample injection analyzed after the addition of PL^pro^ to the Substrate Mixture (t = 0 s), by which time low levels of substrate hydrolysis were manifest. The inert *N*-acetylated hydrolysis products were used as internal standard to quantify hydrolysis (Supporting Figures S3 and S4); SPE-MS assay results are a mean of independent triplicates (n = 3; mean ± SD).

The observation that IRF3_189-197_-ISG15 (**4**), IRF3_189-197_-Ub (**15**), and IRF3_189-197_-NEDD8 (**16**) all have an LRGG motif, whereas IRF3_189-197_-URM1 (**17**) has an LHGG motif and, relative to **4**, **15**, and **16**, is a substantially poorer PL^pro^ substrate raises the question as to whether PL^pro^ catalyzes the hydrolysis of the corresponding IRF3_189-197_-URM1-R (**25**) variant of **17**, in which the LHGG histidine residue has been substituted for an arginine residue, more efficiently than that of **17** (Fig. 3a). **25** was thus synthesized (Supporting Figure S1) and incubated with isolated recombinant PL^pro^, and its hydrolysis was monitored using SPE-MS (Fig. 4a).

Although the PL^pro^-catalyzed hydrolysis of **25** was ∼4-fold more efficient than that of **17** (after 4 h), it remained ∼7-fold less efficient compared to the PL^pro^-catalyzed hydrolysis of IRF3_189-197_-ISG15 (**4**) (after 4 h), indicating that peptides with an LRGG motif are, in principle, better substrates than those with an LHGG motif (Fig. 4a). However, considering that **25** and **4** are based on the identical IRF3_189-197_ fragment C-terminal to their LRGG motif which likely binds to the S′ sites of PL^pro^ in an identical manner, the results support the proposal that the primary sequence of the substrate N-terminal to the LXGG motif can substantially affect catalysis. This observation may reflect the importance of substrate binding to S sites other than S1-S4, including at allosteric positions, and/or the presence of secondary structural elements in the substrate that favor PL^pro^ catalysis. Note that substrate binding to allosteric sites by full-length folded substrate proteins has been proposed to be important in PL^pro^ catalysis on the basis of crystallographic studies.^42^, ^43^, ^44^

The PL^pro^-catalyzed hydrolysis of TMEM59_298-305_-Ub (**22**) and SARS-CoV-2_N_335-341_-Ub (**23**), which bear an acidic ED or DD sequence, respectively, in the sequence C-terminal to their LXGG motif, is less efficient compared to that of IRF3_189-197_-Ub (**15**) (Fig. 3c), which bears a hydrophobic LV sequence at the corresponding position. This observation raises the possibility that acidic residues proximate to the substrate P1′ lysine residue involved in the isopeptide amide bond, are detrimental for PL^pro^ catalysis. To test this proposal, we synthesized the corresponding SARS-CoV-2_N_335-341_-Ub_L_340_V_341_ variant (**26**; Supporting Figure S1), in which the DD sequence was substituted for an LV sequence as present in **15**, and monitored its PL^pro^-catalyzed hydrolysis using SPE-MS (Fig. 4b). The results reveal that PL^pro^ catalyzes the hydrolysis of **26** with similar efficiency as that of **15** and ∼2.5-fold more efficiently than that of **23** (after 4 h), supporting the proposal that hydrophobic residues may be preferred in proximity of the substrate lysine residue involved in the isopeptide amide bond, potentially due to improved binding to the PL^pro^ S′ sites.

The combined results clearly indicate that the primary sequence of oligopeptides affects SARS-CoV-2 PL^pro^ catalysis and that apparently minor changes in their sequence, which may be distal to the LXGG motif, can have a pronounced effect on catalysis. It thus appears that substrate binding to both the S and S′ sites of PL^pro^ has potential to modulate catalysis.

### 2.5 Substrate competition studies

To investigate whether *N*^ε^-lysine-branched oligopeptides or the reported pp1a/1ab-derived linear oligopeptide nsp2/3_808-827_ (**2**) are more efficient substrates of SARS-CoV-2 PL^pro^, we attempted to determine ${\text{k}}_{\text{cat}}$/${\text{K}}_{\text{m}}$-values. These efforts were, however, unsuccessful due to technical limitations of the SPE-MS assay, *i.e.* high peptide concentrations saturated the sensor of the mass spectrometer impeding the quantification of PL^pro^ catalysis. As an alternative, direct substrate competition studies were performed using the linear pp1a/1ab-derived oligopeptide nsp2/3_808-827_ (**2**) and the two lysine-branched oligopeptide PL^pro^ substrates IRF3_189-197_-ISG15 (**4**) or IRF3_189-197_-Ub (**15**). Note that nsp2/3_808-827_ was employed as a pp1a/1ab-derived linear substrate because it was a more efficient substrate compared to linear peptides based on the nsp1/2 or nsp3/4 cleavage site,^47^ in accord with studies that employed a LCMS assay to investigate the substrate preference of SARS-CoV-2 PL^pro^.^63^

**Fig. 5:**
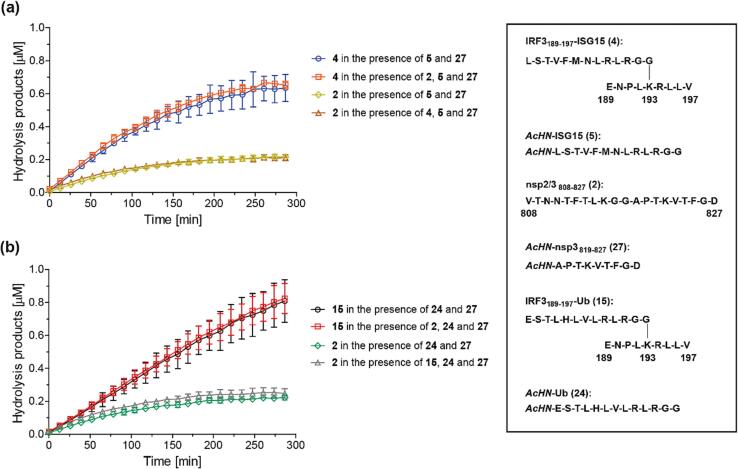
***N*^ε^-Lysine-branched oligopeptides are more efficient substrates of isolated recombinant PL^pro^ than linear oligopeptides.** (**a**) The SARS-CoV-2 PL^pro^-catalyzed hydrolysis of IRF3_189-197_-ISG15 (**4**) is not affected by the presence of nsp2/3_808-827_ (**2**) and vice versa; **4** appears to be a more efficient PL^pro^ substrate than **2**; (**b**) the SARS-CoV-2 PL^pro^-catalyzed hydrolysis of IRF3_189-197_-Ub (**15**) is not affected by the presence of nsp2/3_808-827_ (**2**) and vice versa; **15** appears to be a more efficient PL^pro^ substrate than **2**. Conditions: SARS-CoV-2 PL^pro^ (0.2 μM), substrate peptide(s) (2.0 μM), inert *N*-acetylated standard peptides (0.2 μM, as indicated) in buffer (50 mM Tris, pH 8.0, ambient temperature). Measurement times were normalized to the first sample injection analyzed after the addition of PL^pro^ to the Substrate Mixture (t = 0 s), by which time low levels of substrate hydrolysis were manifest. The inert *N*-acetylated hydrolysis products **27**^47^ and **5** or **24** were used as internal standard to quantify hydrolysis (Supporting Figures S2 and S4); SPE-MS assay results are a mean of independent triplicates (n = 3; mean ± SD).

Equimolar amounts of nsp2/3_808-827_ (**2**) and either IRF3_189-197_-ISG15 (**4**) or IRF3_189-197_-Ub (**15**) were incubated with isolated recombinant SARS-CoV-2 PL^pro^ in the same reaction vessel, together with the corresponding inert *N*-acetylated hydrolysis product peptides of **2** and **4** or **15** (as internal standards). PL^pro^-catalyzed peptide hydrolysis was monitored using SPE-MS, which was suitable for these experiments provided that substrates and products have different masses.^64^ The combined results reveal that PL^pro^-catalyzed hydrolysis of **2** and **4**/**15** does not affect each other under the tested conditions, *i.e.* the PL^pro^-catalyzed hydrolysis of **2** proceeds as efficiently in the presence of **4** or **15** as in its absence, and vice versa (Fig. 5). This observation may indicate that, under the tested conditions, the initial binding of the peptides to PL^pro^ is not rate-limiting regardless of whether peptides are branched or linear, *i.e.* the on/off-rates for peptide binding are faster than the peptide hydrolysis rates, potentially because the peptides lack appropriate folding.

The results also reveal that the PL^pro^-catalyzed hydrolysis of nsp2/3_808-827_ (**2**) is less efficient than that of **4** or **15**, *i.e.* ∼10 % of **2** was observed to be hydrolyzed after 5 h whereas ∼35–40 % of **4** and **15** were observed to be hydrolyzed after 5 h which may indicate that the ${\text{k}}_{\text{cat}}$/${\text{K}}_{\text{m}}$-values of the *N*^ε^-lysine-branched oligopeptides are higher than that of **2**. Notably, it appears that the absolute levels of PL^pro^ catalysis depended on the batch of PL^pro^ and substrate sequence used.

### 2.6 Conformational analysis of substrate binding

To investigate the binding mode of *N*^ε^-lysine-branched oligopeptides to PL^pro^, we computationally modelled nine non-covalently bound PL^pro^:oligopeptide complexes, including the IRF3_189-197_ oligopeptide *N*^ε^-branched with ISG15 (**4**), Ub (**15**), NEDD8 (**16**), URM1 (**17**) or SUMO1 (**18**), as well as the TMEM59_285-291_ (**21**), TMEM59_298-305_ (**22**), and SARS-CoV-2_N_335-341_ (**23**) oligopeptides, all of which are branched with Ub via an *N*^ε^-lysine. The models were based on a reported PL^pro^ structure (PDB ID: 6WX4^65^), using two of the reported AutoDock CrankPep (ADCP)^66^-docked poses of the nsp2/3_808-827_ oligopeptide **2**^61^ as templates for modelling the N- and C-terminal fragments (Supporting Figures S5-S7). For **17**, we modelled both the neutral (^N^) and positive charged (^+^) states of the P3 histidine imidazole group, as PL^pro^ has a reported preference for positively charged residues at this position.^65^ The resultant PL^pro^:oligopeptide complexes were subjected to 3 × 200 ns molecular dynamics (MD) simulations, and the stability of the complexes was assessed by calculating the root mean square deviations (RMSDs) and root mean square fluctuations (RMSFs) (Supporting Figures S8-S17).

Analysis of the backbone RMSFs of the PL^pro^-bound *N*^ε^-lysine-branched oligopeptides reveals that the P6-P1 residues of the UBL-derived peptide fragment are stable, with values <3 Å (Supporting Figure S14); an exception was IRF3_189-197_-URM1 (**17**) with a neutral P3 histidine imidazole, an observation suggesting that the protonation state of this group may affect complex stability. RMSF analysis of the oligopeptide sidechains reveals that the conformation of the P4 residue is rigid relative to its adjacent P5 and P3 residues (Supporting Figure S15), likely reflecting the conserved nature of the LXGG motif leucine residue which binds to the PL^pro^ S4 site. Notably, the conformation of the P4 glutamine residue of the IRF3_189-197_-SUMO1 oligopeptide (**18**), which is not a substrate of isolated recombinant PL^pro^ and which lacks the LXGG motif (Fig. 3a), is also rigid, suggesting that complex stability itself may not necessarily indicate productive substrate recognition.

**Fig. 6:**
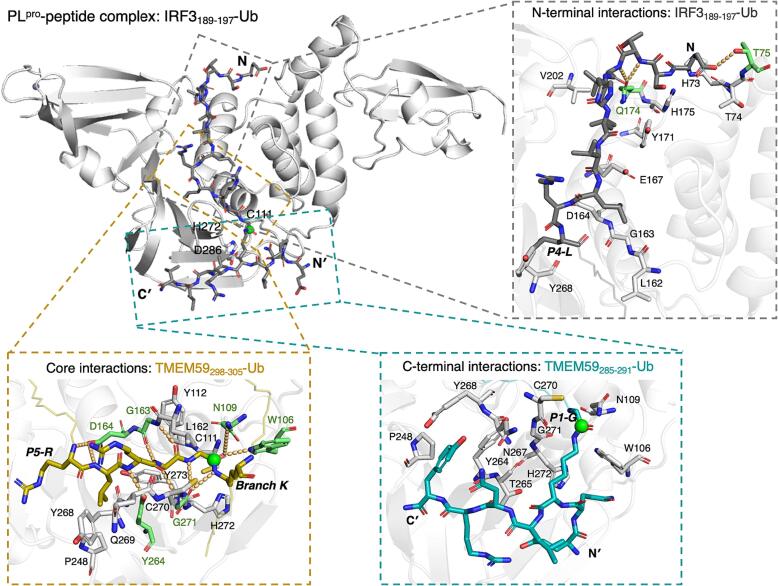
**Modelled interactions of PL**^**pro**^**with lysine-branched oligopeptides.** Views of clustering-derived representative PL^pro^:oligopeptide complex structures exemplifying the overall binding mode of the oligopeptides and their interactions with PL^pro^ N-terminal and C-terminal to the S4-S1 sites. Peptides are colored as in Fig. 3, with the P1 scissile amide carbons as green spheres; the N-terminus of the UBL-derived fragment and the N- and C-termini of the S′ binding fragment are labelled N, N′, and C′, respectively. PL^pro^ residues interacting via hydrogen bonding with the oligopeptide are in lime (the calculated occurrence is ≥25%; orange dashes; not necessarily present in the frame). PL^pro^ residues within 4 Å of the peptide residues that are calculated to contribute ≥0.5 kcal mol^−1^ binding energy, are in white.

Conserved hydrogen bonding interactions appear to stabilize binding of the oligopeptide backbone to the PL^pro^ S4-S1 sites (Fig. 6, Supporting Figures S18 and S19), reminiscent of the interactions observed in the modelled PL^pro^:nsp2/3_808-827_ (**2**) complex.^61^ Hydrogen bonding interactions involving residues of the UBL-derived peptide fragment N-terminal to P4-P1 may also contribute to binding, *e.g.* with T75 and Q174; however, they appear to be less conserved than those with the P4-P1 LXGG motif (Supporting Table S1, Fig. 6, Supporting Figures S18 and S19), consistent with previous observations on the modelled interactions of pp1a/1ab-derived linear oligopeptides with PL^pro^.^61^ Notably, the IRF3_189-197_-SUMO1 oligopeptide (**18**) can apparently engage in similar interactions with PL^pro^ as the other modelled *N*^ε^-lysine-branched oligopeptides; however, it appears that its P4 glutamine sidechain binds to the S4 site less efficiently than a leucine sidechain (Supporting Figures S20-S24).

In all the modelled PL^pro^:substrate complexes, the residues C-terminal to the substrate LXGG motif manifest high conformational flexibility, with backbone RMSFs exceeding 4 Å (Supporting Figure S16). The V-shaped RMSF plots indicate that the peptide backbone is flexible at both its N- and C-termini (Supporting Figure S16). Nonetheless, transient hydrogen bonding and dispersion interactions were observed between the substrates and *e.g.* N109 of PL^pro^ (Supporting Table S1), as well as with residues around Y268 which form a flexible β-hairpin loop (G266-G271), *i.e.* the blocking loop 2 (BL2), that controls substrate access to the active site.^67^, ^68^, ^69^, ^70^ In the apo form of PL^pro^, the BL2 loop is present in an open conformation; substrate binding likely triggers an induced fit mechanism resulting in closure of the BL2 loop, in a manner helping to orient the C-terminus of the UBL productively towards the active site.^70^ The conformational changes in the BL2 loop associated with substrate binding to PL^pro^ are conserved in, at least, some human DUBs, suggesting that both viral and human DUBs employ similar mechanisms to regulate substrate selectivity.^71^, ^72^ Note that SARS-CoV and SARS-CoV-2 PL^pro^ inhibitors can also bind proximate to BL2 and alter or stabilize its conformation,^39^, ^67^, ^70^, ^73^, ^74^, ^75^, ^76^ as precedented by the inhibitor-induced conformational changes in BL2 of human DUBs, such as USP7 and USP14.^77^

**Fig. 7:**
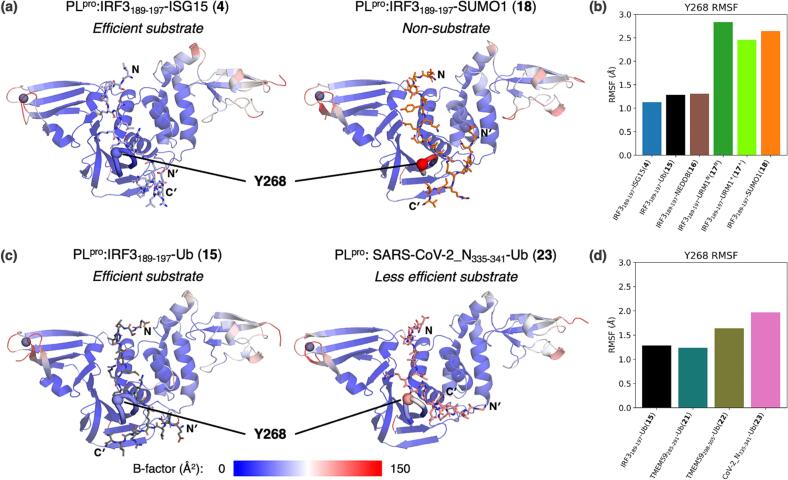
**BL2 flexibility in PL^pro^:oligopeptide complexes is indicative of poor hydrolysis efficiency.** Views of clustering-derived representative structures of (**a**) the PL^pro^:IRF3_189-197_-ISG15 (**4**) and PL^pro^:IRF3_189-197_-SUMO1 (**18**) complexes and (**c**) the PL^pro^:IRF3_189-197_-Ub (**15**) and PL^pro^:SARS-CoV-2_N_335-341_-Ub (**23**) complexes. PL^pro^ residues are colored based on MD-derived backbone B-factors; red represents high B-factors, which is indicative of relatively high residue flexibility. The bar plots show comparisons of Y268 backbone RMSF for PL^pro^ in complex with (**b**) oligopeptides **4**, **15**, **16**, **17**, and **18**, and (**d**) oligopeptides **15**, **21**, **22**, and **23**. Residues forming BL2 (G266-G271) are shown as a thickened loop, with Y268 Cα being shown as a sphere. The N-terminus of the UBL-derived fragment and the N- and C-termini of the S′ binding fragment are labelled N, N′, and C′, respectively.

Our previous modelling studies have shown that the BL2 conformation is stable in its closed form in the PL^pro^:nsp2/3_808-827_ (**2**) substrate complex, though alternating between open and closed forms with apo PL^pro^, or when the active site is occupied by a peptide that is not efficiently hydrolyzed.^61^ We analyzed BL2 dynamics in all the nine modelled PL^pro^:substrate complexes by backbone RMSF of the most flexible residue in BL2 (*i.e.* Y268) (Fig. 7, Supporting Figures S25 and S26) and the Y268-P248 Cα-Cα distance (Supporting Figures S27 and S28); in the latter case, a distance of >12 Å was considered to be indicative of an open BL2 conformation. The results reveal that BL2 remains stably closed in the PL^pro^:IRF3_189-197_-ISG15 (**4**) and PL^pro^:IRF3_189-197_-Ub (**15**) complexes (Fig. 7), which are the most efficient substrates among the modelled peptides (Fig. 3). By contrast, BL2 is flexible in the modelled PL^pro^ complex with the relatively inefficient substrate IRF3_189-197_-URM1 (**17**), regardless of the charge state of the P3 histidine imidazole group. Similarly, in the modelled PL^pro^:IRF3_189-197_-SUMO1 (**18**) complex, BL2 frequently opens and closes (Supporting Figure S28); note that **18** is not a PL^pro^ substrate (Fig. 3). The latter observation is likely a result of the P4 leucine to glutamine substitution in **18**; consistent with this proposal, previous reports have shown that the binding of hydrophobic sidechains to the PL^pro^ S4 site induces BL2 closure.^65^, ^70^, ^78^, ^79^

In the modelled PL^pro^ complexes with the *N*^ε^-lysine-branched oligopeptides that mimic post-translational protein ubiquitinylation (*i.e.* **15**, **21**, **22**, and **23**), BL2 remains rigidly closed for the relatively more efficient substrates (*i.e.* **15** and **21**), as observed for the pp1a/1ab-derived nsp2/3_808-827_ peptide **2**,^61^ but not for the less efficient substrates (*i.e.* **22** and **23**) (Fig. 7, Supporting Figures S27 and S28). These observations are in accord with the results on the stability of the complexes of PL^pro^ and *N*^ε^-lysine-branched oligopeptides mimicking post-translational protein ISG15ylation. In particular, it appears that hydrophobic residues C-terminal to the P1′ lysine residue can affect BL2 flexibility (Supporting Figures S13, S23, S24, and S29-S31), in accord with the experimentally observed preference of PL^pro^ for hydrophobic residues C-terminal to the substrate P1′ lysine residue involved in the isopeptide amide bond (Fig. 4).

The combined modelling and experimental results reveal the importance of the conformational dynamics of the BL2 loop; it not only enables productive substrate binding in the active site, but also governs substrate selectivity. Importantly, the BL2 flexibility and opening tendency observed in the MD simulations correlate with the experimentally observed substrate selectivity, *i.e.* conformational BL2 flexibility in the PL^pro^:substrate complex appears to result in inefficient catalysis (Fig. 7). Although substrate peptide dissociation was not observed over the course of the MD simulations, the observations suggest that a sustained closed state of BL2 may be necessary for initiating productive catalysis, potentially because BL2 is proximal to H272 which is part of the catalytic triad.^68^ Thus, the observation that the PL^pro^-catalyzed hydrolysis of the linear pp1a/1ab-derived oligopeptide nsp2/3_808-827_ (**2**) and the *N*^ε^-lysine-branched oligopeptide substrates do not appear to affect each other when incubated in the same reaction vessel (Fig. 5), implies that inefficient substrate binding to PL^pro^ results in dissociation being faster than substrate hydrolysis.

## 3 Conclusions

SARS-CoV-2 PL^pro^ catalyzes the hydrolysis of peptide amide bonds that are C-terminal to three LXGG motifs in pp1a/1ab to release functional nsps1-3.^1^, ^2^ By contrast with M^pro^, PL^pro^ is also a deubiquitinase (DUB) that catalyzed the hydrolysis of isopeptide amide bonds of human proteins that are post-translationally modified with Ub/UBLs, in particular ISG15.^39^, ^40^ Thus, the DUB substrate preference of PL^pro^ is apparently similar to that of the human ubiquitin specific peptidase 18 (USP18), a DUB which also employs a nucleophilic cysteine and which selectively catalyzes protein deISG15ylations.^35^, ^45^ It has been proposed that the DUB activity of PL^pro^ modulates virulence via catalyzing the deISG15ylation of post-translationally ISG15ylated IRF3.^39^ However, to our knowledge, the DUB activity of isolated recombinant PL^pro^ has not yet been validated with oligopeptide substrates *in vitro*.

We developed SPE-MS assays that directly monitor the hydrolysis of the isopeptide amide bond of synthetic *N*^ε^-lysine-branched oligopeptides, which mimic post-translational protein modification by Ub/UBLs, catalyzed by isolated recombinant SARS-CoV-2 PL^pro^, extending the scope of our reported PL^pro^ SPE-MS assays with linear oligopeptides as substrates to *N*^ε^-lysine-branched oligopeptide substrates.^47^ The SPE-MS assays compare favorably to reported spectroscopic- or MS-based DUB assays, because these do not employ substrates that bind to the PL^pro^ S′ sites,^39^, ^40^, ^48^, ^49^ and are associated with comparatively high costs of the substrates.^51^ Due to variations in the catalytic efficiency of different PL^pro^ batches, we thus determined the rank order of substrate preference by quantifying product formation relative to internal standards.

Importantly, our results with *N*^ε^-lysine-branched oligopeptides support cellular studies concerning the DUB activities of PL^pro^, including its reported preference for catalyzing the hydrolysis of isopeptide amide bonds C-terminal to the LRGG motif of ISG15 rather than Ub/Ub_2_ and, in particular, NEDD8.^39^, ^40^ The rank order observed for PL^pro^-catalyzed hydrolysis of K_193_-branched IRF3-derived oligopeptides mimicking post-translational modification by UBLs was: IRF3_189-197_-ISG15 (**4**) > IRF3_189-197_-Ub (**15**) > IRF3_189-197_-NEDD8 (**16**) > IRF3_189-197_-URM1 (**17**) > IRF3_189-197_-SUMO1 (**18**), the latter of which was not a substrate of PL^pro^ (Fig. 3a). The results thus imply that the substrate preference of PL^pro^ not only depends on the fold of the substrate, interactions of the substrate at the PL^pro^ S1-S4 sites,^42^, ^65^ and on allosteric interactions with PL^pro^,^42^, ^43^, ^44^ but also on the sequence identity of the region of the substrate which binds to the active site beyond S1-S4. This proposal is further supported by the pronounced effect of amino acid substitutions in the *N*^ε^-lysine-branched oligopeptide substrates on PL^pro^ catalysis (Fig. 4), as well as by reported work on the hydrolysis of linear oligopeptides catalyzed by SARS-CoV-2 PL^pro^ and PL^pro^s of other coronaviruses.^80^, ^81^

The combined results show that the primary sequence of both the peptide fragment derived from the C-terminus of the UBL and that of the post-translationally modified human protein affects the efficiency of PL^pro^ catalysis, the latter potentially by binding to the S′ sites of PL^pro^, possibly in a substrate context-dependent manner. Hence, SPE-MS assays with oligopeptides have potential to predict the efficacy with which PL^pro^ catalyzes the hydrolysis of post-translationally modified (human) proteins, although factors other than the primary sequence also contribute to catalysis efficiency (see above). It will be useful to perform studies with the *N*^ε^-lysine-branched oligopeptides and isolated recombinant SARS-CoV PL^pro^ and MERS-CoV PL^pro^, as well as reported variants of SARS-CoV-2 PL^pro^, to inform on different substrate preferences and on how these differences manifest in altered virulence. Similarly, the substrate preferences of viral proteases other than coronavirus PL^pro^s, that have been reported to catalyze protein deISG15ylation and/or deubiquitinylation,^82^, ^83^, ^84^ should be examined.

The results indicate that PL^pro^ may catalyze the deISG15ylation of IRF3 regardless of the ISG15ylation site (Fig. 2c); studies with SARS-CoV-2 infected human cells are required to investigate the significance of this observation in a cellular context. Note that the ISG15ylation of IRF3 at the *N*^ε^-amino group of K193, K366, and, by implication, K360 may compete with other post-translational modifications, *e.g.* ubiquitinylation at K193,^54^ and/or acetylation^85^ and methylation^86^ at K366, in a manner potentially affecting PL^pro^ catalysis. SPE-MS assay results with *N*^ε^-lysine-branched oligopeptides mimicking the ISG15ylation of 4EHP, the ISG15ylation of which has been proposed to control translation during immune response,^59^ suggest that ISG15ylated 4EHP may be a substrate of SARS-CoV-2 PL^pro^ in cells (Fig. 3b). The relevance of the PL^pro^-catalyzed deISG15ylation of ISG15ylated 4EHP for virulence should thus also be probed in cellular studies.

It is likely that PL^pro^ not only catalyzes the deISG15ylation of IRF3 and 4EHP (Fig. 3), but also of other human proteins.^87^ Further substrate selectivity studies are, however, currently hampered by the lack of information on the exact ISG15ylation sites of human proteins.^35^, ^55^, ^56^, ^57^, ^58^ Note that evidence suggests that residues other than lysine residues may be ISG15ylated, *e.g.* cysteine residues^88^, ^89^; thus, the ability of SARS-CoV-2 PL^pro^ to catalyze the deISG15ylation of other residues than lysine should be subject of future work. Considering that protein ubiquitinylation is apparently more widespread than ISG15ylation, it is possible that human ubiquitinylated proteins may be identified which are more efficient substrates than ISG15ylated IRF3 *in vitro* and in cells.

Viral proteins can also be ISG15ylated by host proteins, in a manner which may impede their function, *e.g.* during replication.^90^, ^91^, ^92^ PL^pro^ may catalyze the deISG15ylation of ISG15ylated viral proteins to counteract the host innate immune response. Although, to our knowledge, little information is currently available on SARS-CoV-2 protein ISG15ylation, multiple ubiquitinylation sites have been identified in SARS-CoV-2 proteins in proteomic MS studies.^62^ Our work provides MS evidence that isolated recombinant PL^pro^ catalyzes the hydrolysis of the isopeptide amide bond of an *N*^ε^-lysine-branched oligopeptide based on a reported ubiquitinylation site of the SARS-CoV-2 N protein, *i.e.* SARS-CoV-2_N_335-341_-Ub **23** (Fig. 3c). It is thus possible that PL^pro^ catalyzes the deubiquitinylation of at least some of the (poly)ubiquitinylated lysine residues of viral proteins to *e.g.* regulate protein function or to prevent protein degradation via the host proteasome, as (poly)ubiquitinylation can be a signal for proteasomal degradation and the removal of the (poly)ubiquitin may hence stabilize the viral protein; conversely, PL^pro^ has potential to also catalyze the deISG15ylation of ISG15ylated viral proteins.

In general, it appears that viral proteases may have evolved to catalyze deISG15ylation reactions as a strategy to evade the host innate immune response, at least during early stages of infection.^92^, ^93^, ^94^ Interestingly, viral proteases catalyze deISG15ylation reactions at different sites*, e.g.* the leader protease of foot-and-mouth-disease virus (FMDV) is reported to catalyze the hydrolysis of ISG15 N-terminal to diglycine of the LRGG motif,^82^ opposed to SARS-CoV-2 PL^pro^ which catalyzes the hydrolysis of ISG15 C-terminal to diglycine of the LRGG motif. Viral proteases have also been reported to catalyze the direct hydrolysis of the main chain of interferon regulatory factors,^95^, ^96^, ^97^ including that of IRF3 by SARS-CoV-2 PL^pro^,^18^ further highlighting the importance of modulating (post-translationally modified) interferon regulatory factors such as IRF3 during viral infections.

At least in most cases, the identity of the substrate residues binding to the S sites affects PL^pro^ catalysis to a relatively greater extent than the identity of the substrate residues binding to the S′ sites (Fig. 2). This observation may reflect the different substrate selectivities of PL^pro^ and M^pro^. M^pro^ apparently requires the presence of specific residues (*i.e.*, S/A/N) at the S1′ site for efficient catalysis, though its substrate requirements for S2′-S4′ appear to be less stringent.^98^ Defining the full substrate scope of M^pro^ is, however, the subject of ongoing investigations.^99^, ^100^ Notably, NEMO, the M^pro^-catalyzed hydrolysis of which has been reported to induce the death of brain endothelial cells,^16^, ^17^ has a valine residue at the P1′ position, indicating that the presence of (L/F/V)Q(S/A/N) motifs in human proteins may not be sufficient to predict the efficiency of M^pro^ catalysis.

Eleven sites in pp1a/1ab fulfil the apparently somewhat narrower substrate requirements for M^pro^, whereas only three sites in pp1a/1ab fulfil the substrate requirements for the apparently more promiscuous PL^pro^.^1^, ^2^ Human proteases that accept (L/F/V)Q(S/A/N) motifs as substrates are currently unknown, suggesting that M^pro^ may have evolved to exclusively catalyze the release of those nsps from pp1a/1ab that are directly involved in replication and transcription (*e.g.* nsp12, which has a RNA-dependent RNA polymerase domain, and the nsp13 helicase), under strict spatiotemporal control. By contrast, in principle, human DUBs could catalyze the hydrolysis of pp1a/1ab at three sites, releasing *inter alia* nsp1 and nsp2 which have been proposed to help the virus evade the host immune system,^101^ and thus compete with PL^pro^ for viral substrates. The apparent lower substrate specificity of PL^pro^ may reflect its functions in enabling the virus to counteract the host immune system, with respect to catalyzing both the release of nsp1 and nsp2 and the deISG15ylation of post-translationally modified human and, potentially, viral proteins, a process which might not require strict spatiotemporal control and thus tolerates reduced substrate specificity.

The ability of PL^pro^, but not M^pro^ (as far as is known), to catalyze hydrolysis of isopeptide as well as peptide amide bonds is striking. The comparison of substrate and non-substrate binding modes for the two proteases is thus of interest, because the precise nature of the dynamic interactions that regulate the different substrate selectivities of PL^pro^ and M^pro^ is not defined. Reported PL^pro^ structures reveal that substrate binding can induce conformational changes of flexible regions proximate to the active site, including the PL^pro^ blocking loop 2 (BL2) which is a key factor in regulating the accessibility of potential substrates to the active site.^70^ The role of the PL^pro^ BL2 in regulating substrate recognition is precedented by BL2 in, at least some, human DUBs, including *e.g.* USP7 and USP14;^71^, ^72^ however, in human DUBs, post-translational modification of BL2 residues may further alter catalysis.^102^ It appears that the PL^pro^ substrate selectivity is not only a result of the affinity of the substrate to bind to S sites and, to a lesser extent, S′ sites, but also of the ability of a potential substrate to stabilize the closed conformation of BL2, so enabling a catalytically productive enzyme-substrate conformation which may be achieved via interactions with PL^pro^ that may be relatively remote from the active site, including by specific interactions of hydrophobic substrate residues with the S′ sites (Fig. 6).

Movement of active site bordering loops to bind, enclose, and/or orientate substrates (and reject non-substrates) are common in enzyme catalysis. For example, substrate binding can induce conformational changes in flexible regions of M^pro^ that directly contribute to shaping the S sites, in particular of an α-helix (T45-L50), which contributes to forming the S2 site, and of a loop (D187-Q192), which contributes to forming the S4 site.^98^, ^103^, ^104^, ^105^, ^106^ The presence of a BL2-type loop in PL^pro^, but not M^pro^, is interesting. Whether or not the presence of this loop affects the ability of PL^pro^ to accept different types of substrates (peptide and isopeptide amide bonds), whilst manifesting an apparently narrower selectivity with respect to polyprotein pp1a/1ab hydrolysis, is unclear. In this regard, it will be of interest to investigate how the substrate selectivities of PL^pro^ homologues have changed during the course of viral evolution, both with respect to pp1a/1ab and proteins in different host organisms, *e.g.* SARS-CoV-2 PL^pro^ prefers ISG15 as a substrate, whereas SARS-CoV PL^pro^ prefers diubiquitin.^39^, ^40^ Notably, the efficiency and selectivity of MERS-CoV PL^pro^ catalysis has been proposed to be distinct from that of SARS-CoV and SARS-CoV-2 PL^pro^, in part as a result of amino acid variations in the BL2 loop.^48^, ^74^

SPE-MS assays have been used to investigate post-translational protein modifications and catalysis of nucleophilic cysteine enzymes, including isolated recombinant SARS-CoV-2 PL^pro^ and M^pro^.^10^, ^47^, ^49^, ^107^, ^108^, ^109^, ^110^, ^111^, ^112^, ^113^ Our SPE-MS assays employing synthetic *N*^ε^-lysine-branched oligopeptides as substrates of isolated recombinant PL^pro^ have enabled studies on the substrate scope and substrate selectivity of PL^pro^ and thus helped to characterize the DUB activity of PL^pro^. The combination of analogous MS-based assays with synthetic *N*^ε^-lysine-branched oligopeptides as substrates of isolated recombinant human DUBs, together with proteomic studies, will enable studies on the substrate preferences of human DUBs, such as USP18 which also catalyzes protein deISG15ylation, and which is a current medicinal chemistry target.^35^, ^45^

## 4 Experimental section

### 4.1 Production and purification of isolated recombinant SARS-CoV-2 PL^pro^

The PL^pro^ domain of the SARS-CoV-2 nsp3 (E746-T1063) was produced using *E. coli* Lemo21(DE3) cells and purified as reported previously.^47^

### 4.2 Peptide synthesis

Linear oligopeptides were prepared by solid phase peptide synthesis (SPPS) using a Liberty Blue peptide synthesizer (CEM Microwave Technology Ltd.), as reported for the synthesis of the oligopeptides **1**–**3**.^47^

The *N*^ε^-lysine-branched oligopeptides mimicking post-translationally modified proteins were synthesized by microwave-assisted SPPS using the Fmoc-protection strategy from the C- to N-terminus on Rink Amide MBHA resin (AGTC Bioproducts Ltd.; loading: 0.6–0.8 mmol/g) similar to reported procedures.^47^ Initially, a linear oligopeptide was synthesized; *N*-Fmoc lysine with a 4-methyltrityl (Mtt) protected *N*^ε^-amine was used at the site of the lysine branching, and an amino acid with a Boc-protected *N*^α^-amine was used at the N-terminus. Branching was introduced via selective deprotection of the lysine *N*^ε^-Mtt group of the resin-bound peptides using 1%_v/v_ trifluoroacetic acid (TFA) and 2%_v/v_ triisopropylsilane (TIPS) in dichloromethane at ambient temperature while shaking (300 rpm) (5 cycles; after each cycle, the deprotection mixture was removed and the resin was washed with dichloromethane). Following Mtt deprotection, the UBL-derived peptide fragment was synthesized by SPPS from the C- to N-terminus starting with the free *N*^ε^-amino group of the resin-bound peptides at the C-terminus.

After completion of the synthesis, the resin-bound peptides were washed with dichloromethane and subsequently cleaved from the resin and simultaneously deprotected using a mixture of trifluoroacetic acid, triisopropylsilane, 1,3-dimethoxybenzene, and water (92.5/2.5/2.5/2.5%_v/v_, respectively). Solids were separated; the remaining clear solution was diluted with diethyl ether (45 mL/0.1 mmol resin). After incubation for 30 min at 0 °C, the mixture was centrifuged for 10 min using a Beckman Coulter Allegra X-30R centrifuge equipped with a SX4400 rotor (4500 rpm); the supernatant was discarded. The solid residue was dissolved in a water/acetonitrile mixture, frozen using liquid N_2_, and then lyophilized. The dried crude product was dissolved in a water/acetonitrile mixture, filtered, and purified using a semi-preparative HPLC machine (Shimadzu UK Ltd.) equipped with a reverse phase column (Gemini 00G-4454-U0-AX; phase: NX-C18). A linear gradient (typically 2–47%_v/v_ over 38 min) of acetonitrile in milli-Q grade water (each containing 0.1%_v/v_ trifluoroacetic acid) was used as eluent. Fractions were analyzed by SPE-MS and those containing the pure peptide were combined and lyophilized. Sequences, mass spectra, and purification characteristics of the synthetic oligopeptides are shown in Supporting Figure S1.

### 4.3 SPE-MS assays

PL^pro^ assays for turnover and competition experiments were performed in 96-well polypropylene assay plates (Greiner), either with a 1.0 or 0.5 mL final reaction volume, using isolated recombinant SARS-CoV-2 PL^pro^ (0.2 μM), substrate peptide(s) (2.0 μM), inert *N*-acetylated standard peptides (0.2 μM, as indicated in the individual experiments) in buffer (50 mM Tris, pH 8.0) at ambient temperature. PL^pro^ catalysis was directly monitored using SPE-MS.^47^ The RapidFire RF 365 high-throughput sampling robot used was programmed to aspirate samples from the reaction mixture at the time intervals indicated in the individual experiments.

MS-analyses were performed using a RapidFire RF 365 high-throughput sampling robot (Agilent) attached to an iFunnel Agilent 6550 accurate mass quadrupole time-of-flight (Q-TOF) mass spectrometer operated in the positive ionization mode.^47^ Assay samples were aspirated under vacuum for 0.6 s and loaded onto a C4 solid phase extraction (SPE) cartridge. After loading, the C4 SPE cartridge was washed with 0.1%_v/v_ aqueous formic acid to remove non-volatile buffer salts (5.5 s, 1.5 mL/min). The peptide was eluted from the SPE cartridge with 0.1%_v/v_ aqueous formic acid in 85/15_v/v_ acetonitrile/water into the mass spectrometer (5.5 s, 1.25 mL/min) and the SPE cartridge re-equilibrated with 0.1%_v/v_ aqueous formic acid (0.5 s, 1.25 mL/min). The mass spectrometer parameters were: capillary voltage: 4000 V; nozzle voltage: 1000 V; fragmentor voltage: 365 V; gas temperature: 280 °C; gas flow: 13 L/min; sheath gas temperature: 350 °C; sheath gas flow: 12 L/min.

For data analysis and to quantify product formation, the charge states of both the C-terminal and N-terminal product peptides and the corresponding *N*-acetylated C-terminal and N-terminal product peptides (*i.e.* **5**, **12**, and **24**), which were used as internal standards, were used to extract ion chromatogram data (*m*/*z* +1 for **12** and the corresponding product peptide; *m*/*z* +2 for **5** and **24**, as well as for the corresponding product peptides); peak areas were integrated using the RapidFire Integrator software (Agilent). Data were exported into Microsoft Excel and used to calculate the product peptide concentrations using the equation: peptide concentration = 0.2 μM × (integral C- or N-terminal product peptide) / (integral *N*-acetylated C- or N-terminal product peptide).

### 4.4 Preparation of PL^pro^:oligopeptide models

A reported SARS-CoV-2 PL^pro^ structure (PDB ID: 6WX4^65^) was prepared for modelling as described (Supporting Table S2).^61^ Based on reported quantum mechanics/molecular mechanics-umbrella sampling (QM/MM-US) calculations on proton transfer processes in the PL^pro^ catalytic triad,^61^ C111 was modelled in its deprotonated form, H272 as doubly protonated, and D286 as deprotonated.

The *N*^ε^-lysine-branched oligopeptides complexed with PL^pro^ were constructed using reported AutoDock CrankPep (ADCP)^66^-docked conformations of the linear oligopeptide nsp2/3_808-827_ (**2**).^61^ The residues N-terminal to the scissile amide in the top ranked pose (d2_01, reported nomenclature^61^), which was successful in placing P4-P1 LKGG in the respective S4-S1 subsites, were used to build the N-terminal ubiquitin (Ub) or Ub-like modifier (UBL) derived fragment of the *N*^ε^-lysine-branched oligopeptides. The C-terminal fragment of the *N*^ε^-lysine-branched oligopeptides was built based on the 48th-ranked pose of **2** (d2_48), which did not pass through S4-S1, but in which the P4′ lysine sidechain *N*^ε^ amine was proximate to C111 (Supporting Figure S5). The lysine side chain branching was modelled by linking the C^ε^ atom of the d2_48 P4′ lysine sidechain to the carboxamide N atom of the 2_01 P1′ alanine. To prepare each of the eight branched peptides in IRF3_189-197_-(ISG15/Ub/NEDD8/URM1/SUMO1) and (TMEM59_285-291_/TMEM59_298-305_/SARS-CoV-2_N_335-341_)-Ub (Fig. 3), the two poses of **2** were modified to the lengths of the N- and C-terminal fragments. The residues were modified to the target sequences using the mutagenesis tool of PyMOL (open source, v. 2.3.0),^114^ selecting the least sterically clashing backbone-dependent rotamer in each case.^115^ The N-termini of both fragments were uncapped, while the C-terminus of the IRF3_189-197_/TMEM59_285-291_/TMEM59_298-305_/SARS-CoV-2_N_335-341_ fragment was NH_2_-capped. Peptide histidine residues were modelled in their neutral state, with *N*^ε^ protonated (HIE in AMBER nomenclature),^116^ except for IRF3_189-197_-URM1 where both the neutral HIE and the positively charged, doubly protonated (HIP) states of the P3 histidine residue were considered, denoted as URM1^N^ and URM1^+^ respectively.

### 4.5 Parametrization and molecular dynamics (MD)

MD simulations of the PL^pro^:oligopeptide complexes were performed in GROMACS (v. 2019.2, 2020.4),^117^ using the AMBERFF99SB-ILDN/TIP3P forcefield.^116^, ^118^ Non-bonded Zn parameters were employed.^119^ For the non-standard *N*^ε^-branched lysine residue, *N*-butyl acetamide (Supporting Figure S32) was used as a model for parametrization with AMBER atom types and RESP charges (Gaussian16 and antechamber in Amber18).^120^, ^121^, ^122^, ^123^, ^124^ The new residue, designated as “LYC”, was incorporated into GROMACS (Supporting Figure S33), with its *N*^ε^ atom bonded to the P1 Gly C atom. Amide improper dihedral parameters centered at the GLY_C and LYC_NZ atoms were included to maintain amide group planarity.

Each of the nine PL^pro^:oligopeptide complexes (eight unique sequences, with URM1^N^ and URM1^+^ for two possible charge states of the P3 histidine group in **17**) was centered in a rhombic dodecahedral box with at least 1.0 nm separation from box edges, solvated, neutralized with sodium/chloride ions (110,788–110,847 atoms in total), and minimized until the maximum force was below 1000 kJ mol^−1^ nm^−1^. From the minimized system, three replicas were initiated using random velocities at 298.15 K, subjected to 200 ps (1 fs step) restrained NVT equilibration at 298.15 K, followed by 200 ps (1 fs step) NPT equilibration at 298.15 K and 1.0 bar. The equilibrated complexes were subjected to 200 ns production MD (2 fs step). A velocity-rescaling thermostat with a stochastic term (time constant 0.1 ps; protein and non-protein coupled separately)^125^ and a Parrinello-Rahman barostat (time constant 2 ps) were used.^126^, ^127^ Long-range electrostatic interactions were calculated by smooth Particle-mesh Ewald (1 nm cut-off).^128^, ^129^ Van der Waals interactions were cut off at 1 nm.

The 3 × 200 ns MD trajectories were fitted based on the PL^pro^ backbone and analyzed using GROMACS tools (v 2019.2).^117^ A hydrogen bond was defined on observation of a donor–acceptor distance <3.5 Å and hydrogen-donor–acceptor angle <30°. To obtain representative structures, clustering was performed with a 3 Å RMSD cut-off of the peptide backbone, using the gromos algorithm.^130^ Per residue decomposition of PL^pro^:oligopeptide binding energies calculated by molecular mechanics/generalized Born surface area (MM/GBSA)^131^, ^132^, ^133^, ^134^ was performed on frames in 5 ns intervals, using MMPBSA.py (AMBER18) with an ionic strength of 0.15 M, mbondi2 radii, and *igb* = 5.^124^, ^135^, ^136^

## Declaration of Competing Interest

The authors declare that they have no known competing financial interests or personal relationships that could have appeared to influence the work reported in this paper.

## Data Availability

Data will be made available on request.

## References

[1] V’kovski P., Kratzel A., Steiner S., Stalder H., Thiel V. (2021). Coronavirus biology and replication: implications for SARS-CoV-2. Nat Rev Microbiol.

[2] Thiel V., Ivanov K.A., Putics Á. (2003). Mechanisms and enzymes involved in SARS coronavirus genome expression. J Gen Virol.

[3] Li G., Hilgenfeld R., Whitley R., De Clercq E. (2023). Therapeutic strategies for COVID-19: progress and lessons learned. Nat Rev Drug Discov.

[4] Cannalire R., Cerchia C., Beccari A.R., Di Leva F.S., Summa V. (2022). Targeting SARS-CoV-2 proteases and polymerase for COVID-19 treatment: state of the art and future opportunities. J Med Chem.

[5] Ghosh A.K., Mishevich J.L., Mesecar A., Mitsuya H. (2022). Recent drug development and medicinal chemistry approaches for the treatment of SARS-CoV-2 infection and COVID-19. ChemMedChem.

[6] Lv Z., Cano K.E., Jia L., Drag M., Huang T.T., Olsen S.K. (2022). Targeting SARS-CoV-2 proteases for COVID-19 antiviral development. Front Chem.

[7] Ton A.-T., Pandey M., Smith J.R., Ban F., Fernandez M., Cherkasov A. (2022). Targeting SARS-CoV-2 papain-like protease in the postvaccine era. Trends Pharmacol Sci.

[8] Tan H., Hu Y., Jadhav P., Tan B., Wang J. (2022). Progress and challenges in targeting the SARS-CoV-2 papain-like protease. J Med Chem.

[9] Owen D.R., Allerton C.M.N., Anderson A.S. (2021). An oral SARS-CoV-2 Mpro inhibitor clinical candidate for the treatment of COVID-19. Science.

[10] Unoh Y., Uehara S., Nakahara K. (2022). Discovery of S-217622, a noncovalent oral SARS-CoV-2 3CL protease inhibitor clinical candidate for treating COVID-19. J Med Chem.

[11] Mielech A.M., Kilianski A., Baez-Santos Y.M., Mesecar A.D., Baker S.C. (2014). MERS-CoV papain-like protease has deISGylating and deubiquitinating activities. Virology.

[12] Yang X., Chen X., Bian G. (2014). Proteolytic processing, deubiquitinase and interferon antagonist activities of middle east respiratory syndrome coronavirus papain-like protease. J Gen Virol.

[13] Lindner H.A., Lytvyn V., Qi H., Lachance P., Ziomek E., Ménard R. (2007). Selectivity in ISG15 and ubiquitin recognition by the SARS coronavirus papain-like protease. Arch Biochem Biophys.

[14] Ratia K., Kilianski A., Baez-Santos Y.M., Baker S.C., Mesecar A. (2014). Structural basis for the ubiquitin-linkage specificity and deISGylating activity of SARS-CoV papain-like protease. PLoS Pathog.

[15] Chen Z., Wang Y., Ratia K., Mesecar A.D., Wilkinson K.D., Baker S.C. (2007). Proteolytic processing and deubiquitinating activity of papain-like proteases of human coronavirus NL63. J Virol.

[16] Wenzel J., Lampe J., Müller-Fielitz H. (2021). The SARS-CoV-2 main protease Mpro causes microvascular brain pathology by cleaving NEMO in brain endothelial cells. Nat Neurosci.

[17] Hameedi M.A., Prates E.T., Garvin M.R. (2022). Structural and functional characterization of NEMO cleavage by SARS-CoV-2 3CLpro. Nat Commun.

[18] Moustaqil M., Ollivier E., Chiu H.-P. (2021). SARS-CoV-2 proteases PLpro and 3CLpro cleave IRF3 and critical modulators of inflammatory pathways (NLRP12 and TAB1): implications for disease presentation across species. Emerg Microbes Infect.

[19] Reynolds N.D., Aceves N.M., Liu J.L. (2021). The SARS-CoV-2 SSHHPS recognized by the papain-like protease. ACS Infect Dis.

[20] Mohamud Y., Xue Y.C., Liu H. (2021). The papain-like protease of coronaviruses cleaves ULK1 to disrupt host autophagy. Biochem Biophys Res Commun.

[21] Meyer B., Chiaravalli J., Gellenoncourt S. (2021). Characterising proteolysis during SARS-CoV-2 infection identifies viral cleavage sites and cellular targets with therapeutic potential. Nat Commun.

[22] Haas A.L., Ahrens P., Bright P.M., Ankel H. (1987). Interferon induces a 15-kilodalton protein exhibiting marked homology to ubiquitin. J Biol Chem.

[23] Loeb K.R., Haas A.L. (1992). The interferon-inducible 15-kDa ubiquitin homolog conjugates to intracellular proteins. J Biol Chem.

[24] Narasimhan J., Wang M., Fu Z., Klein J.M., Haas A.L., Kim J.-J.-P. (2005). Crystal structure of the interferon-induced ubiquitin-like protein ISG15. J Biol Chem.

[25] Kang J.A., Kim Y.J., Jeon Y.J. (2022). The diverse repertoire of ISG15: more intricate than initially thought. Exp Mol Med.

[26] Kumar S., Tomooka Y., Noda M. (1992). Identification of a set of genes with developmentally down-regulated expression in the mouse brain. Biochem Biophys Res Commun.

[27] Kamitani T., Kito K., Nguyen H.P., Yeh E.T.H. (1997). Characterization of NEDD8, a developmentally down-regulated ubiquitin-like protein. J Biol Chem.

[28] Furukawa K., Mizushima N., Noda T., Ohsumi Y. (2000). A protein conjugation system in yeast with homology to biosynthetic enzyme reaction of prokaryotes. J Biol Chem.

[29] Van der Veen A.G., Schorpp K., Schlieker C. (2011). Role of the ubiquitin-like protein Urm1 as a noncanonical lysine-directed protein modifier. Proc Natl Acad Sci USA.

[30] van der Veen A.G., Ploegh H.L. (2012). Ubiquitin-like proteins. Ann Rev Biochem.

[31] Hochstrasser M. (2009). Origin and function of ubiquitin-like proteins. Nature.

[32] Dikic I., Schulman B.A. (2023). An expanded lexicon for the ubiquitin code. Nat Rev Mol Cell Biol.

[33] Glickman M.H., Ciechanover A. (2002). The ubiquitin-proteasome proteolytic pathway: destruction for the sake of construction. Physiol Rev.

[34] Lecker S.H., Goldberg A.L., Mitch W.E. (2006). Protein degradation by the ubiquitin–proteasome pathway in normal and disease states. J Am Soc Nephrol.

[35] Jiménez Fernández D., Hess S., Knobeloch K.-P. (2020). Strategies to target ISG15 and USP18 toward therapeutic applications. Front Chem.

[36] Cappadocia L., Lima C.D. (2018). Ubiquitin-like protein conjugation: structures, chemistry, and mechanism. Chem Rev.

[37] Lange S.M., Armstrong L.A., Kulathu Y. (2022). Deubiquitinases: from mechanisms to their inhibition by small molecules. Mol Cell.

[38] Komander D., Clague M.J., Urbé S. (2009). Breaking the chains: structure and function of the deubiquitinases. Nat Rev Mol Cell Biol.

[39] Shin D., Mukherjee R., Grewe D. (2020). Papain-like protease regulates SARS-CoV-2 viral spread and innate immunity. Nature.

[40] Klemm T., Ebert G., Calleja D.J. (2020). Mechanism and inhibition of the papain-like protease, PLpro, of SARS-CoV-2. EMBO J.

[41] Lei J., Kusov Y., Hilgenfeld R. (2018). Nsp3 of coronaviruses: structures and functions of a large multi-domain protein. Antiviral Res.

[42] Patchett S., Lv Z., Rut W. (2021). A molecular sensor determines the ubiquitin substrate specificity of SARS-CoV-2 papain-like protease. Cell Rep.

[43] van Vliet V.J.E., Huynh N., Palà J. (2022). Ubiquitin variants potently inhibit SARS-CoV-2 PLpro and viral replication via a novel site distal to the protease active site. PLoS Pathog.

[44] Wydorski P.M., Osipiuk J., Lanham B.T. (2023). Dual domain recognition determines SARS-CoV-2 PLpro selectivity for human ISG15 and K48-linked di-ubiquitin. Nat Commun.

[45] Malakhov M.P., Malakhova O.A., Kim K.I., Ritchie K.J., Zhang D.-E. (2002). UBP43 (USP18) specifically removes ISG15 from conjugated proteins. J Biol Chem.

[46] Gold I.M., Reis N., Glaser F., Glickman M.H. (2022). Coronaviral PLpro proteases and the immunomodulatory roles of conjugated versus free Interferon Stimulated Gene product-15 (ISG15). Semin Cell Dev Biol.

[47] Brewitz L., Kamps J.J.A.G., Lukacik P. (2022). Mass spectrometric assays reveal discrepancies in inhibition profiles for the SARS-CoV-2 papain-like protease. ChemMedChem.

[48] Freitas B.T., Durie I.A., Murray J. (2020). Characterization and noncovalent inhibition of the deubiquitinase and deISGylase activity of SARS-CoV-2 papain-like protease. ACS Infect Dis.

[49] Redhead M.A., Owen C.D., Brewitz L. (2021). Bispecific repurposed medicines targeting the viral and immunological arms of COVID-19. Sci Rep.

[50] Ritorto M.S., Ewan R., Perez-Oliva A.B. (2014). Screening of DUB activity and specificity by MALDI-TOF mass spectrometry. Nat Commun.

[51] Armstrong L.A., Lange S.M., Dee Cesare V. (2021). Biochemical characterization of protease activity of Nsp3 from SARS-CoV-2 and its inhibition by nanobodies. PLoS One.

[52] Wu F., Zhao S., Yu B. (2020). A new coronavirus associated with human respiratory disease in China. Nature.

[53] Shi H.-X., Yang K., Liu X. (2010). Positive regulation of interferon regulatory factor 3 activation by Herc5 via ISG15 modification. Mol Cell Biol.

[54] Chattopadhyay S., Kuzmanovic T., Zhang Y., Wetzel J.L., Sen G.C. (2016). Ubiquitination of the transcription factor IRF-3 activates RIPA, the apoptotic pathway that protects mice from viral pathogenesis. Immunity.

[55] Giannakopoulos N.V., Luo J.-K., Papov V. (2005). Proteomic identification of proteins conjugated to ISG15 in mouse and human cells. Biochem Biophys Res Commun.

[56] Li C., Nelson T.G., Vertegaal A.C.O., Thibault P. (2021). Proteomic strategies for characterizing ubiquitin-like modifications. Nat Rev Methods Primers.

[57] Zhao C., Denison C., Huibregtse J.M., Gygi S., Krug R.M. (2005). Human ISG15 conjugation targets both IFN-induced and constitutively expressed proteins functioning in diverse cellular pathways. Proc Natl Acad Sci USA.

[58] Zhang Y., Thery F., Wu N.C. (2019). The in vivo ISGylome links ISG15 to metabolic pathways and autophagy upon Listeria monocytogenes infection. Nat Commun.

[59] Okumura F., Zou W., Zhang D.-E. (2007). ISG15 modification of the eIF4E cognate 4EHP enhances cap structure-binding activity of 4EHP. Genes Dev.

[60] Glaser W., Skern T. (2000). Extremely efficient cleavage of eIF4G by picornaviral proteinases L and 2A in vitro. FEBS Lett.

[61] Chan H.T.H., Brewitz L., Lukacik P. (2023). Studies on the selectivity of the SARS-CoV-2 papain-like protease reveal the importance of the P2′ proline of the viral polyprotein. bioRxiv.

[62] Stukalov A., Girault V., Grass V. (2021). Multilevel proteomics reveals host perturbations by SARS-CoV-2 and SARS-CoV. Nature.

[63] Kuo C.-J., Chao T.-L., Kao H.-C. (2021). Kinetic characterization and inhibitor screening for the proteases leading to identification of drugs against SARS-CoV-2. Antimicrob Agents Chemother.

[64] Nakashima Y., Brewitz L., Tumber A., Salah E., Schofield C.J. (2021). 2-Oxoglutarate derivatives can selectively enhance or inhibit the activity of human oxygenases. Nat Commun.

[65] Rut W., Lv Z., Zmudzinski M. (2020). Activity profiling and crystal structures of inhibitor-bound SARS-CoV-2 papain-like protease: A framework for anti–COVID-19 drug design. Sci Adv.

[66] Zhang Y., Sanner M.F. (2019). AutoDock CrankPep: combining folding and docking to predict protein–peptide complexes. Bioinformatics.

[67] Báez-Santos Y.M., St. John S.E., Mesecar A.D. (2015). The SARS-coronavirus papain-like protease: structure, function and inhibition by designed antiviral compounds. Antivir Res..

[68] Henderson J.A., Verma N., Harris R.C., Liu R., Shen J. (2020). Assessment of proton-coupled conformational dynamics of SARS and MERS coronavirus papain-like proteases: Implication for designing broad-spectrum antiviral inhibitors. J Chem Phys.

[69] Gao X., Qin B., Chen P. (2021). Crystal structure of SARS-CoV-2 papain-like protease. Acta Pharm Sin B.

[70] Fu Z., Huang B., Tang J. (2021). The complex structure of GRL0617 and SARS-CoV-2 PLpro reveals a hot spot for antiviral drug discovery. Nat Commun.

[71] Hu M., Li P., Li M. (2002). Crystal structure of a UBP-family deubiquitinating enzyme in isolation and in complex with ubiquitin aldehyde. Cell.

[72] Hu M., Li P., Song L. (2005). Structure and mechanisms of the proteasome-associated deubiquitinating enzyme USP14. EMBO J.

[73] Ratia K., Pegan S., Takayama J. (2008). A noncovalent class of papain-like protease/deubiquitinase inhibitors blocks SARS virus replication. Proc Natl Acad Sci USA.

[74] Lee H., Lei H., Santarsiero B.D. (2015). Inhibitor recognition specificity of MERS-CoV papain-like protease may differ from that of SARS-CoV. ACS Chem Biol.

[75] Osipiuk J., Azizi S.-A., Dvorkin S. (2021). Structure of papain-like protease from SARS-CoV-2 and its complexes with non-covalent inhibitors. Nat Commun.

[76] Shen Z., Ratia K., Cooper L. (2022). Design of SARS-CoV-2 PLpro inhibitors for COVID-19 antiviral therapy leveraging binding cooperativity. J Med Chem.

[77] Wertz I.E., Murray J.M. (2019). Structurally-defined deubiquitinase inhibitors provide opportunities to investigate disease mechanisms. Drug Discov Today Technol.

[78] Zhao Y., Du X., Duan Y. (2021). High-throughput screening identifies established drugs as SARS-CoV-2 PLpro inhibitors. Protein Cell.

[79] Ma C., Sacco M.D., Xia Z. (2021). Discovery of SARS-CoV-2 Papain-like Protease Inhibitors through a Combination of High-Throughput Screening and a FlipGFP-Based Reporter Assay. ACS Cent Sci.

[80] Drag M., Mikolajczyk J., Bekes M. (2008). Positional-scanning fluorigenic substrate libraries reveal unexpected specificity determinants of DUBs (deubiquitinating enzymes). Biochem J.

[81] Rut W., Zmudzinski M., Snipas S.J., Bekes M., Huang T.T., Drag M. (2020). Engineered unnatural ubiquitin for optimal detection of deubiquitinating enzymes. Chem Sci.

[82] Swatek K.N., Aumayr M., Pruneda J.N. (2018). Irreversible inactivation of ISG15 by a viral leader protease enables alternative infection detection strategies. Proc Natl Acad Sci USA.

[83] Akutsu M., Ye Y., Virdee S., Chin J.W., Komander D. (2011). Molecular basis for ubiquitin and ISG15 cross-reactivity in viral ovarian tumor domains. Proc Natl Acad Sci USA.

[84] James T.W., Frias-Staheli N., Bacik J.-P. (2011). Structural basis for the removal of ubiquitin and interferon-stimulated gene 15 by a viral ovarian tumor domain-containing protease. Proc Natl Acad Sci USA.

[85] Huai W., Liu X., Wang C. (2019). KAT8 selectively inhibits antiviral immunity by acetylating IRF3. J Exp Med.

[86] Wang C., Wang Q., Xu X. (2017). The methyltransferase NSD3 promotes antiviral innate immunity via direct lysine methylation of IRF3. J Exp Med.

[87] Munnur D., Teo Q., Eggermont D. (2021). Altered ISGylation drives aberrant macrophage-dependent immune responses during SARS-CoV-2 infection. Nat Immunol.

[88] Bade V.N., Nickels J., Keusekotten K., Praefcke G.J.K. (2012). Covalent protein modification with ISG15 via a conserved cysteine in the hinge region. PLoS One.

[89] Jeon S.J., Chung K.C. (2022). Covalent conjugation of ubiquitin-like ISG15 to apoptosis-inducing factor exacerbates toxic stimuli-induced apoptotic cell death. J Biol Chem.

[90] Durfee L.A., Lyon N., Seo K., Huibregtse J.M. (2010). The ISG15 conjugation system broadly targets newly synthesized proteins: implications for the antiviral function of ISG15. Mol Cell.

[91] Zhao C., Hsiang T.-Y., Kuo R.-L., Krug R.M. (2010). ISG15 conjugation system targets the viral NS1 protein in influenza A virus–infected cells. Proc Natl Acad Sci USA.

[92] Perng Y.-C., Lenschow D.J. (2018). ISG15 in antiviral immunity and beyond. Nat Rev Microbiol.

[93] Frias-Staheli N., Giannakopoulos N.V., Kikkert M. (2007). Ovarian tumor domain-containing viral proteases evade ubiquitin- and ISG15-dependent innate immune responses. Cell Host Microbe.

[94] Isaacson M.K., Ploegh H.L. (2009). Ubiquitination, ubiquitin-like modifiers, and deubiquitination in viral infection. Cell Host Microbe.

[95] Hung H.-C., Wang H.-C., Shih S.-R., Teng I.F., Tseng C.-P., Hsu J.T.A. (2011). Synergistic inhibition of enterovirus 71 replication by interferon and rupintrivir. J Infect Dis.

[96] Xiang Z., Liu L., Lei X., Zhou Z., He B., Wang J. (2016). 3C protease of enterovirus D68 inhibits cellular defense mediated by interferon regulatory factor 7. J Virol.

[97] Lei X., Xiao X., Xue Q., Jin Q., He B., Wang J. (2013). Cleavage of interferon regulatory factor 7 by enterovirus 71 3C suppresses cellular responses. J Virol.

[98] Zhao Y., Zhu Y., Liu X. (2022). Structural basis for replicase polyprotein cleavage and substrate specificity of main protease from SARS-CoV-2. Proc Natl Acad Sci USA.

[99] Pablos I., Machado Y., de Jesus H.C.R. (2021). Mechanistic insights into COVID-19 by global analysis of the SARS-CoV-2 3CLpro substrate degradome. Cell Rep.

[100] Koudelka T., Boger J., Henkel A. (2021). N-Terminomics for the identification of in vitro substrates and cleavage site specificity of the SARS-CoV-2 main protease. Proteomics.

[101] Yan W., Zheng Y., Zeng X., He B., Cheng W. (2022). Structural biology of SARS-CoV-2: open the door for novel therapies. Sig Transduct Target Ther.

[102] Xu D., Shan B., Lee B.-H. (2015). Phosphorylation and activation of ubiquitin-specific protease-14 by Akt regulates the ubiquitin-proteasome system. Elife.

[103] Kneller D.W., Phillips G., O’Neill H.M. (2020). Structural plasticity of SARS-CoV-2 3CL Mpro active site cavity revealed by room temperature X-ray crystallography. Nat Commun.

[104] Lee J., Kenward C., Worrall L.J. (2022). X-ray crystallographic characterization of the SARS-CoV-2 main protease polyprotein cleavage sites essential for viral processing and maturation. Nat Commun.

[105] Chan H.T.H., Moesser M.A., Walters R.K. (2021). Discovery of SARS-CoV-2 Mpro peptide inhibitors from modelling substrate and ligand binding. Chem Sci.

[106] Chan H.T.H., Oliveira A.S.F., Schofield C.J., Mulholland A.J., Duarte F. (2023). Dynamical nonequilibrium molecular dynamics simulations Identify allosteric sites and positions associated with drug resistance in the SARS-CoV-2 main protease. JACS Au.

[107] Malla T.R., Tumber A., John T. (2021). Mass spectrometry reveals potential of β-lactams as SARS-CoV-2 Mpro inhibitors. Chem Commun.

[108] Brewitz L., Dumjahn L., Zhao Y. (2023). Alkyne derivatives of SARS-CoV-2 main protease inhibitors including nirmatrelvir inhibit by reacting covalently with the nucleophilic cysteine. J Med Chem.

[109] Malla T.R., Brewitz L., Muntean D.-G. (2022). Penicillin derivatives inhibit the SARS-CoV-2 main protease by reaction with its nucleophilic cysteine. J Med Chem.

[110] Thun-Hohenstein S.T.D., Suits T.F., Malla T.R. (2022). Structure-activity studies reveal scope for optimisation of ebselen-type inhibition of SARS-CoV-2 main protease. ChemMedChem.

[111] Miura T., Malla T.R., Owen C.D. (2023). In vitro selection of macrocyclic peptide inhibitors containing cyclic γ2,4-amino acids targeting the SARS-CoV-2 main protease. Nat Chem.

[112] Mahjour B., Zhang R., Shen Y. (2023). Rapid planning and analysis of high-throughput experiment arrays for reaction discovery. Nat Commun.

[113] de Munnik M., Lithgow J., Brewitz L. (2023). αβ,α′β′-Diepoxyketones are mechanism-based inhibitors of nucleophilic cysteine enzymes. Chem Commun.

[114] Schrödinger LLC., The PyMOL Molecular Graphics System, Version 2.3.0.

[115] Dunbrack R.L., Cohen F.E. (1997). Bayesian statistical analysis of protein side-chain rotamer preferences. Protein Sci.

[116] Lindorff-Larsen K., Piana S., Palmo K. (2010). Improved side-chain torsion potentials for the Amber ff99SB protein force field. Proteins.

[117] Abraham M.J., Murtola T., Schulz R. (2015). GROMACS: high performance molecular simulations through multi-level parallelism from laptops to supercomputers. SoftwareX.

[118] Jorgensen W.L., Chandrasekhar J., Madura J.D., Impey R.W., Klein M.L. (1983). Comparison of simple potential functions for simulating liquid water. J Chem Phys.

[119] Hoops S.C., Anderson K.W., Merz K.M. (1991). Force field design for metalloproteins. J Am Chem Soc.

[120] Bayly C.I., Cieplak P., Cornell W.D., Kollman P.A. (1993). A well-behaved electrostatic potential based method using charge restraints for deriving atomic charges: the RESP model. J Phys Chem.

[121] Cornell W.D., Cieplak P., Bayly C.I., Kollman P.A. (1993). Application of RESP charges to calculate conformational energies, hydrogen bond energies, and free energies of solvation. J Am Chem Soc.

[122] Cornell W.D., Cieplak P., Bayly C.I. (1995). A second generation force field for the simulation of proteins, nucleic acids, and organic molecules. J Am Chem Soc.

[123] Frisch M.J., Trucks G.W., Schlegel H.B. (2016). Gaussian 16 Rev. A.03.

[124] Case D.A., Ben-Shalom I.Y., Brozell S.R. (2018). AMBER 2018.

[125] Bussi G., Donadio D., Parrinello M. (2007). Canonical sampling through velocity rescaling. J Chem Phys.

[126] Nosé S., Klein M.L. (1983). Constant pressure molecular dynamics for molecular systems. Mol Phys.

[127] Parrinello M., Rahman A. (1981). Polymorphic transitions in single crystals: A new molecular dynamics method. J Appl Phys.

[128] Darden T., York D., Pedersen L. (1993). Particle mesh Ewald: an *N*⋅log(*N*) method for Ewald sums in large systems. J Chem Phys.

[129] Essmann U., Perera L., Berkowitz M.L., Darden T., Lee H., Pedersen L.G. (1995). A smooth particle mesh Ewald method. J Chem Phys.

[130] Daura X., Gademann K., Jaun B., Seebach D., van Gunsteren W.F., Mark A.E. (1999). Peptide folding: when simulation meets experiment. Angew Chem Int Ed.

[131] Srinivasan J., Cheatham T.E., Cieplak P., Kollman P.A., Case D.A. (1998). Continuum solvent studies of the stability of DNA, RNA, and phosphoramidate−DNA helices. J Am Chem Soc.

[132] Kollman P.A., Massova I., Reyes C. (2000). Calculating structures and free energies of complex molecules: combining molecular mechanics and continuum models. Acc Chem Res.

[133] Gohlke H., Kiel C., Case D.A. (2003). Insights into protein–protein binding by binding free energy calculation and free energy decomposition for the Ras-Raf and Ras–RalGDS complexes. J Mol Biol.

[134] Metz A., Pfleger C., Kopitz H., Pfeiffer S. (2012). Hot spots and transient pockets: predicting the determinants of small-molecule binding to a protein–protein interface. J Chem Inf Model.

[135] Miller B.R., McGee T.D., Swails J.M., Homeyer N., Gohlke H., Roitberg A.E. (2012). MMPBSA.py: an efficient program for end-state free energy calculations. J Chem Theory Comput.

[136] Onufriev A., Bashford D., Case D.A. (2004). Exploring protein native states and large-scale conformational changes with a modified generalized born model. Proteins.

