# Supplementary material for: Mass spectrometric assays monitoring the deubiquitinase activity of the SARS-CoV-2 papain-like protease inform on the basis of substrate selectivity and have utility for substrate identification
Source: Bioorg Med Chem. 2023 Nov 15;95:117498. doi: 10.1016/j.bmc.2023.117498 (PMC10933793; doi:10.1016/j.bmc.2023.117498)
Supplement: Supplementary data 1 [file mmc1.pdf]

Supporting Information

Mass spectrometric assays monitoring the deubiquitinase activity of the SARS-CoV-2 papain-like protease inform on the basis of substrate selectivity and have utility for substrate identification

Lennart Brewitz[1,2,*], H. T. Henry Chan[1], Petra Lukacik[3,4], Claire Strain-Damerell[3,4], Martin A. Walsh[3,4], Fernanda Duarte[1], and Christopher J. Schofield[1,2,*]

[1]*Chemistry Research Laboratory, Department of Chemistry, University of Oxford, 12 Mansfield Road, OX1 3TA, Oxford, United Kingdom.*

[2]*The Ineos Oxford Institute for Antimicrobial Research, Department of Chemistry, University of Oxford, 12 Mansfield Road, OX1 3TA, Oxford, United Kingdom.*

[3]*Diamond Light Source Ltd., Harwell Science and Innovation Campus, OX11 0DE, Didcot, United Kingdom.*

[4]*Research Complex at Harwell, Harwell Science and Innovation Campus, OX11 0FA, Didcot, United Kingdom.*

*E-mail: lennart.brewitz@chem.ox.ac.uk and christopher.schofield@chem.ox.ac.uk

Table of contents

1. Supporting figures S2-S46
2. Supporting tables S47-S48
3. References S49

1. Supporting figures

Supporting Figure S1. The N^{ε}-lysine-branched oligopeptides used in this study (continues on the following 22 pages). Sequences of N^{ε}-lysine-branched oligopeptides used in this work as potential SARS-CoV-2 PL^{pro} substrates; peptide sequences based on the coding sequences of human proteins post-translationally modified with ubiquitin or ubiquitin-like modifiers. Peptides were synthesized with C-terminal amides by solid phase peptide synthesis (SPPS) and purified by HPLC as described in the Experimental section; the peptide purity (>90%) was determined using HPLC. The anticipated peptide masses were confirmed using solid phase extraction coupled to mass spectrometry (SPE-MS) in LCMS-grade water; SPE-MS conditions are given in the Experimental section.

(**I**) (**a**) Sequence and purification characteristics of the K_{193}-branched $IRF3_{189\text{-}197}$-ISG15 oligopeptide **4**; (**b**) mass spectrum (SPE-MS) of **4** (2.0 µM) in water. m/z = 1263.75 corresponds to the +2 charge state of **4**, note that **4** ionizes in the +2 charge state as the $2H^{+}$ ion and, less abundantly, as the $Na^{+}H^{+}$ ion; the enlarged region shows the m/z +2 peak. m/z = 842.84 corresponds to the +3 charge state of **4**, m/z = 632.38 corresponds to the +4 charge state of **4**, and m/z = 506.11 corresponds to the +5 charge state of **4**. The peptide is estimated to be >90% pure based on HPLC analysis. Note, m/z values referred to are for the most abundant isotope.

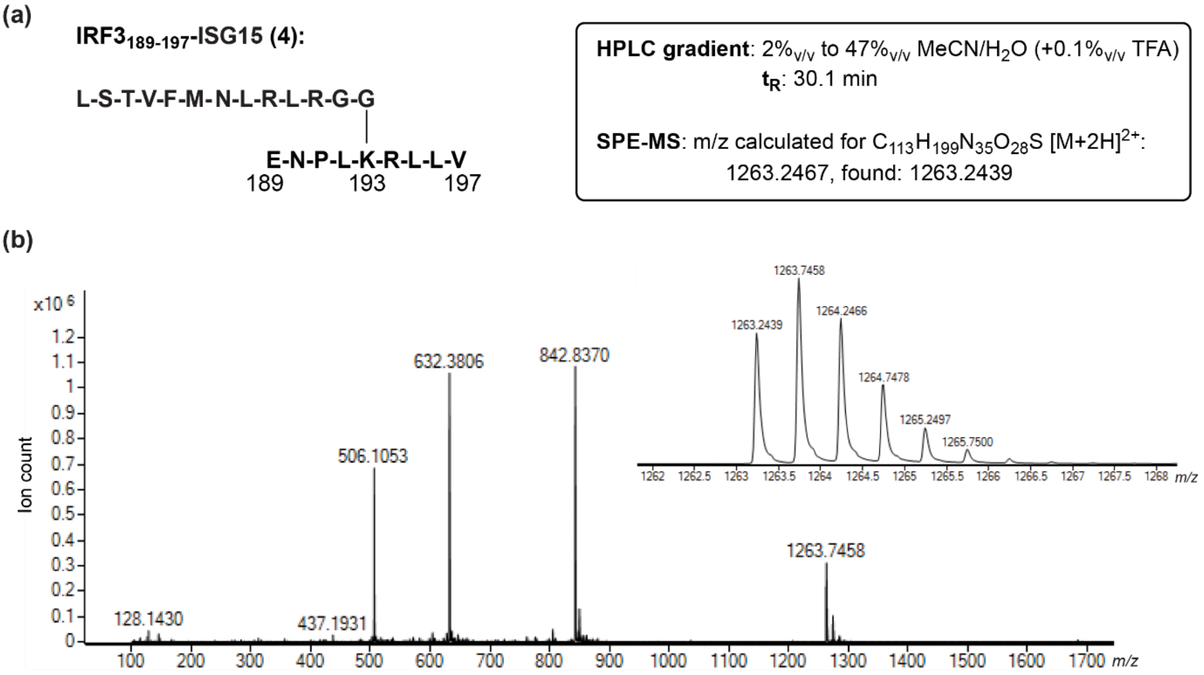

(**II**) (**a**) Sequence and purification characteristics of the ISG15-derived N-terminally *N*-acetylated inert standard peptide Ac-LSTVFMNLRLRGG-NH_2 (**5**); (**b**) mass spectrum (SPE-MS) of **5** (2.0 μM) in water. m/z = 752.93 corresponds to the +2 charge state of **5**, note that **5** ionizes in the +2 charge state as the $2H^+$ ion and, less abundant, as the H^+Na^+ ion; the enlarged region shows the m/z +2 peak. m/z = 1504.84 corresponds to the +1 charge state of **5**. The peptide is estimated to be >90% pure based on HPLC analysis. Note, m/z values referred to are for the most abundant isotope.

(a)

N-terminally *N*-acetylated ISG15-derived internal standard (5):

Ac-**L-S-T-V-F-M-N-L-R-L-R-G-G**

HPLC gradient: 2%$_{v/v}$ to 47%$_{v/v}$ MeCN/H_2O (+0.1%$_{v/v}$ TFA)
t_R: 26.0 min

SPE-MS: m/z calculated for $C_{66}H_{115}N_{21}O_{17}$ $[M+2H]^{2+}$: 752.9245, found: 752.9332

(b)

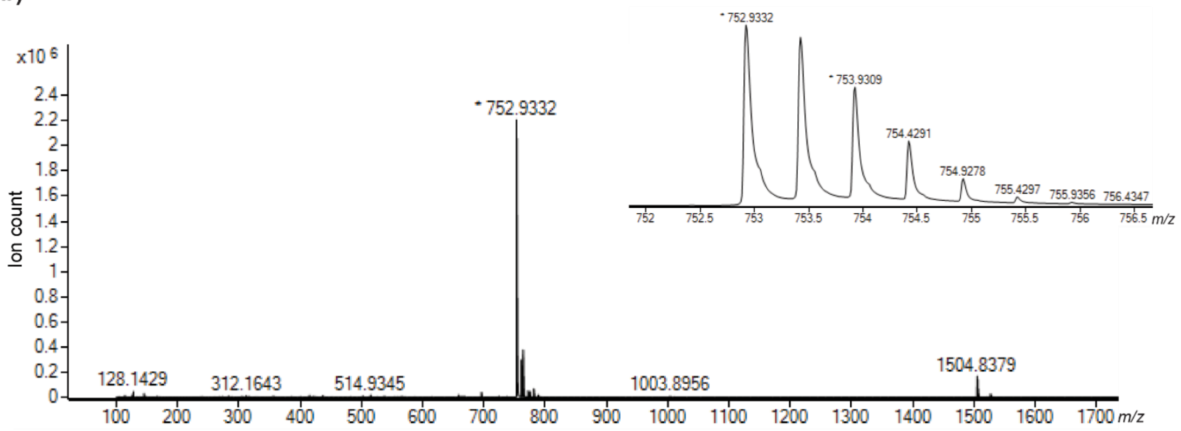

(**III**) (**a**) Sequence and purification characteristics of the K_{193}-branched $IRF3_{189\text{-}193}$-ISG15 oligopeptide **6**; (**b**) mass spectrum (SPE-MS) of **6** (2.0 µM) in water. m/z = 1023.08 corresponds to the +2 charge state of **6**, note that **6** ionizes in the +2 charge state as the $2H^+$ ion and, less abundantly, as the Na^+H^+ and as $2Na^+$ ions; the enlarged region shows the m/z +2 peak. m/z = 682.39 corresponds to the +3 charge state of **6**, and m/z = 512.05 corresponds to the +4 charge state of **6**. The peptide is estimated to be >90% pure based on HPLC analysis. Note, m/z values referred to are for the most abundant isotope.

(a)

$IRF3_{189\text{-}193}$-ISG15 (6):

L-S-T-V-F-M-N-L-R-L-R-G-G

E-N-P-L-K
189 193

HPLC gradient: $2\%_{v/v}$ to $47\%_{v/v}$ MeCN/H_2O (+$0.1\%_{v/v}$ TFA)
t_R: 26.0 min

SPE-MS: m/z calculated for $C_{90}H_{156}N_{28}O_{24}S$ $[M+2H]^{2+}$: 1022.5779, found: 1022.5789

(b)

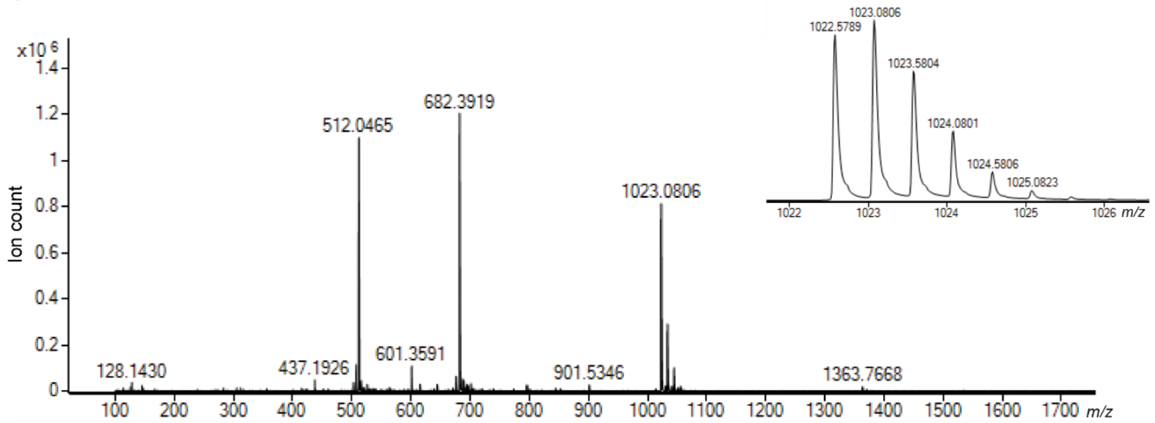

(**IV**) (**a**) Sequence and purification characteristics of the K_{193}-branched $IRF3_{192\text{-}193}$-ISG15 oligopeptide **7**; (b) mass spectrum (SPE-MS) of **7** (2.0 μM) in water. m/z = 852.51 corresponds to the +2 charge state of **7**, note that **7** ionizes in the +2 charge state as the $2H^+$ ion and, less abundantly, as the Na^+H^+ ion; the enlarged region shows the m/z +2 peak. m/z = 1704.01 corresponds to the +1 charge state of **7**, m/z = 568.68 corresponds to the +3 charge state of **7**, and m/z = 426.76 corresponds to the +4 charge state of **7**. The peptide is estimated to be >90% pure based on HPLC analysis. Note, m/z values referred to are for the most abundant isotope.

(a)

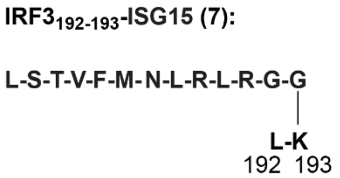

HPLC gradient: 2%$_{v/v}$ to 47%$_{v/v}$ MeCN/H_2O (+0.1%$_{v/v}$ TFA)
t_R: 25.6 min

SPE-MS: m/z calculated for $C_{76}H_{136}N_{24}O_{18}S$ $[M+2H]^{2+}$: 852.5087, found: 852.5121

(b)

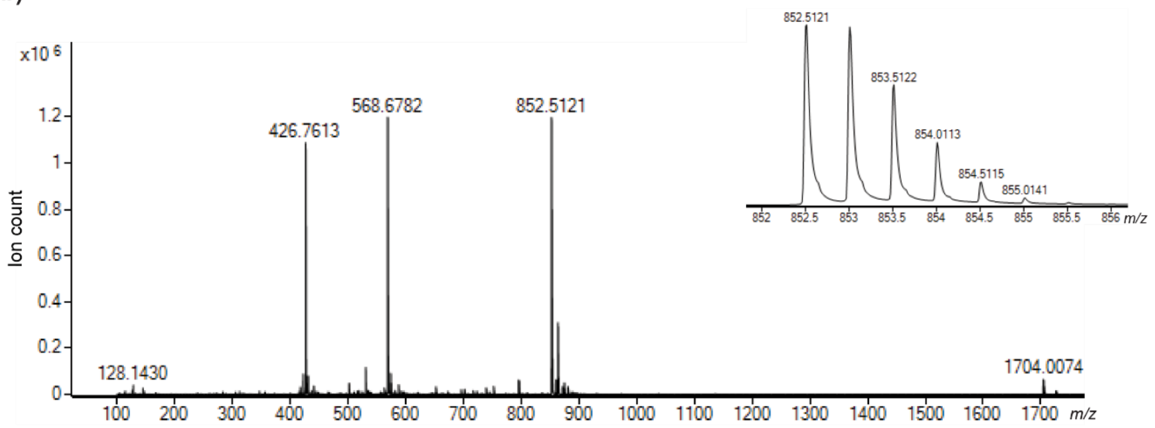

(**V**) (**a**) Sequence and purification characteristics of the K_{193}-branched $IRF3_{185\text{-}200}$-ISG15 oligopeptide **8**; (**b**) mass spectrum (SPE-MS) of **8** (2.0 µM) in water. m/z = 1055.27 corresponds to the +3 charge state of **8**, note that **8** ionizes in the +3 charge state as the $3H^+$ ion and, less abundantly, as the $2H^+Na^+$ and $2Na^+H^+$ ions; the enlarged region shows the m/z +3 peak. m/z = 1582.40 corresponds to the +2 charge state of **8**, m/z = 791.71 corresponds to the +4 charge state of **8**, and m/z = 633.57 corresponds to the +5 charge state of **8**. The peptide is estimated to be >90% pure based on HPLC analysis. Note, m/z values referred to are for the most abundant isotope.

(a)

$IRF3_{185\text{-}200}$-ISG15 (8):

L-S-T-V-F-M-N-L-R-L-R-G-G

L-G-P-S-E-N-P-L-K-R-L-L-V-P-G-E

185 193 200

HPLC gradient: $2\%_{v/v}$ to $47\%_{v/v}$ MeCN/H_2O (+$0.1\%_{v/v}$ TFA)
t_R: 31.2 min

SPE-MS: m/z calculated for $C_{141}H_{243}N_{42}O_{38}S$ $[M+3H]^{3+}$: 1054.9360, found: 1054.9358

(b)

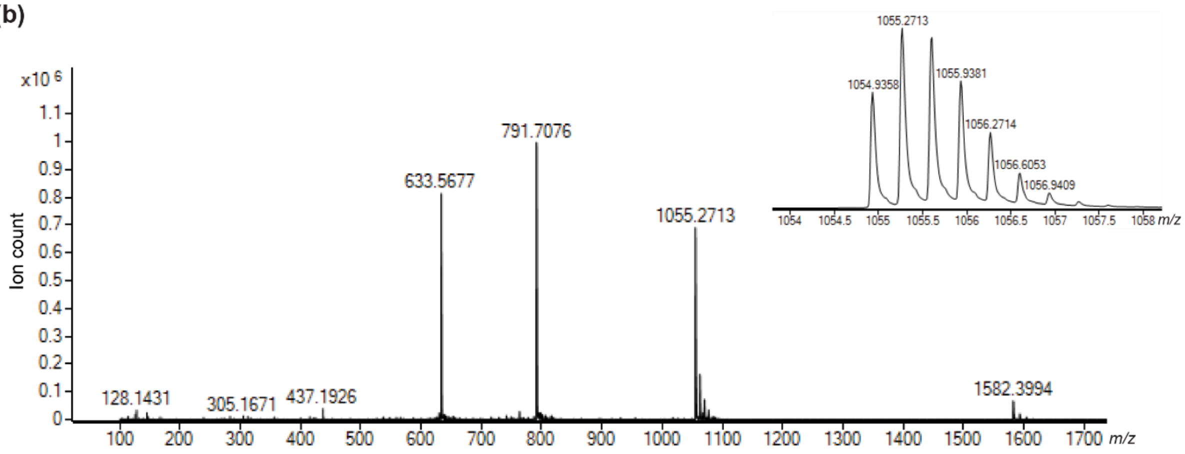

(**VI**) (**a**) Sequence and purification characteristics of the K_{193}-branched IRF3$_{189\text{-}197}$-ISG15$_{1\text{-}18}$ oligopeptide **9**; (**b**) mass spectrum (SPE-MS) of **9** (2.0 µM) in water. m/z = 1028.94 corresponds to the +3 charge state of **9**, note that **9** ionizes in the +3 charge state as the $3H^+$ ion and, less abundantly, as the $2H^+Na^+$ ion; the enlarged region shows the m/z +3 peak. m/z = 1542.90 corresponds to the +2 charge state of **9**, m/z = 771.96 corresponds to the +4 charge state of **9**, m/z = 617.77 corresponds to the +5 charge state of **9**, and m/z = 514.97 corresponds to the +6 charge state of **9**. The peptide is estimated to be >90% pure based on HPLC analysis. Note, m/z values referred to are for the most abundant isotope.

(a)

IRF3$_{189\text{-}197}$-ISG15$_{1\text{-}18}$ (9):

Y-G-L-K-P-L-S-T-V-F-M-N-L-R-L-R-G-G

E-N-P-L-K-R-L-L-V

189 193 197

HPLC gradient: 2%$_{v/v}$ to 47%$_{v/v}$ MeCN/H_2O (+0.1%$_{v/v}$ TFA)

t_R: 34.0 min

SPE-MS: m/z calculated for $C_{141}H_{242}N_{41}O_{34}S$ $[M+3H]^{3+}$: 1028.6057, found: 1028.6049

(b)

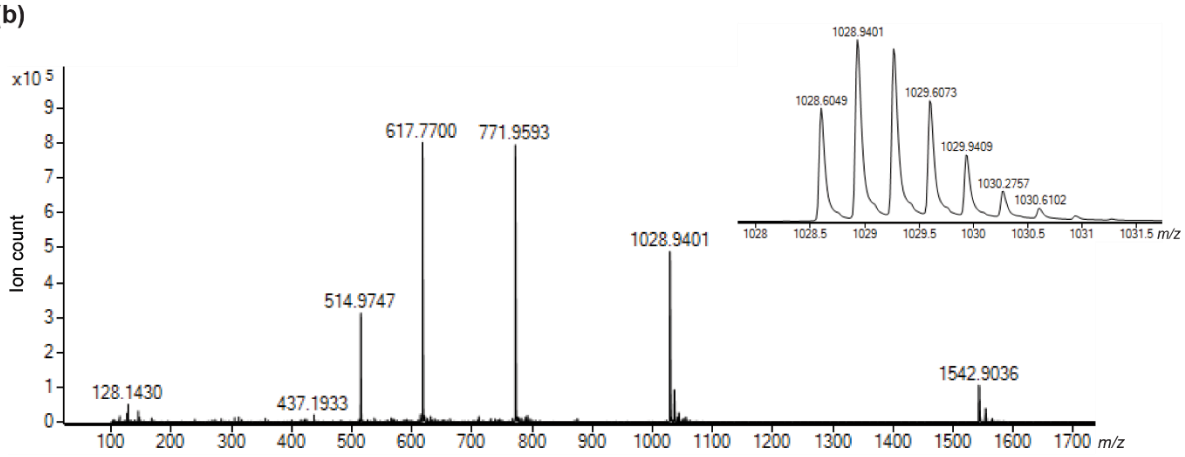

(**VII**) (**a**) Sequence and purification characteristics of the K_{193}-branched $IRF3_{189\text{-}197}$-$ISG15_{1\text{-}7}$ oligopeptide **10**; (**b**) mass spectrum (SPE-MS) of **10** (2.0 μM) in water. m/z = 924.58 corresponds to the +2 charge state of **10**, note that **10** ionizes in the +2 charge state as the $2H^+$ ion and, less abundantly, as the H^+Na^+ ion; the enlarged region shows the m/z +2 peak. m/z = 616.72 corresponds to the +3 charge state of **10**, and m/z = 462.80 corresponds to the +4 charge state of **10**. The peptide is estimated to be >90% pure based on HPLC analysis. Note, m/z values referred to are for the most abundant isotope.

(a)

$IRF3_{189\text{-}197}$-$ISG15_{1\text{-}7}$ (10):

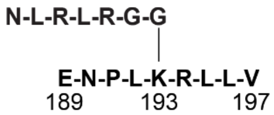

HPLC gradient: $2\%_{v/v}$ to $47\%_{v/v}$ MeCN/H_2O (+$0.1\%_{v/v}$ TFA)
t_R: 28.3 min

SPE-MS: m/z calculated for $C_{81}H_{149}N_{29}O_{20}$ $[M+2H]^{2+}$: 924.0762, found: 924.0772

(b)

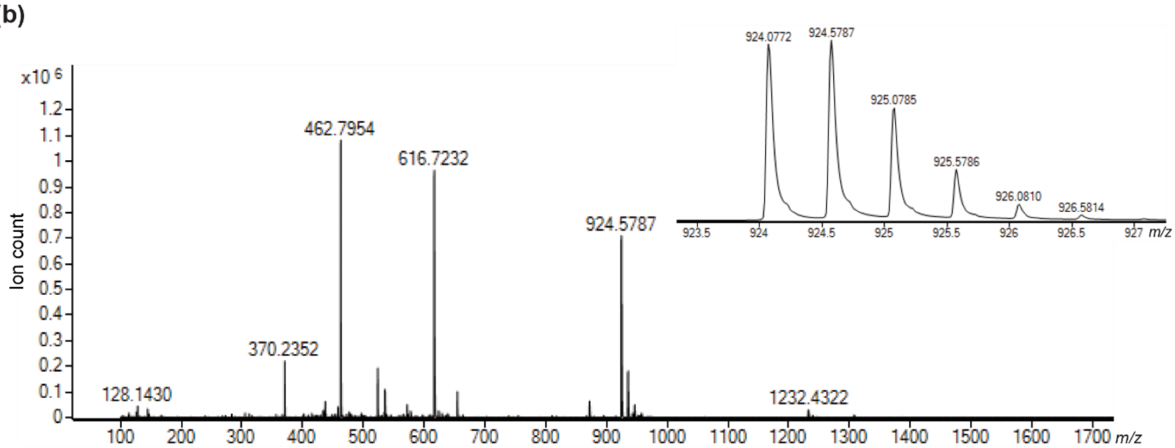

(**VIII**) (**a**) Sequence and purification characteristics of the K_{193}-branched IRF$3_{189\text{-}197}$-ISG$15_{1\text{-}4}$ oligopeptide **11**; (**b**) mass spectrum (SPE-MS) of **11** (2.0 µM) in water. m/z = 732.47 corresponds to the +2 charge state of **11**, note that **11** ionizes in the +2 charge state as the $2H^+$ ion and, less abundantly, as the H^+Na^+ ion; the enlarged region shows the m/z +2 peak. m/z = 1463.91 corresponds to the +1 charge state of **11**, m/z = 488.65 corresponds to the +3 charge state of **11**, and m/z = 366.74 corresponds to the +4 charge state of **11**. The peptide is estimated to be >90% pure based on HPLC analysis. Note, m/z values referred to are for the most abundant isotope.

(a)

IRF3$_{189\text{-}197}$-ISG15 (11):

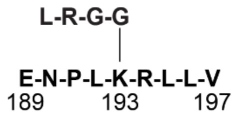

HPLC gradient: 2%$_{v/v}$ to 47%$_{v/v}$ MeCN/H_2O (+0.1%$_{v/v}$ TFA)
t_R: 28.0 min

SPE-MS: m/z calculated for $C_{65}H_{120}N_{22}O_{16}$ $[M+2H]^{2+}$: 732.4621, found: 732.4658

(b)

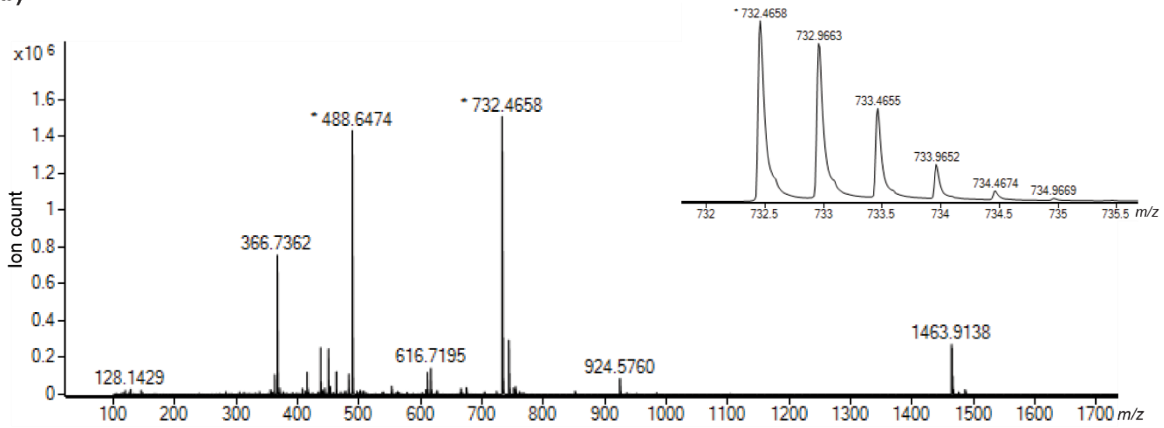

(**IX**) (**a**) Sequence and purification characteristics of the IRF3$_{189\text{-}193}$-derived N-terminally *N*-acetylated inert standard peptide Ac-ENPLKRLLV-NH_2 (**12**); (**b**) mass spectrum (SPE-MS) of **12** (2.0 μM) in water. m/z = 1122.70 corresponds to the +1 charge state of **12**, note that **12** ionizes in the +1 charge state as the H^+ ion and, less abundant, as the Na^+ ion; the enlarged region shows the m/z +1 peak. m/z = 561.86 corresponds to the +2 charge state of **12**. The peptide is estimated to be >90% pure based on HPLC analysis. Note, m/z values referred to are for the most abundant isotope.

(a)

N-terminally *N*-acetylated
IRF3$_{189\text{-}197}$-derived internal standard (12):

Ac-**E-N-P-L-K-R-L-L-V**
189 193 197

HPLC gradient: 2%$_{v/v}$ to 47%$_{v/v}$ MeCN/H_2O (+0.1%$_{v/v}$ TFA)
t_R: 26.3 min

SPE-MS: m/z calculated for $C_{51}H_{92}N_{15}O_{13}$ $[M+H]^+$: 1122.6994, found: 1122.7011

(b)

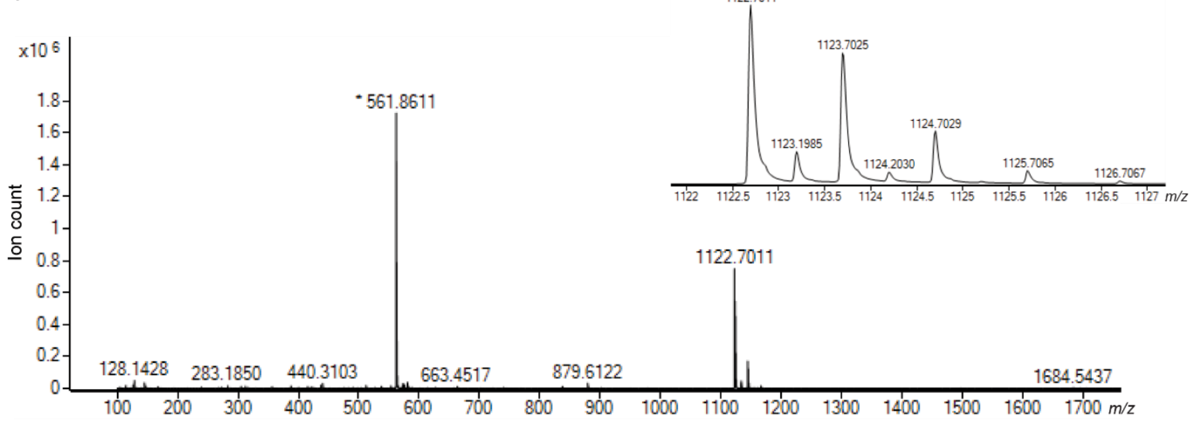

(**X**) (**a**) Sequence and purification characteristics of the K_{360}-branched IRF3$_{357\text{-}364}$-ISG15 oligopeptide **13**; (**b**) mass spectrum (SPE-MS) of **13** (2.0 µM) in water. m/z = 1238.20 corresponds to the +2 charge state of **13**, note that **13** ionizes in the +2 charge state as the $2H^+$ ion and, less abundantly, as the H^+Na^+ ion; the enlarged region shows the m/z +2 peak. m/z = 825.81 corresponds to the +3 charge state of **13**, m/z = 619.61 corresponds to the +4 charge state of **13**, and m/z = 495.89 corresponds to the +5 charge state of **13**. The peptide is estimated to be >75% pure based on HPLC analysis. Note, m/z values referred to are for the most abundant isotope.

(a)

IRF3$_{357\text{-}364}$-ISG15 (13):

L-S-T-V-F-M-N-L-R-L-R-G-G

P-W-T-K-R-L-V-M

357 360 364

HPLC gradient: 2%$_{v/v}$ to 47%$_{v/v}$ MeCN/H_2O (+0.1%$_{v/v}$ TFA)
t_R: 29.2 min

SPE-MS: m/z calculated for $C_{112}H_{190}N_{34}O_{25}S_2$ $[M+2H]^{2+}$: 1237.7036, found: 1237.7001

(b)

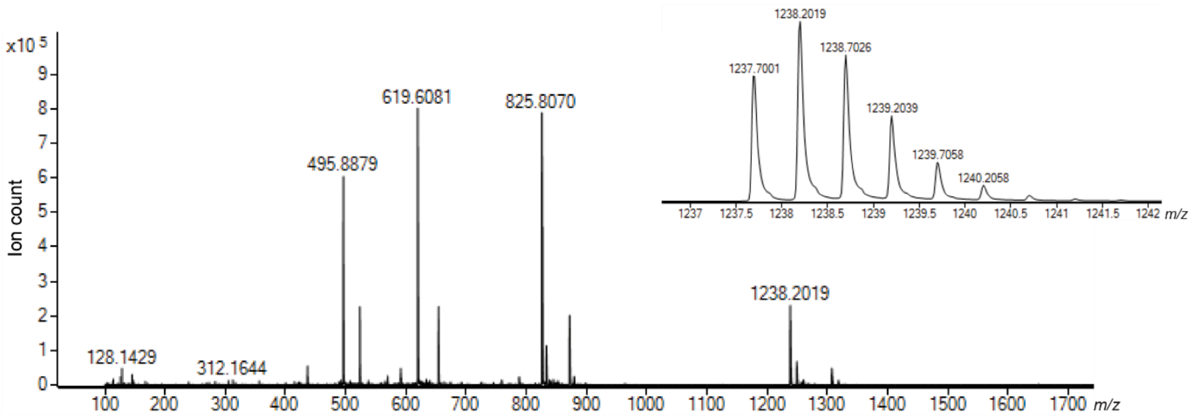

(**XI**) (**a**) Sequence and purification characteristics of the K_{366}-branched $IRF3_{362\text{-}370}$-ISG15 oligopeptide **14**; (**b**) mass spectrum (SPE-MS) of **14** (2.0 µM) in water. m/z = 1215.72 corresponds to the +2 charge state of **14**, note that **14** ionizes in the +2 charge state as the $2H^+$ ion and, less abundantly, as the H^+Na^+ and $2Na^+$ ions; the enlarged region shows the m/z +2 peak. m/z = 810.82 corresponds to the +3 charge state of **14**, and m/z = 608.37 corresponds to the +4 charge state of **14**. The peptide is estimated to be >90% pure based on HPLC analysis. Note, m/z values referred to are for the most abundant isotope.

(a)

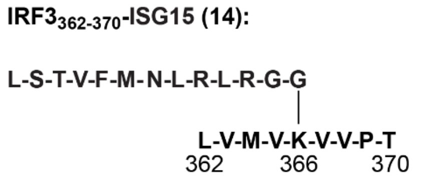

HPLC gradient: $2\%_{v/v}$ to $47\%_{v/v}$ MeCN/H_2O (+$0.1\%_{v/v}$ TFA)
t_R: 29.4 min

SPE-MS: m/z calculated for $C_{110}H_{195}N_{31}O_{26}S_2$ $[M+2H]^{2+}$: 1215.2160, found: 1215.2135

(b)

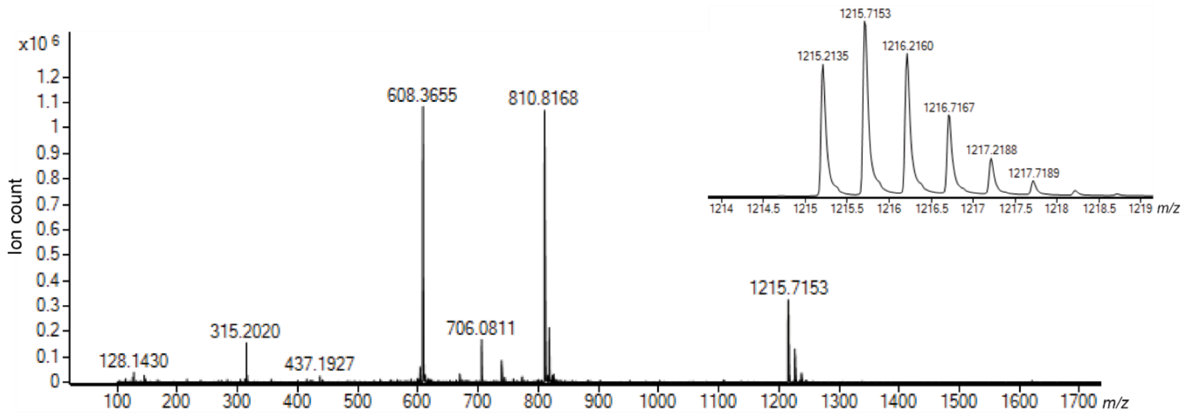

(**XII**) (**a**) Sequence and purification characteristics of the K_{193}-branched IRF3$_{189\text{-}197}$-Ub oligopeptide **15**; (**b**) mass spectrum (SPE-MS) of **15** (2.0 µM) in water. m/z = 1257.26 corresponds to the +2 charge state of **15**, note that **15** ionizes in the +2 charge state as the $2H^+$ ion and, less abundantly, as the Na^+H^+ and $2Na^+$ ions; the enlarged region shows the m/z +2 peak. m/z = 838.51 corresponds to the +3 charge state of **15**, m/z = 629.14 corresponds to the +4 charge state of **15**, and m/z = 503.51 corresponds to the +5 charge state of **15**. The peptide is estimated to be >90% pure based on HPLC analysis. Note, m/z values referred to are for the most abundant isotope.

(a)

IRF3$_{189\text{-}197}$-Ub (15):

E-S-T-L-H-L-V-L-R-L-R-G-G

|

E-N-P-L-K-R-L-L-V

189 193 197

HPLC gradient: 2%$_{v/v}$ to 47%$_{v/v}$ MeCN/H_2O (+0.1%$_{v/v}$ TFA)

t_R: 29.3 min

SPE-MS: m/z calculated for $C_{112}H_{200}N_{36}O_{29}$ $[M+2H]^{2+}$: 1256.7636, found: 1256.7613

(b)

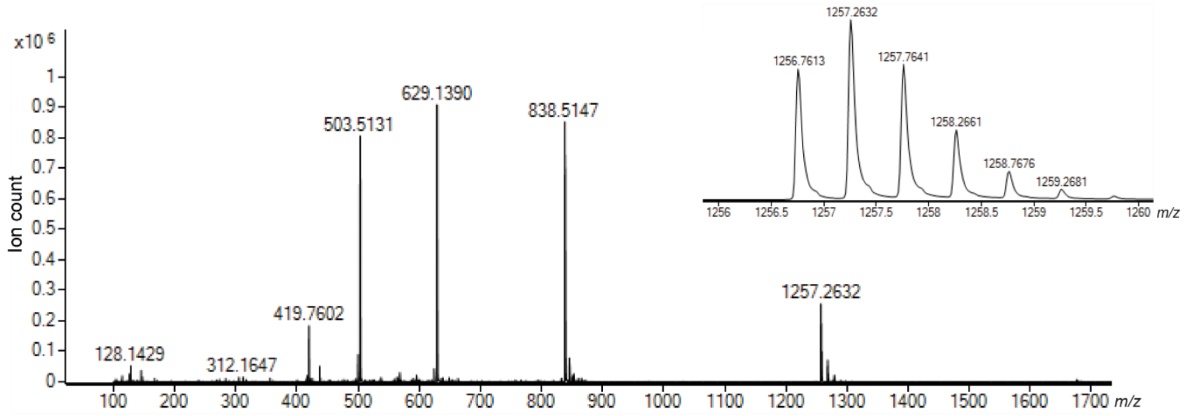

(**XIII**) (**a**) Sequence and purification characteristics of the K_{193}-branched IRF3$_{189\text{-}197}$-NEDD8 oligopeptide **16**; (**b**) mass spectrum (SPE-MS) of **16** (2.0 μM) in water. m/z = 1177.73 corresponds to the +2 charge state of **16**, note that **16** ionizes in the +2 charge state as the $2H^+$ ion and, less abundantly, as the Na^+H^+ and $2Na^+$ ions; the enlarged region shows the m/z +2 peak. m/z = 785.49 corresponds to the +3 charge state of **16**, m/z = 589.37 corresponds to the +4 charge state of **16**, and m/z = 471.70 corresponds to the +5 charge state of **16**. The peptide is estimated to be >90% pure based on HPLC analysis. Note, m/z values referred to are for the most abundant isotope.

(a)

IRF3$_{189\text{-}197}$-NEDD8 (16):

G-S-V-L-H-L-V-L-A-L-R-G-G

E-N-P-L-K-R-L-L-V (branched at K)

189 193 197

HPLC gradient: 2%$_{v/v}$ to 47%$_{v/v}$ MeCN/H_2O (+0.1%$_{v/v}$ TFA)
t_R: 33.4 min

SPE-MS: m/z calculated for $C_{107}H_{191}N_{33}O_{26}$ $[M+2H]^{2+}$: 1177.2314, found: 1177.2300

(b)

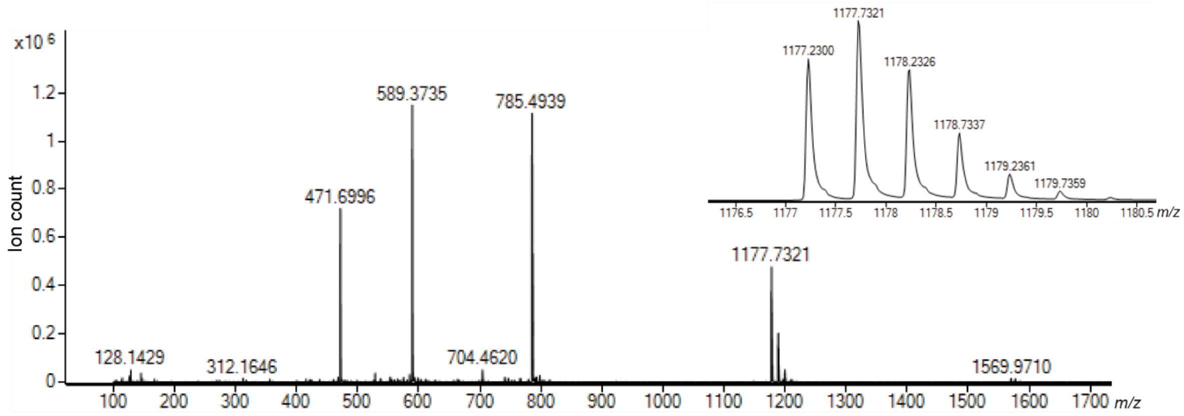

(**XIV**) (**a**) Sequence and purification characteristics of the K$_{193}$-branched IRF3$_{189-197}$-URM1 oligopeptide **17**; (**b**) mass spectrum (SPE-MS) of **17** (2.0 μM) in water. m/z = 1218.69 corresponds to the +2 charge state of **17**, note that **17** ionizes in the +2 charge state as the 2H$^+$ ion and, less abundantly, as the Na$^+$H$^+$ and 2Na$^+$ ions; the enlarged region shows the m/z +2 peak. m/z = 812.80 corresponds to the +3 charge state of **17**, and m/z = 609.85 corresponds to the +4 charge state of **17**. The peptide is estimated to be >85% pure based on HPLC analysis. Note, m/z values referred to are for the most abundant isotope.

(a)

IRF3$_{189-197}$-URM1 (17):

Q-D-S-V-L-F-I-S-T-L-H-G-G

E-N-P-L-K-R-L-L-V (K branched to the terminal G)

189 193 197

HPLC gradient: 2%$_{v/v}$ to 47%$_{v/v}$ MeCN/H_2O (+0.1%$_{v/v}$ TFA)

t_R: 30.2 min

SPE-MS: m/z calculated for $C_{110}H_{185}N_{31}O_{31}$ $[M+2H]^{2+}$: 1218.1921, found: 1218.1907

(b)

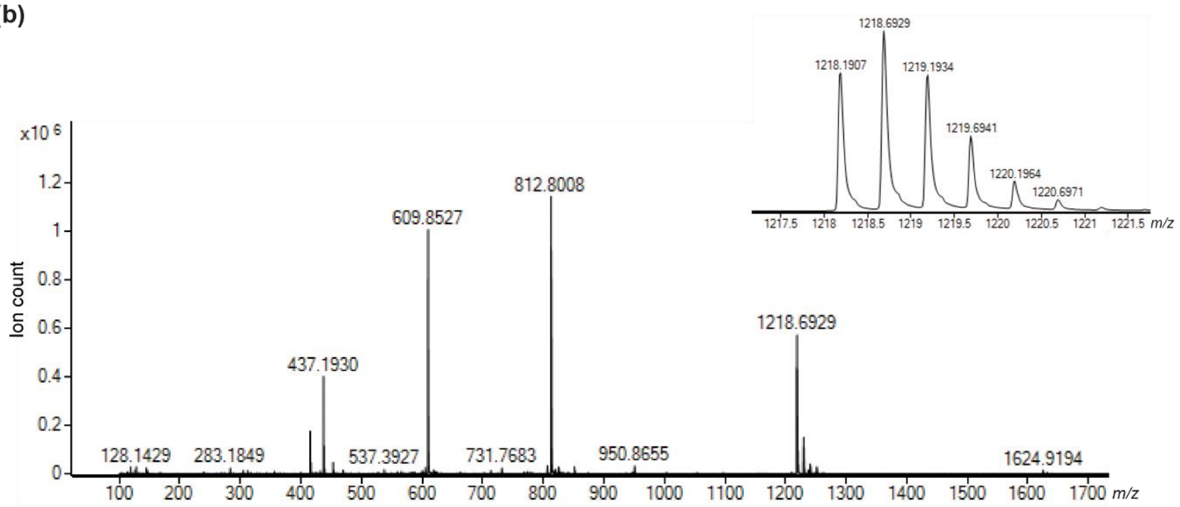

(**XV**) (**a**) Sequence and purification characteristics of the K_{193}-branched $IRF3_{189\text{-}197}$-SUMO1 oligopeptide **18**; (**b**) mass spectrum (SPE-MS) of **18** (2.0 µM) in water. m/z = 1265.17 corresponds to the +2 charge state of **18**, note that **18** ionizes in the +2 charge state as the $2H^+$ ion and, less abundantly, as the Na^+H^+ ion; the enlarged region shows the m/z +2 peak. m/z = 843.79 corresponds to the +3 charge state of **18**. The peptide is estimated to be >85% pure based on HPLC analysis. Note, m/z values referred to are for the most abundant isotope.

(a)

$IRF3_{189\text{-}197}$-SUMO1 (18):

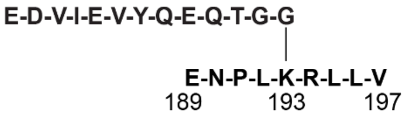

HPLC gradient: $2\%_{v/v}$ to $47\%_{v/v}$ MeCN/H_2O (+$0.1\%_{v/v}$ TFA)
t_R: 29.4 min

SPE-MS: m/z calculated for $C_{111}H_{184}N_{30}O_{37}$ $[M+2H]^{2+}$: 1264.6714, found: 1264.6725

(b)

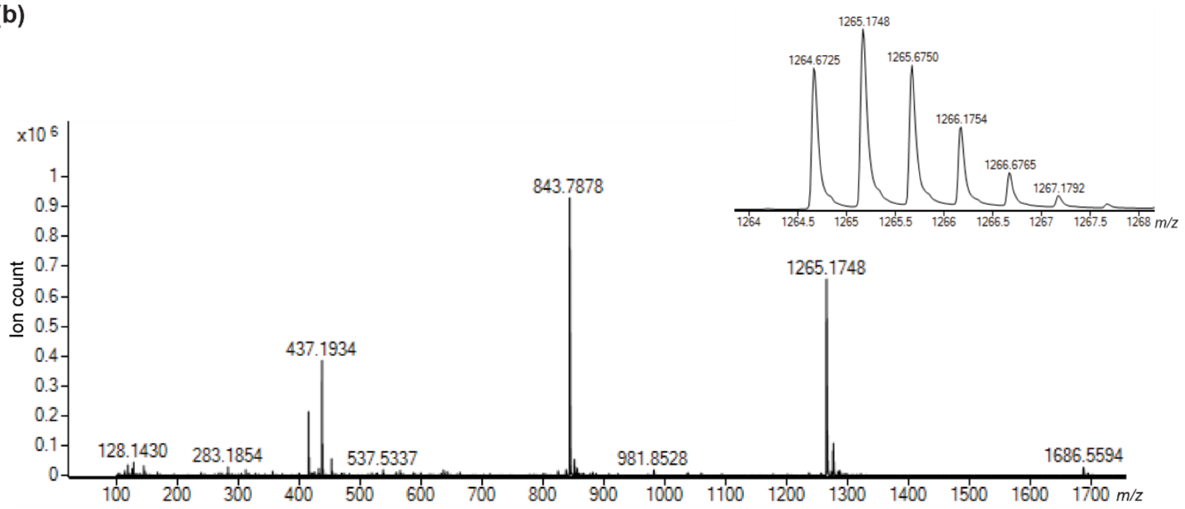

(**XVI**) (**a**) Sequence and purification characteristics of the K_{134}-branched $4EHP_{132\text{-}139}$-ISG15 oligopeptide **19**; (**b**) mass spectrum (SPE-MS) of **19** (2.0 µM) in water. m/z = 1194.20 corresponds to the +2 charge state of **19**, note that **19** ionizes in the +2 charge state as the $2H^{+}$ ion and, less abundantly, as the $H^{+}Na^{+}$ ion; the enlarged region shows the m/z +2 peak. m/z = 796.48 corresponds to the +3 charge state of **19**, m/z = 597.61 corresponds to the +4 charge state of **19**, and m/z = 478.29 corresponds to the +5 charge state of **19**. The peptide is estimated to be >90% pure based on HPLC analysis. Note, m/z values referred to are for the most abundant isotope.

(a)

$4EHP_{132\text{-}139}$-ISG15 (19):

L-S-T-V-F-M-N-L-R-L-R-G-G
|
G-G-K-W-I-I-R-L
132 134 139

HPLC gradient: $2\%_{v/v}$ to $47\%_{v/v}$ MeCN/H_2O (+$0.1\%_{v/v}$ TFA)
t_R: 30.8 min

SPE-MS: m/z calculated for $C_{109}H_{186}N_{34}O_{24}S$ $[M+2H]^{2+}$: 1139.7045, found: 1193.7020

(b)

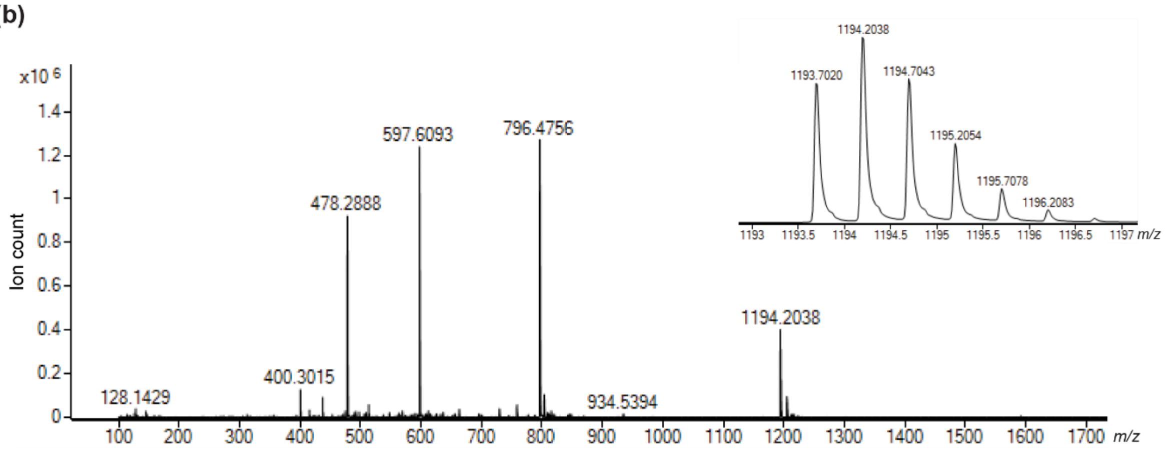

(**XVII**) (**a**) Sequence and purification characteristics of the K_{222}-branched $4EHP_{218\text{-}227}$-ISG15 oligopeptide **20**; (**b**) mass spectrum (SPE-MS) of **20** (2.0 µM) in water. m/z = 1281.71 corresponds to the +2 charge state of **20**, note that **20** ionizes in the +2 charge state as the $2H^+$ ion and, less abundantly, as the H^+Na^+ and $2Na^+$ ions; the enlarged region shows the m/z +2 peak. m/z = 854.81 corresponds to the +3 charge state of **20**, m/z = 641.36 corresponds to the +4 charge state of **20**, and m/z = 513.29 corresponds to the +5 charge state of **20**. The peptide is estimated to be >90% pure based on HPLC analysis. Note, m/z values referred to are for the most abundant isotope.

(a)

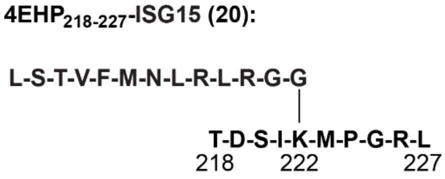

HPLC gradient: $2\%_{v/v}$ to $47\%_{v/v}$ MeCN/H_2O (+$0.1\%_{v/v}$ TFA)
t_R: 26.7 min

SPE-MS: m/z calculated for $C_{111}H_{195}N_{35}O_{30}S_2$ $[M+2H]^{2+}$: 1281.2120, found: 1281.2082

(b)

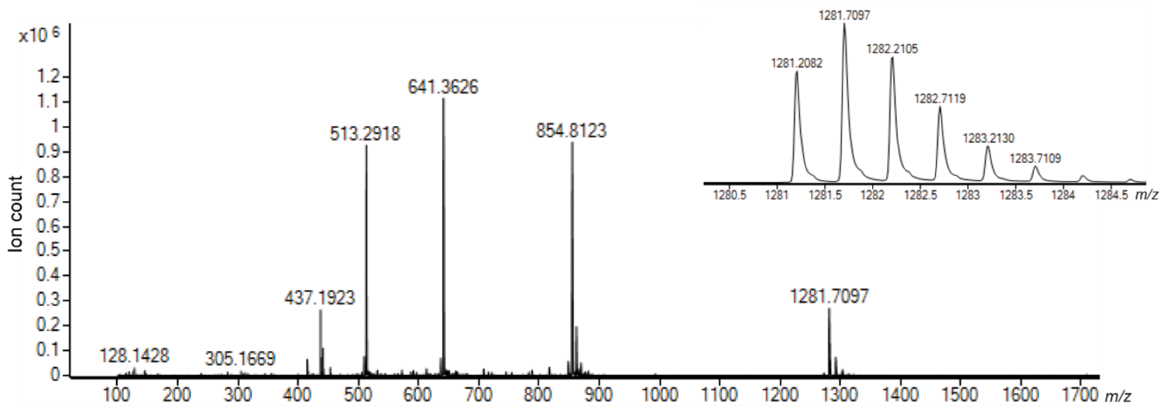

(**XVIII**) (**a**) Sequence and purification characteristics of the K_{287}-branched $TMEM59_{285\text{-}291}$-Ub oligopeptide **21**; (**b**) mass spectrum (SPE-MS) of **21** (2.0 μM) in water. m/z = 1191.68 corresponds to the +2 charge state of **21**, note that **21** ionizes in the +2 charge state as the $2H^+$ ion and, less abundantly, as the H^+Na^+ ion; the enlarged region shows the m/z +2 peak. m/z = 794.79 corresponds to the +3 charge state of **21**, m/z = 596.35 corresponds to the +4 charge state of **21**, m/z = 477.28 corresponds to the +5 charge state of **21**, and m/z = 397.90 corresponds to the +6 charge state of **21**. The peptide is estimated to be >90% pure based on HPLC analysis. Note, m/z values referred to are for the most abundant isotope.

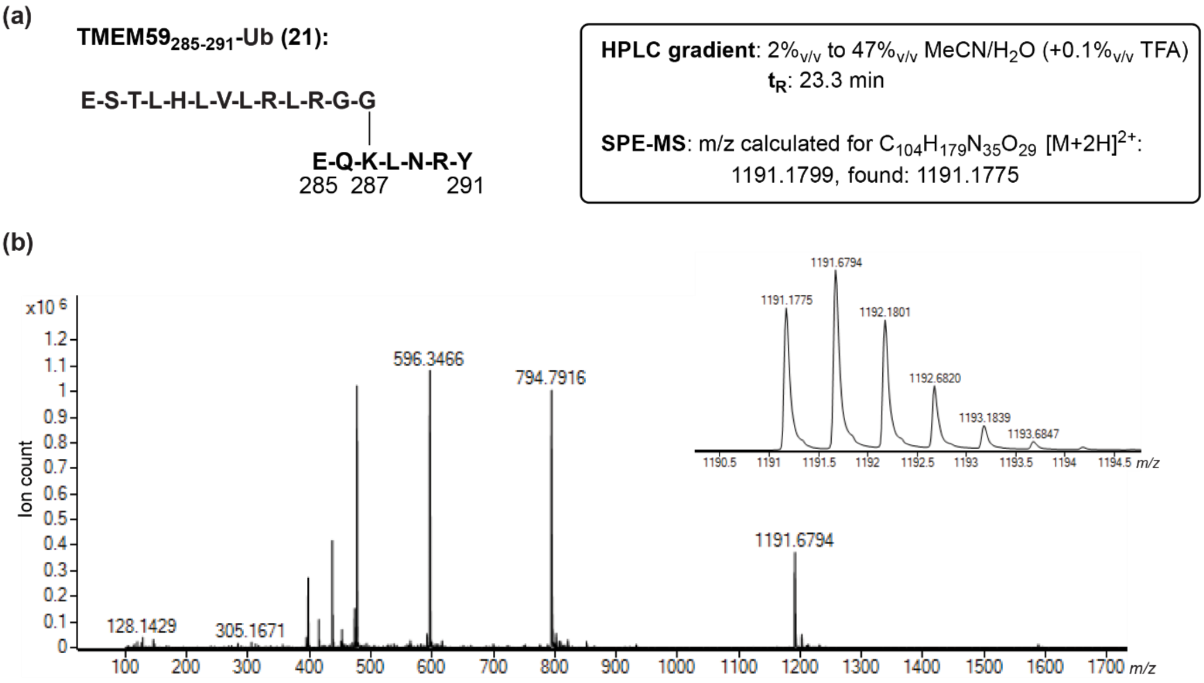

(**XIX**) (**a**) Sequence and purification characteristics of the K_{302}-branched $TMEM59_{298\text{-}305}$-Ub oligopeptide **22**; (**b**) mass spectrum (SPE-MS) of **22** (2.0 μM) in water. m/z = 1183.18 corresponds to the +2 charge state of **22**, note that **22** ionizes in the +2 charge state as the $2H^+$ ion and, less abundantly, as the H^+Na^+ and $2Na^+$ ions; the enlarged region shows the m/z +2 peak. m/z = 789.12 corresponds to the +3 charge state of **22**, m/z = 592.09 corresponds to the +4 charge state of **22**, m/z = 473.88 corresponds to the +5 charge state of **22**, and m/z = 395.06 corresponds to the +6 charge state of **22**. The peptide is estimated to be >90% pure based on HPLC analysis. Note, m/z values referred to are for the most abundant isotope.

(a)

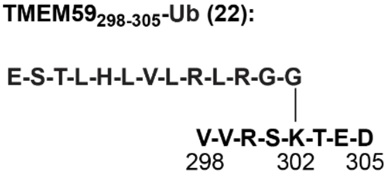

HPLC gradient: $2\%_{v/v}$ to $47\%_{v/v}$ MeCN/H_2O (+$0.1\%_{v/v}$ TFA)
t_R: 22.5 min

SPE-MS: m/z calculated for $C_{101}H_{180}N_{34}O_{31}$ $[M+2H]^{2+}$: 1182.6772, found:

(b)

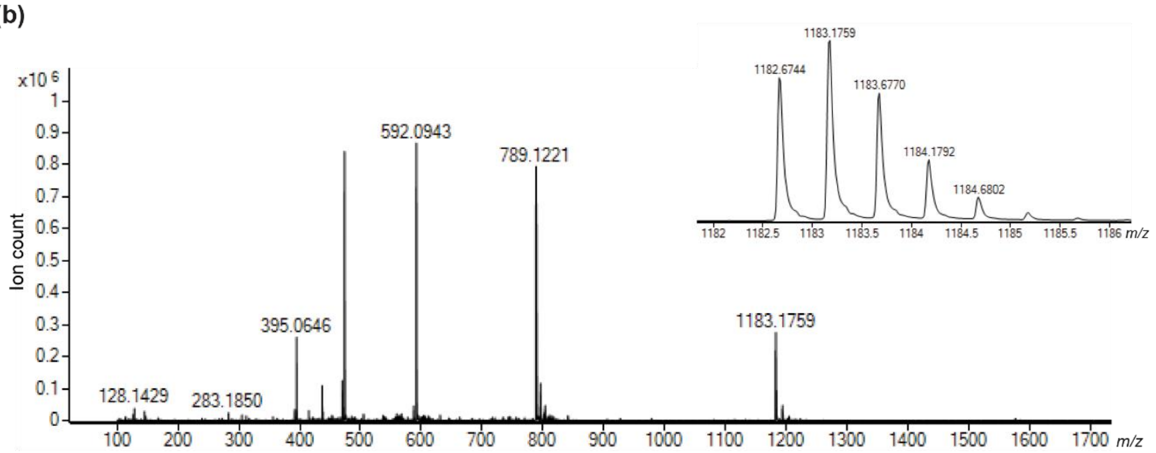

(**XX**) (**a**) Sequence and purification characteristics of the K_{338}-branched SARS-CoV-2_$N_{335\text{-}341}$-Ub oligopeptide **23**; (**b**) mass spectrum (SPE-MS) of **23** (2.0 μM) in water. m/z = 1082.13 corresponds to the +2 charge state of **23**, note that **23** ionizes in the +2 charge state as the $2H^+$ ion and, less abundantly, as the H^+Na^+ and $2Na^+$ ions; the enlarged region shows the m/z +2 peak. m/z = 721.75 corresponds to the +3 charge state of **23**, m/z = 541.57 corresponds to the +4 charge state of **23**, and m/z = 433.46 corresponds to the +5 charge state of **23**. The peptide is estimated to be >90% pure based on HPLC analysis. Note, m/z values referred to are for the most abundant isotope.

(a)

SARS-CoV-2_$N_{335\text{-}341}$-Ub (23):

E-S-T-L-H-L-V-L-R-L-R-G-G

G-A-I-K-L-D-D (branch at K)

335 338 341

HPLC gradient: $2\%_{v/v}$ to $47\%_{v/v}$ MeCN/H_2O (+$0.1\%_{v/v}$ TFA)

t_R: 24.8 min

SPE-MS: m/z calculated for $C_{94}H_{166}N_{30}O_{28}$ $[M+2H]^{2+}$: 1081.6239, found: 1081.6247

(b)

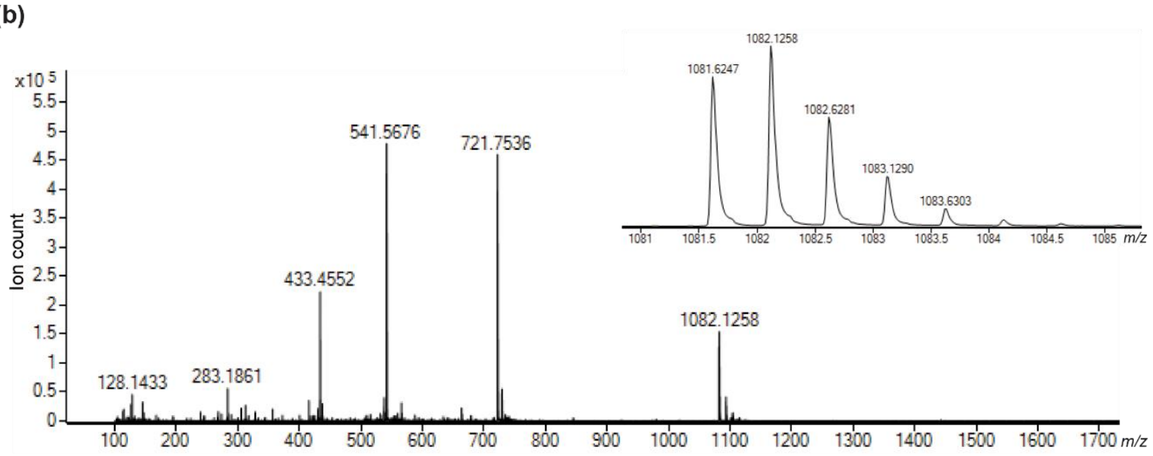

(**XXI**) (**a**) Sequence and purification characteristics of the Ub-derived N-terminally *N*-acetylated inert standard peptide Ac-ESTLHLVLRLRGG-NH_2 (**24**); (**b**) mass spectrum (SPE-MS) of **24** (2.0 µM) in water. m/z = 746.44 corresponds to the +2 charge state of **24**, note that **24** ionizes in the +2 charge state as the $2H^+$ ion and, less abundant, as the H^+Na^+ ion; the enlarged region shows the m/z +2 peak. m/z = 1491.87 corresponds to the +1 charge state of **24**, and m/z = 497.97 corresponds to the +3 charge state of **24**. The peptide is estimated to be >90% pure based on HPLC analysis. Note, m/z values referred to are for the most abundant isotope.

(a)

N-terminally *N*-acetylated
Ub-derived internal standard (24):

Ac-**E-S-T-L-H-L-V-L-R-L-R-G-G**

HPLC gradient: 2%$_{v/v}$ to 47%$_{v/v}$ MeCN/H_2O (+0.1%$_{v/v}$ TFA)
t_R: 26.3 min

SPE-MS: m/z calculated for $C_{65}H_{116}N_{22}O_{18}$ $[M+2H]^{2+}$: 746.4414, found: 746.4448

(b)

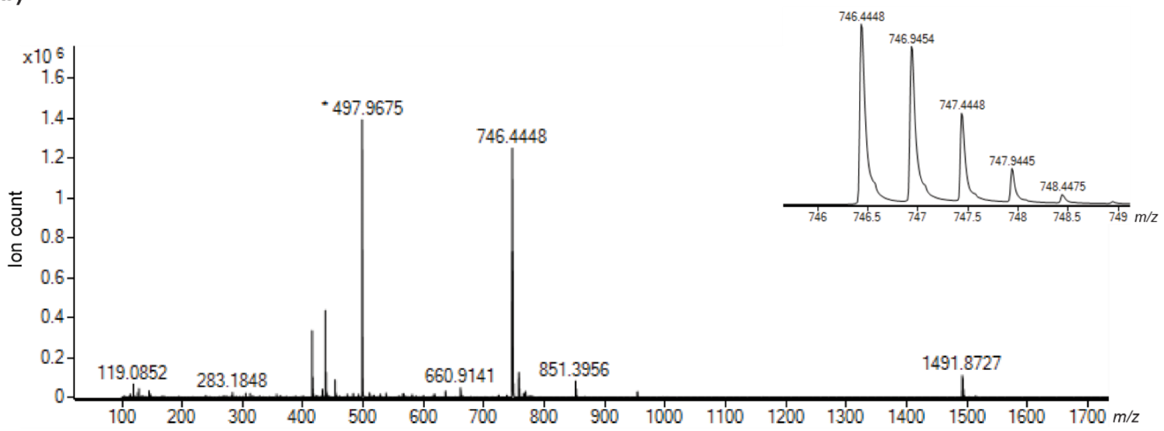

(**XXII**) (**a**) Sequence and purification characteristics of the K_{193}-branched $IRF3_{189\text{-}197}$-URM1-R oligopeptide **25**; (**b**) mass spectrum (SPE-MS) of **25** (2.0 µM) in water. m/z = 1228.21 corresponds to the +2 charge state of **25**, note that **25** ionizes in the +2 charge state as the $2H^+$ ion and, less abundantly, as the Na^+H^+ and $2Na^+$ ions; the enlarged region shows the m/z +2 peak. m/z = 819.15 corresponds to the +3 charge state of **25**, and m/z = 614.61 corresponds to the +4 charge state of **25**. The peptide is estimated to be >90% pure based on HPLC analysis. Note, m/z values referred to are for the most abundant isotope.

(a)

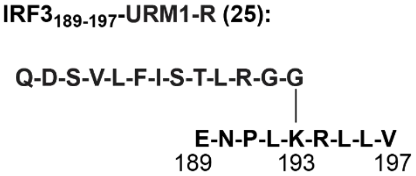

HPLC gradient: 2%$_{v/v}$ to 47%$_{v/v}$ MeCN/H_2O (+0.1%$_{v/v}$ TFA)
t_R: 31.8 min

SPE-MS: m/z calculated for $C_{110}H_{190}N_{32}O_{31}$ $[M+2H]^{2+}$: 1227.7132, found: 1227.7118

(b)

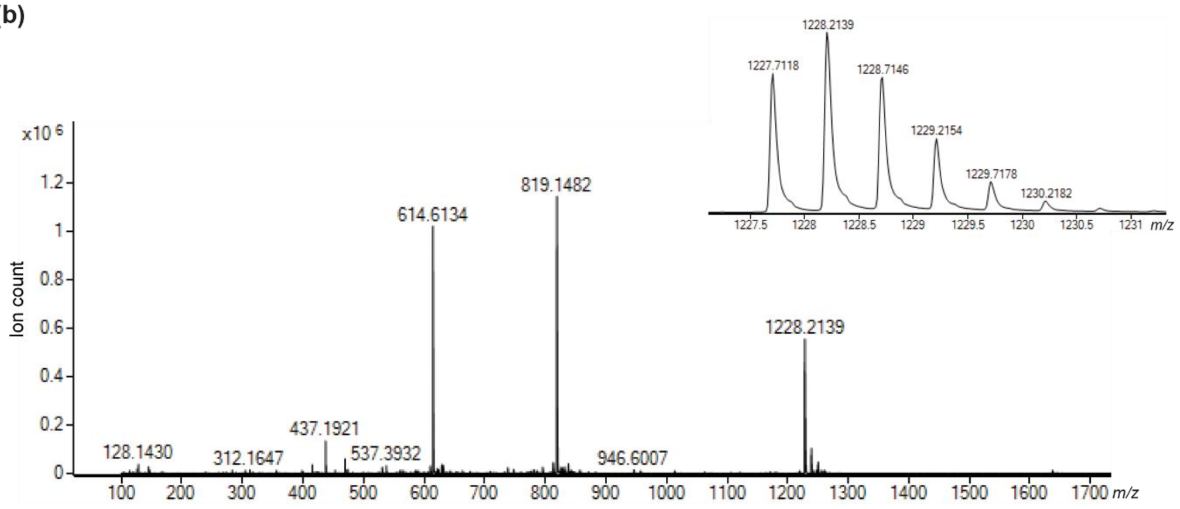

(**XXIII**) (**a**) Sequence and purification characteristics of the K_{338}-branched SARS-CoV-2_$N_{335\text{-}341}$-Ub_$L_{340}V_{341}$ oligopeptide **26**; (**b**) mass spectrum (SPE-MS) of **26** (2.0 µM) in water. m/z = 1073.17 corresponds to the +2 charge state of **26**, note that **26** ionizes in the +2 charge state as the $2H^+$ ion and, less abundantly, as the H^+Na^+ and $2Na^+$ ions; the enlarged region shows the m/z +2 peak. m/z = 715.79 corresponds to the +3 charge state of **26**, m/z = 537.09 corresponds to the +4 charge state of **26**, and m/z = 429.88 corresponds to the +5 charge state of **26**. The peptide is estimated to be >90% pure based on HPLC analysis. Note, m/z values referred to are for the most abundant isotope.

(a)

SARS-CoV-2_$N_{335\text{-}341}$-Ub_$L_{340}V_{341}$ (26):

E-S-T-L-H-L-V-L-R-L-R-G-G
|
G-A-I-K-L-L-V
335 338 341

HPLC gradient: $2\%_{v/v}$ to $47\%_{v/v}$ MeCN/H_2O (+$0.1\%_{v/v}$ TFA)
t_R: 29.7 min

SPE-MS: m/z calculated for $C_{97}H_{176}N_{30}O_{24}$ $[M+2H]^{2+}$: 1072.6732, found: 1072.6726

(b)

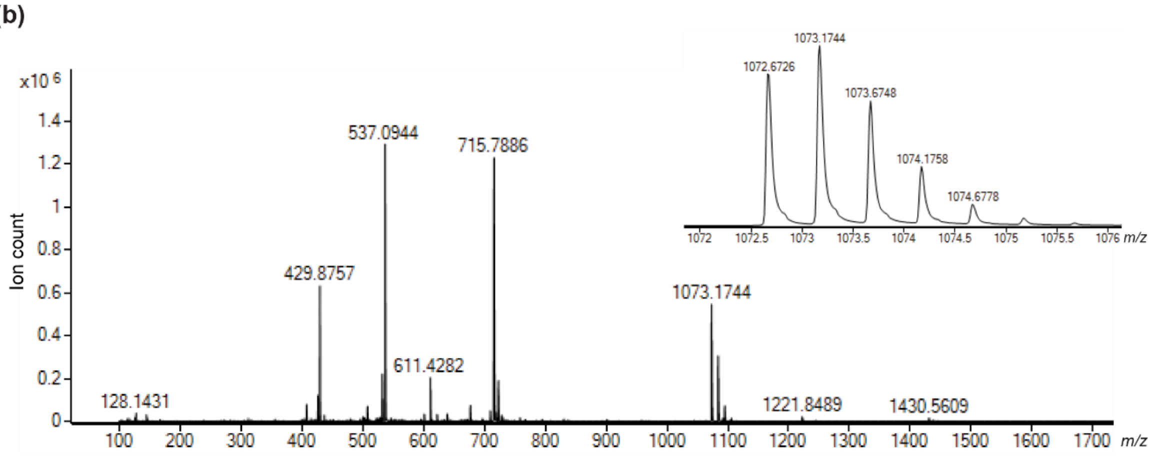

Supporting Figure S2. The N-terminally *N*-acetylated ISG15-derived oligopeptide 5 does not affect SARS-CoV-2 PL^{pro} catalysis and can be used as an internal standard. (**a**) The interferon stimulated gene 15 (ISG15)-derived N-terminally *N*-acetylated N-terminal product peptide (*i.e.*, Ac-LSTVFMNLRLRGG-NH$_2$, **5**; Supporting Figure S1) is not a substrate of isolated recombinant SARS-CoV-2 PL^{pro}; formation of the corresponding *N*-deacylated product (teal circles) was not observed by SPE-MS under assay conditions (SARS-CoV-2 PL^{pro} (0.2 μM) and **5** (2.0 μM) in 50 mM Tris, pH 8.0 at ambient temperature). Results are a mean of independent triplicates (n = 3; mean ± standard deviation, SD); (**b**) SPE-MS analysis reveals that isolated recombinant SARS-CoV-2 PL^{pro} catalyzes the formation of the ISG15-derived LSTVFMNLRLRGG oligopeptide from the K$_{193}$-branched IRF3$_{189-197}$-ISG15 oligopeptide **4** in similar rates in the absence (blue circles) and presence (orange boxes) of **5** (conditions: SARS-CoV-2 PL^{pro} (0.2 μM), **4** (2.0 μM), in the presence or absence of **5** (0.2 μM) in 50 mM Tris, pH 8.0 at ambient temperature); thus, the presence of the N-terminally *N*-acetylated N-terminal oligopeptide **5** in an assay mixture does not affect PL^{pro} catalysis. Results are a mean of independent triplicates (n = 3; mean ± SD).

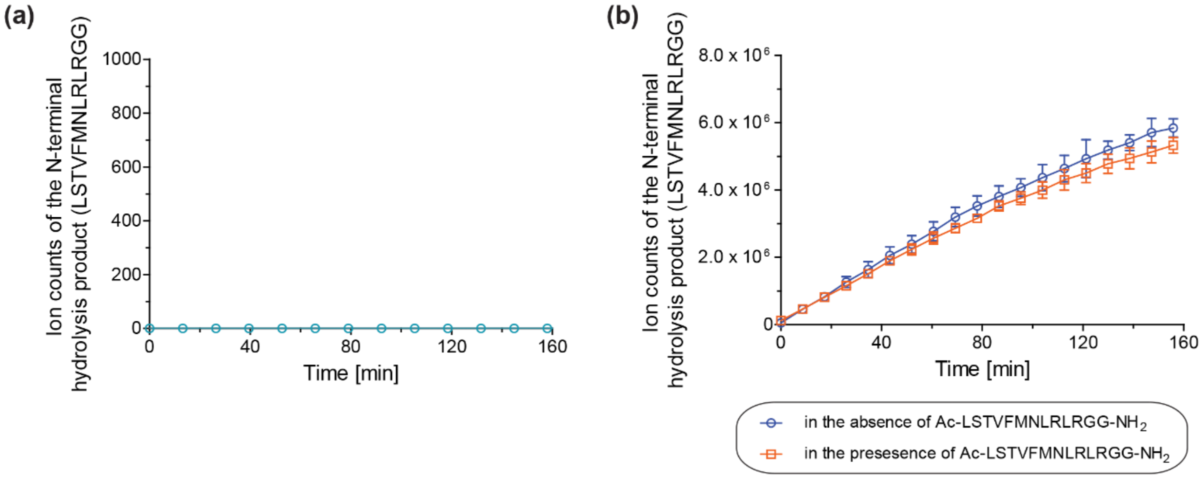

Supporting Figure S3. The N-terminally *N*-acetylated IRF3-derived oligopeptide 12 does not affect SARS-CoV-2 PL^{pro} catalysis and can be used as an internal standard. (**a**) The interferon regulatory factor 3 (IRF3)-derived N-terminally *N*-acetylated C-terminal product peptide (*i.e.*, Ac-ENPLKRLLV-NH_2, **12**; Supporting Figure S1) is not a substrate of isolated recombinant SARS-CoV-2 PL^{pro}; formation of the corresponding *N*-deacylated product (teal circles) was not observed by SPE-MS under assay conditions (SARS-CoV-2 PL^{pro} (0.2 µM) and **12** (2.0 µM) in 50 mM Tris, pH 8.0 at ambient temperature). Results are a mean of independent triplicates (n = 3; mean ± SD); (**b**) SPE-MS analysis reveals that isolated recombinant SARS-CoV-2 PL^{pro} catalyzes the formation of the ISG15-derived ENPLKRLLV oligopeptide from the K_{193}-branched $IRF3_{189-197}$-ISG15 oligopeptide **4** in similar rates in the absence (blue circles) and presence (orange boxes) of **12** (conditions: SARS-CoV-2 PL^{pro} (0.2 µM), **4** (2.0 µM), in the presence or absence of **12** (0.2 µM) in 50 mM Tris, pH 8.0 at ambient temperature); thus, the presence of the N-terminally *N*-acetylated C-terminal oligopeptide **12** in an assay mixture does not affect PL^{pro} catalysis. Results are a mean of independent triplicates (n = 3; mean ± SD).

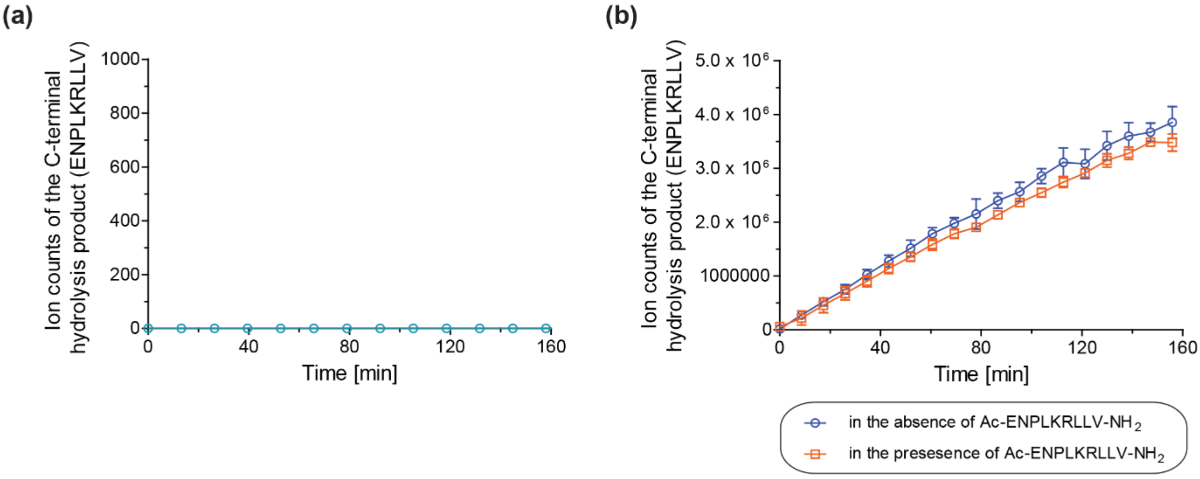

Supporting Figure S4. The N-terminally *N*-acetylated Ub-derived oligopeptide 24 does not affect SARS-CoV-2 PL^{pro} catalysis and can be used as an internal standard. (**a**) The ubiquitin (Ub)-derived N-terminally *N*-acetylated N-terminal product peptide (*i.e.*, Ac-ESTLHLVLRLRGG-NH_2, **24**; Supporting Figure S1) is not a substrate of isolated recombinant SARS-CoV-2 PL^{pro}; formation of the corresponding *N*-deacylated product (teal circles) was not observed by SPE-MS under assay conditions (SARS-CoV-2 PL^{pro} (0.2 µM) and **24** (2.0 µM) in 50 mM Tris, pH 8.0 at ambient temperature). Results are a mean of independent triplicates (n = 3; mean ± SD); (**b**) SPE-MS analysis reveals that isolated recombinant SARS-CoV-2 PL^{pro} catalyzes the formation of the Ub-derived ESTLHLVLRLRGG oligopeptide from the K_{193}-branched $IRF3_{189\text{-}197}$-Ub oligopeptide **15** in similar rates in the absence (black diamonds) and presence (orange boxes) of **24** (conditions: SARS-CoV-2 PL^{pro} (0.2 µM), **15** (2.0 µM), in the presence or absence of **24** (0.2 µM) in 50 mM Tris, pH 8.0 at ambient temperature); thus, the presence of the N-terminally *N*-acetylated N-terminal oligopeptide **24** in an assay mixture does not affect PL^{pro} catalysis. Results are a mean of independent triplicates (n = 3; mean ± SD).

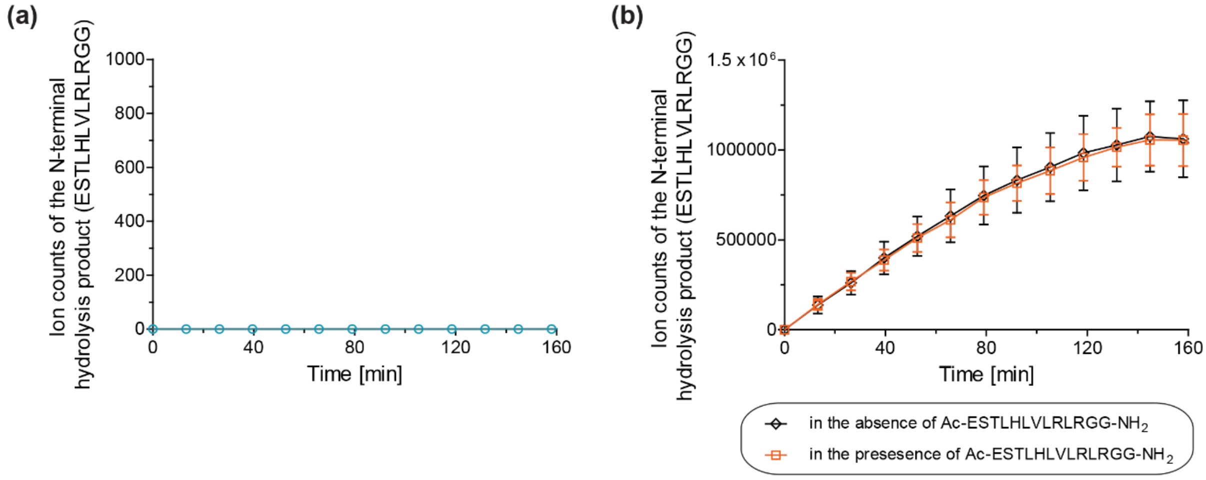

Supporting Figure S5. AutoDock CrankPep (ADCP)[1]-predicted conformations of the pp1a/1ab-derived nsp2/3$_{808-827}$ oligopeptide **2** (VTNNTFTLKGG|APTKVTFGD, the scissile amide bond is indicated with '|')[2] in complex with SARS-CoV-2 PL^{pro} (PDB ID: 6WX4[3]). The 1st and 48th ranked poses are in cyan and yellow, respectively. The N- and C-termini of **2** are labelled, the P1 scissile amide carbon atom is a green sphere, and the P4′ lysine of the 48th ranked pose is in orange; the PL^{pro} active site C111 is in magenta. These poses provided a basis for modelling the branched peptide sequences.

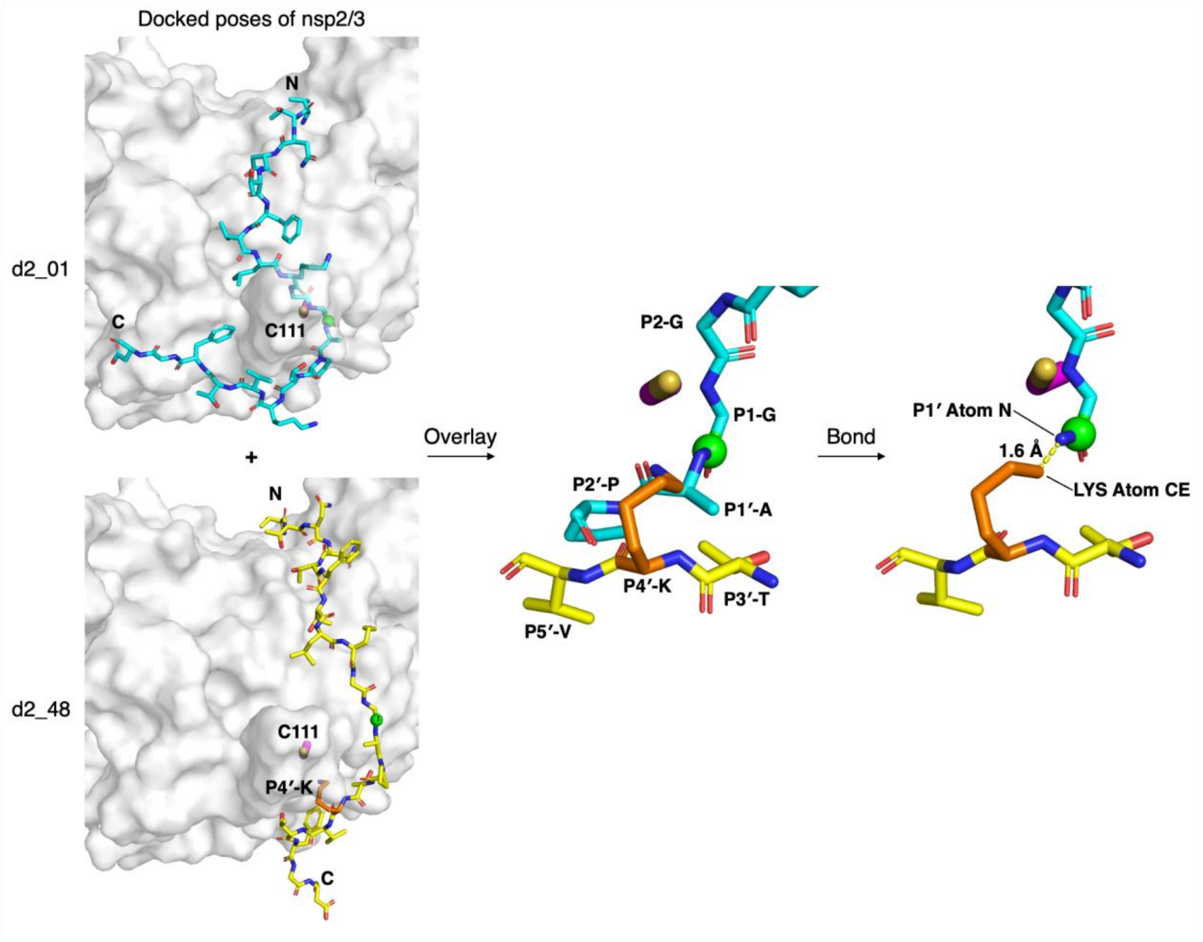

Supporting Figure S6. View of the modelled active site of the PL^{pro}:IRF3$_{189-197}$-ISG15 (**4**) complex. The N^{ε}-lysine amide linkage was constructed as described in the Experimental section.

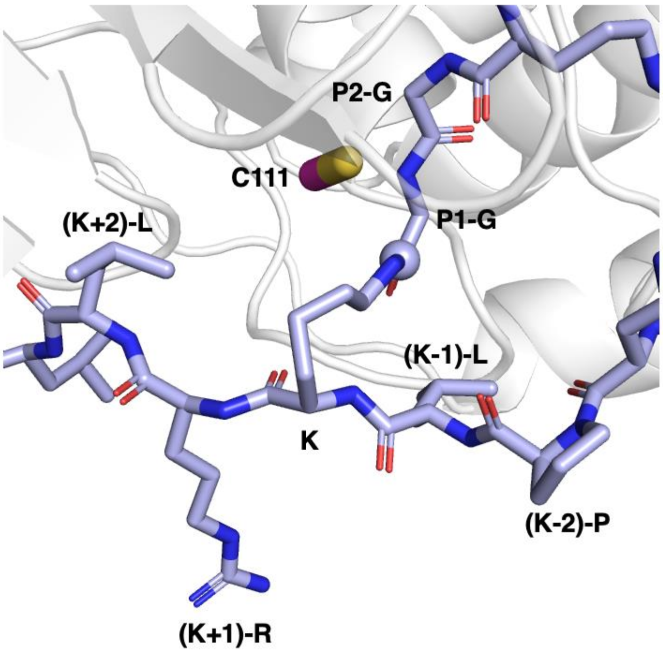

Supporting Figure S7. View of the modelled PL^{pro} complexes with the N^{ε}-lysine-branched oligopeptides IRF3$_{189-197}$-(ISG15/Ub/NEDD8/URM1/SUMO1) and (TMEM59$_{285-291}$/TMEM59$_{298-305}$/SARS-CoV-2_N$_{335-341}$)-Ub. The N-terminus of the UBL-derived fragment and the N- and C-termini of the S′ binding fragment are labelled N, N′, and C′, respectively. The P1 scissile amide carbon atoms are shown as a sphere, and C111 is in magenta. For molecular dynamics (MD) simulations of the URM1 derived peptide, both the neutral (^{N}) and positive charged ($^{+}$) states of the P3 histidine imidazole group were considered.

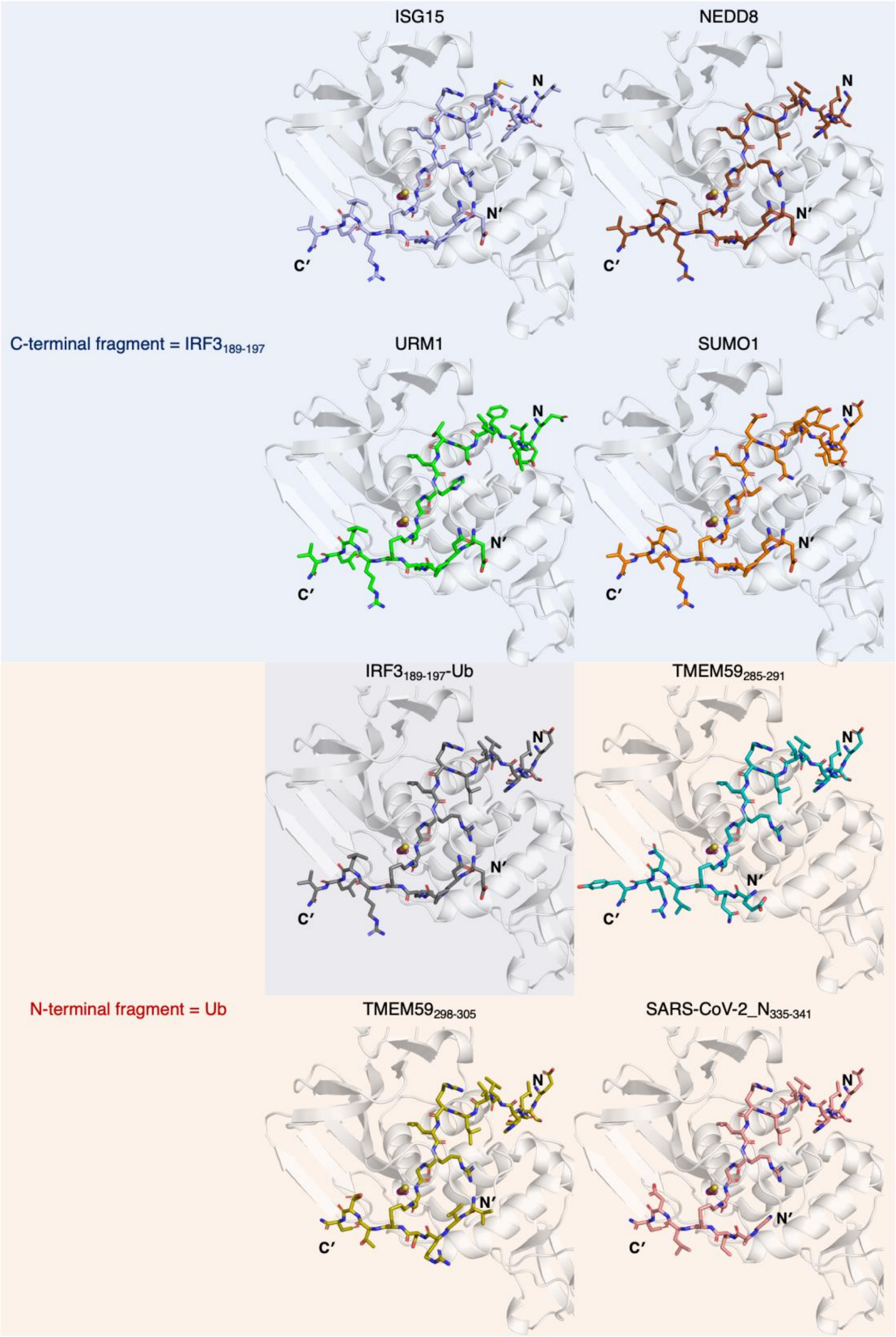

Supporting Figure S8. Root mean square deviation (RMSD) of PL^{pro} backbone atoms (N, C^{α}, C) in the combined 3 × 200 ns MD trajectories (fitted based on the PL^{pro} backbone), relative to the PL^{pro} crystal structure (PDB ID: 6WX4[3]).

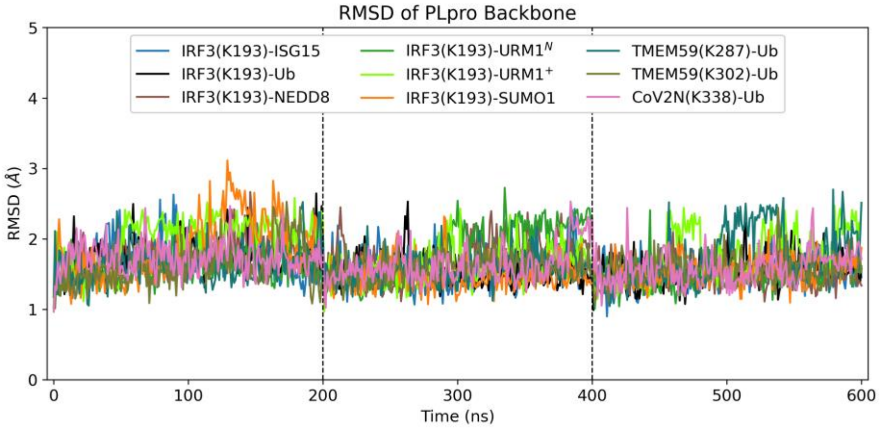

Supporting Figure S9. RMSD of peptide backbone atoms (N, C^{α}, C in both fragments) in the combined 3 × 200 ns MD trajectories (fitted based on the PL^{pro} backbone), relative to the starting peptide poses.

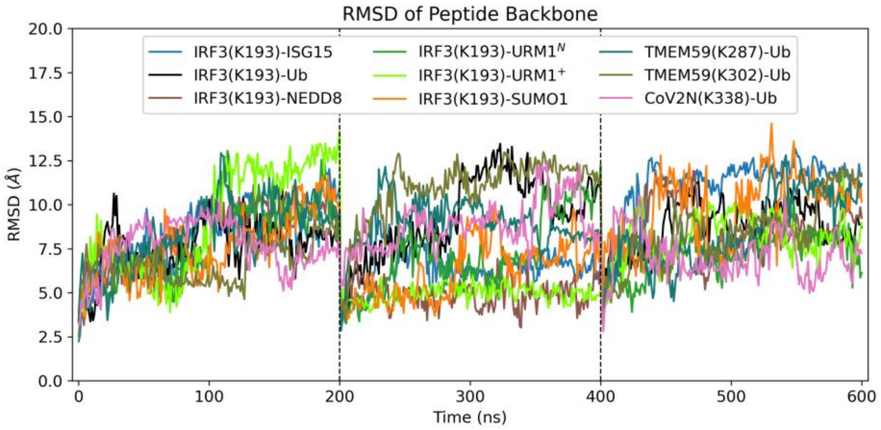

Supporting Figure S10. RMSD of peptide non-hydrogen atoms in the combined 3 × 200 ns MD trajectories (fitted based on the PL^{pro} backbone), relative to the starting peptide poses.

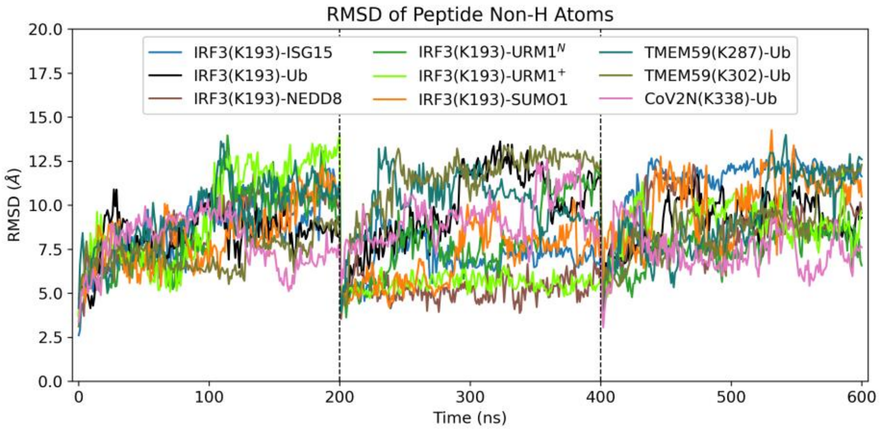

Supporting Figure S11. Cluster membership of frames extracted every ns from the combined 3 × 200 ns MD (fitted using PL^{pro} backbone) of each PL^{pro}:oligopeptide complex, with clustering performed using a 3 Å RMSD cut-off on the peptide backbone atoms using gmx cluster (gromos algorithm)[4,5]. Only the 5 clusters with the highest occupancy percentages are shown. Out of 601 frames, the number of (multimembered) clusters obtained for peptides $IRF3_{189-197}$-(ISG15/Ub/NEDD8/$URM1^{N}$/$URM1^{+}$/SUMO1) and ($TMEM59_{285-291}$/$TMEM59_{298-305}$/SARS-CoV-2_$N_{335-341}$)-Ub are 128 (57), 181 (78), 106 (58), 150 (63), 160 (63), 159 (74), 138 (76), 104 (61), and 123 (62), respectively.

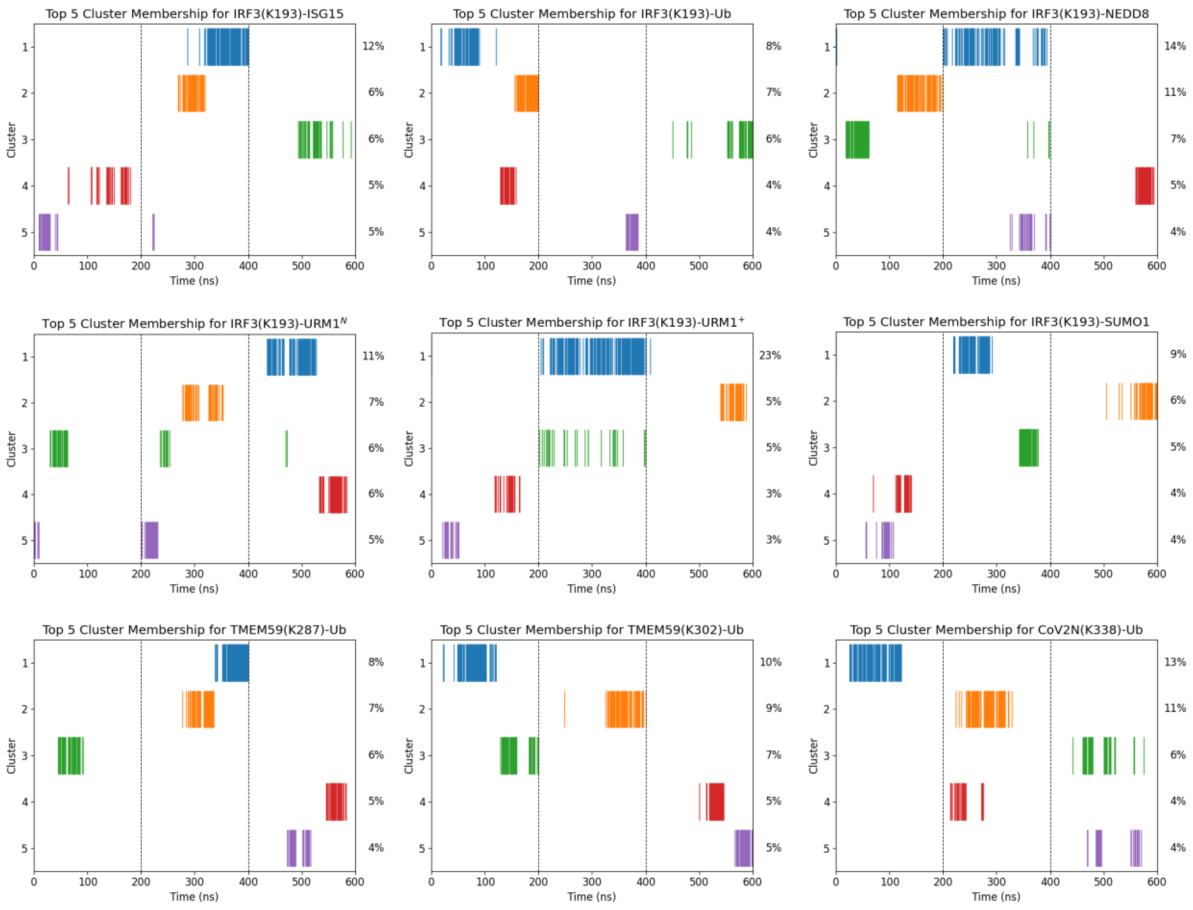

Supporting Figure S12. View of representative oligopeptide conformers from the PL^{pro}:IRF3$_{189\text{-}197}$-UBL (*i.e.*, **4**, **15**, **16**, **17**, and **18**) complexes of the three most populated clusters, obtained from the combined 3 × 200 ns MD of each of the PL^{pro} complexes. The PL^{pro} structure is from the most populated cluster, C111 is in magenta. The peptide backbones are shown in uniform colors with darker colors corresponding to more populated clusters (P1 scissile amide carbon atoms are shown as a sphere), with the N-terminus of the UBL fragment, and the N- and C-termini of the IRF3$_{189\text{-}197}$ fragment shown as black, blue, and red spheres, respectively.

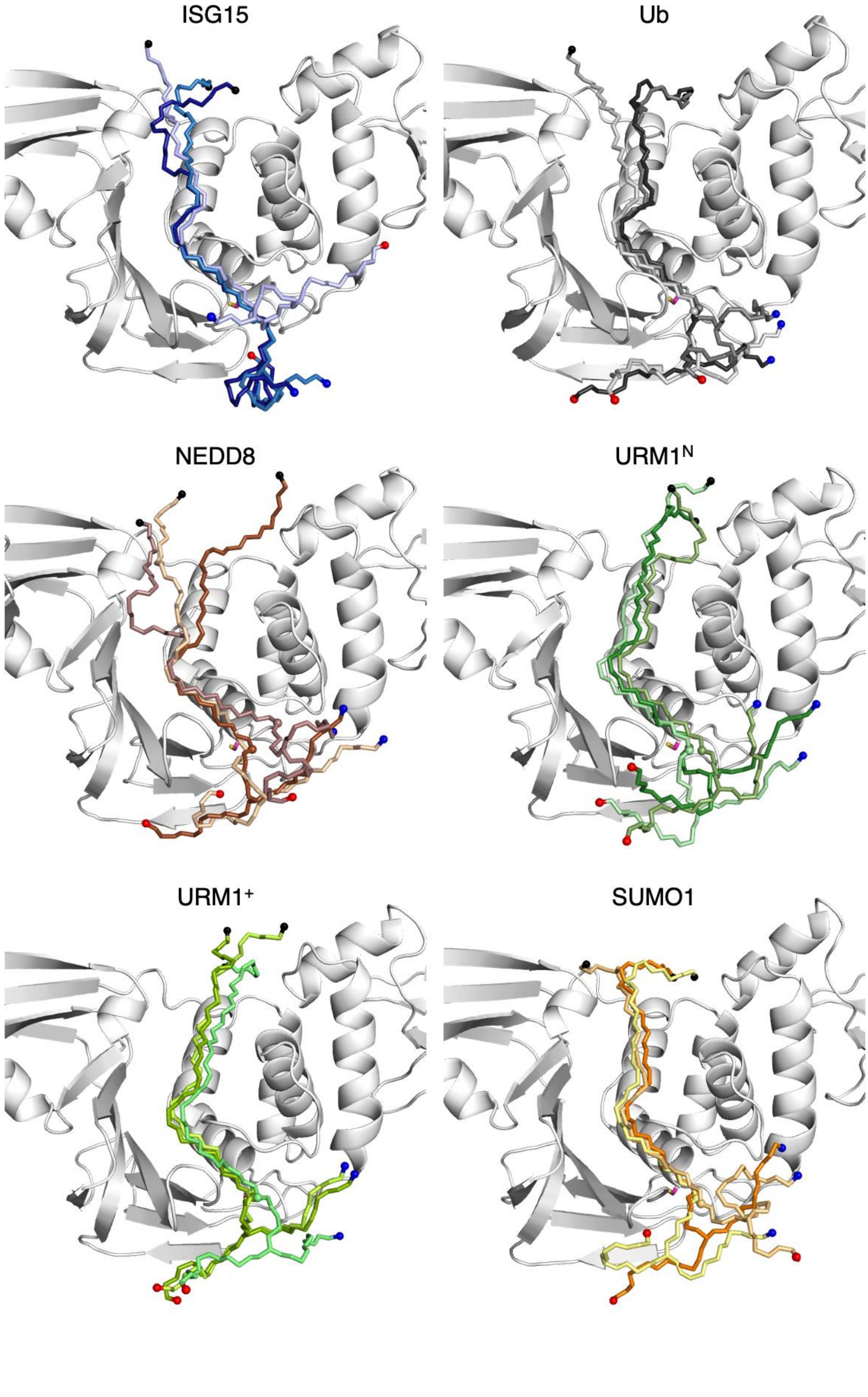

Supporting Figure S13. View of representative oligopeptide conformers from the PL^{pro}:oligopeptide-Ub (*i.e.*, **15**, **21**, **22**, and **23**) complexes of the three most populated clusters, obtained from the combined 3 × 200 ns MD of each of the PL^{pro} complexes. The PL^{pro} structure is from the most populated cluster, C111 is in magenta. The peptide backbones are shown in uniform colors with darker colors corresponding to more populated clusters (P1 scissile amide carbon atoms are shown as a sphere), with the N-terminus of the UBL fragment, and the N- and C-termini of the IRF3$_{189-197}$ fragment shown as black, blue, and red spheres, respectively.

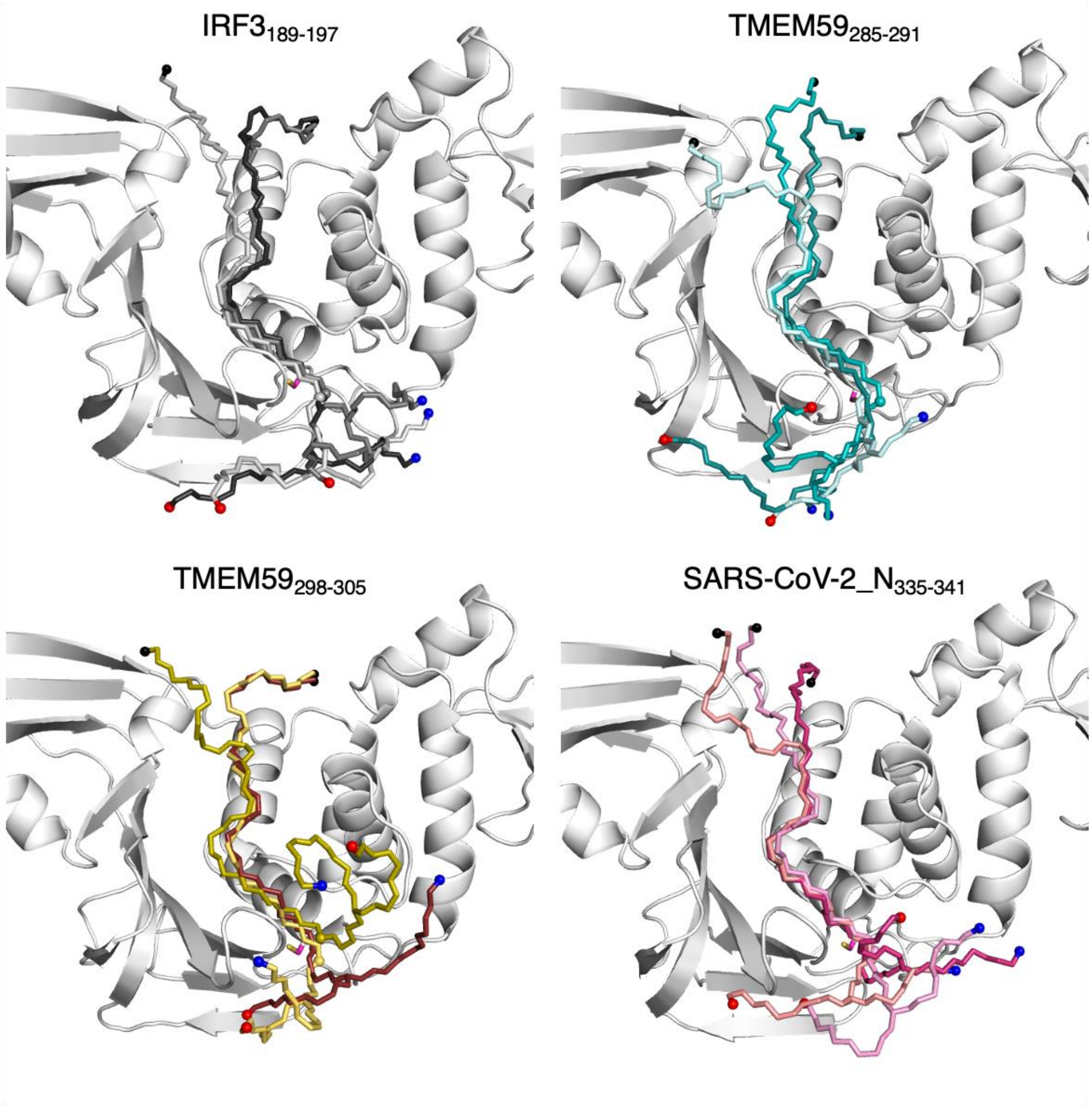

Supporting Figure S14. Root mean square fluctuation (RMSF) for each residue backbone atoms in the UBL fragment, in the combined 3 × 200 ns MD (fitted based on the PL^{pro} backbone).

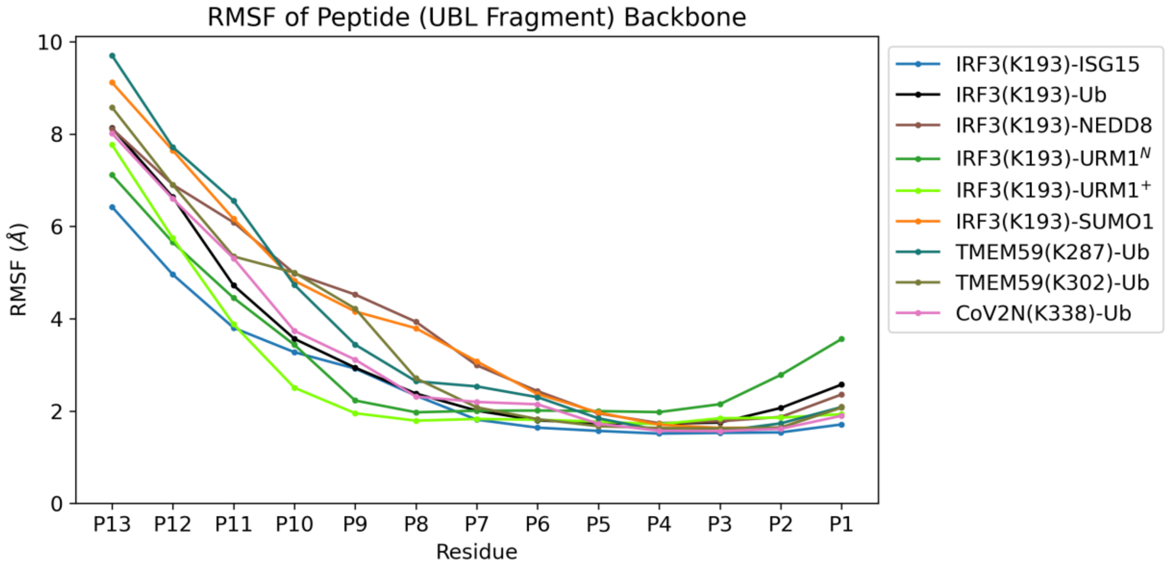

Supporting Figure S15. RMSF of peptide non-hydrogen atoms in the UBL fragment, in the combined 3 × 200 ns MD (fitted based on the PL^{pro} backbone).

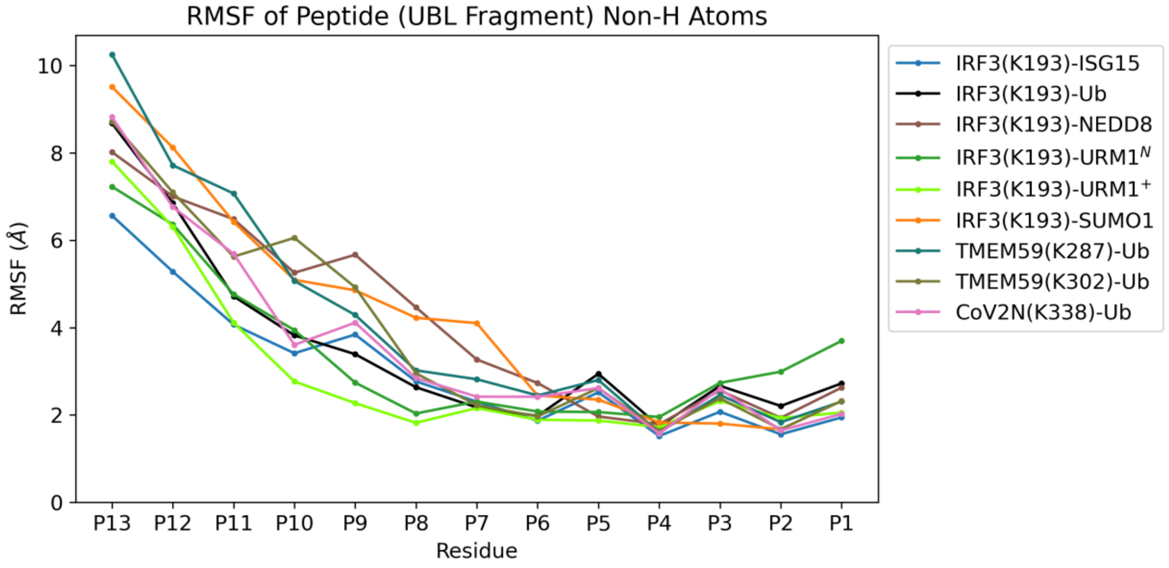

Supporting Figure S16. RMSF for each residue backbone atoms of the oligopeptide fragment binding to the PL^{pro} S′ sites, in the combined 3 × 200 ns MD (fitted based on the PL^{pro} backbone). Note that the C-terminal NH_2 group is treated as a separate residue.

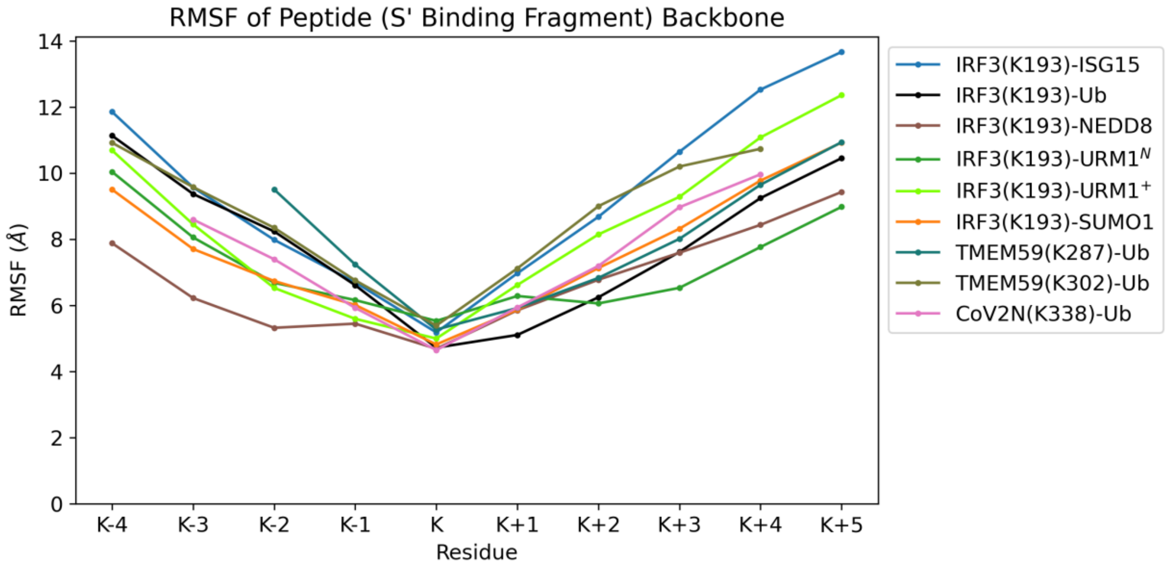

Supporting Figure S17. RMSF of peptide non-hydrogen atoms of the oligopeptide fragment binding to the PL^{pro} S′ sites, in the combined 3 × 200 ns MD (fitted based on the PL^{pro} backbone). Note that the C-terminal NH_2 group is treated as a separate residue.

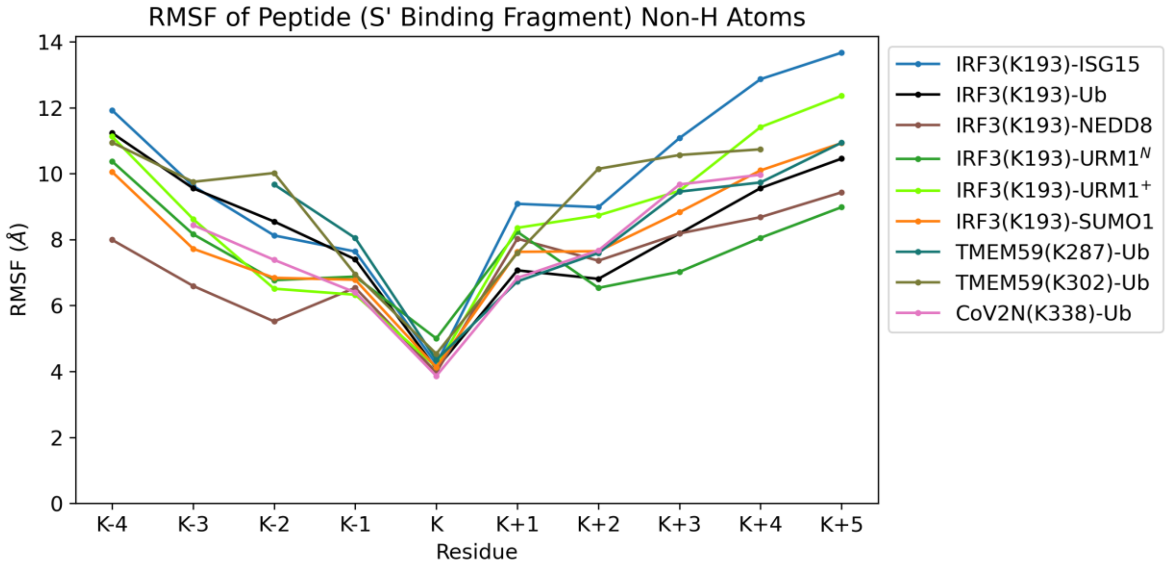

Supporting Figure S18. Conserved hydrogen bonding interactions in the P5-P1′ region and their percentage occurrences (occurrence ≥ 25% for at least five out of nine peptides), observed in 3 × 200 ns MD. Frames were analyzed every ns.

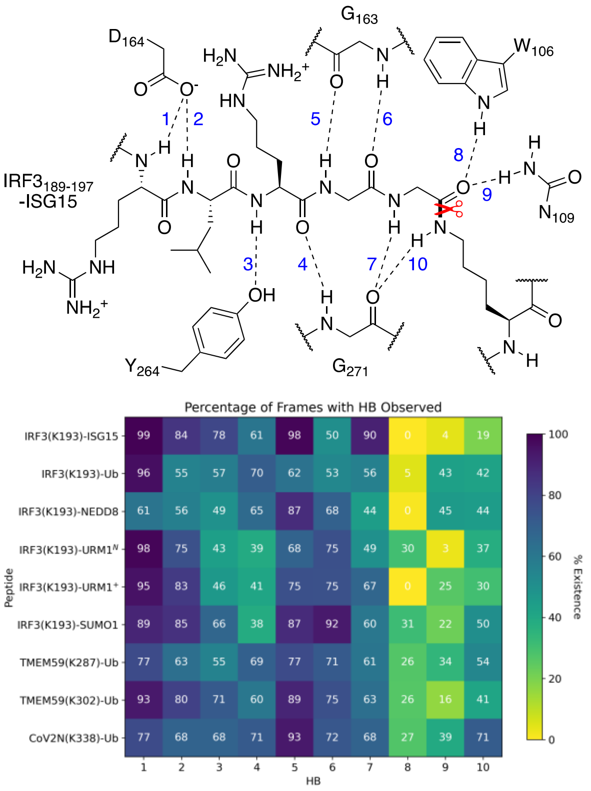

Supporting Figure S19. Event plots showing the occurrence of the conserved hydrogen bonding interactions in 3 × 200 ns MD.

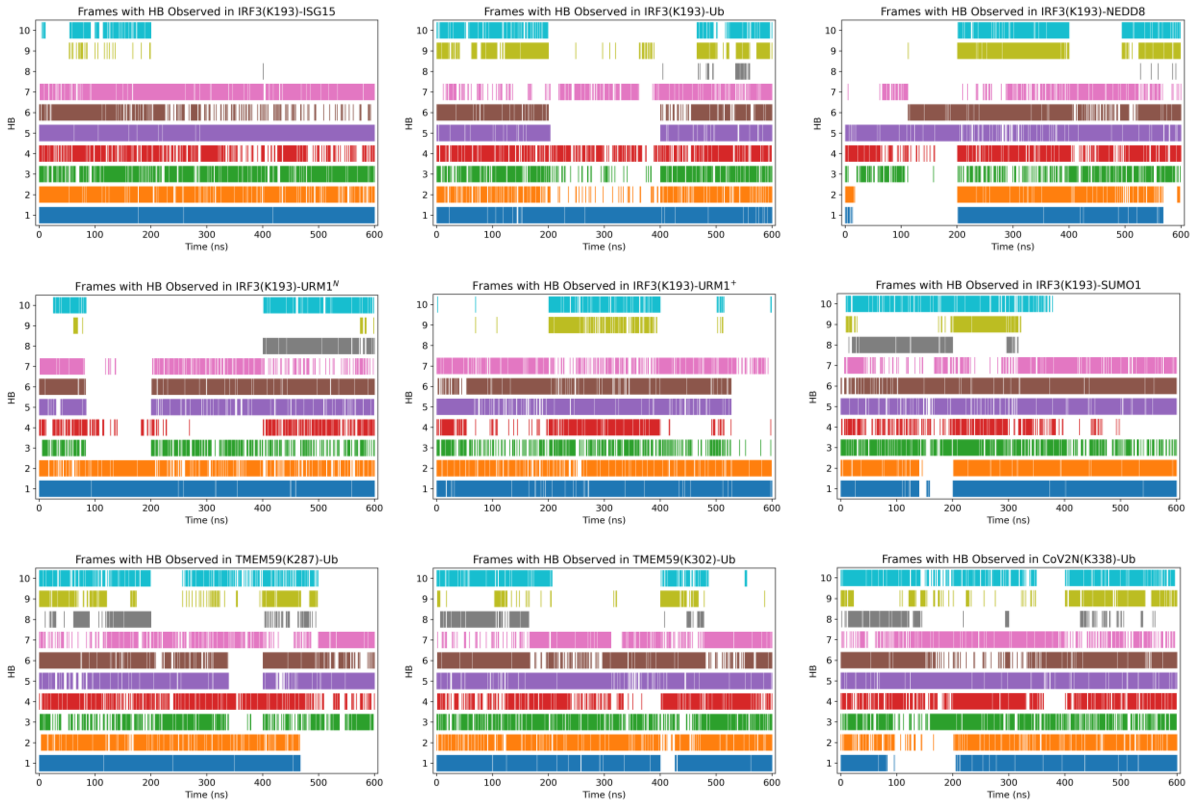

Supporting Figure S20. Contribution of each peptide residue to the PL^{pro}:oligopeptide molecular mechanics/generalized Born surface area (MM/GBSA) binding energy, from complex structures prior to MD and obtained from MD with frames being analyzed every 5 ns.

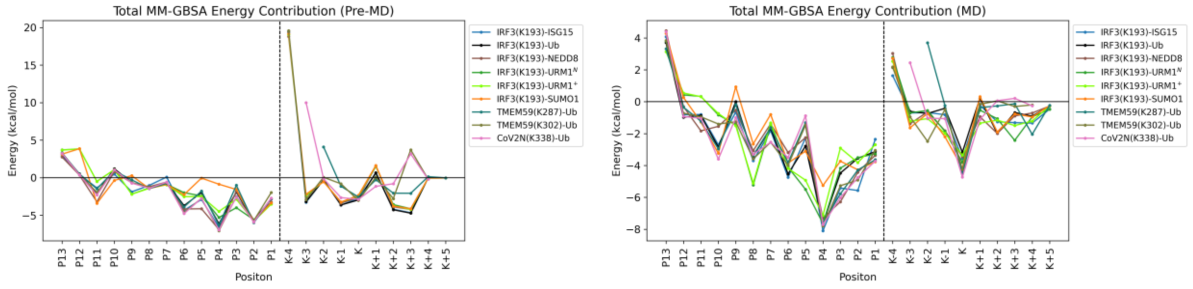

Supporting Figure S21. Contribution of the sidechain of every peptide residue to the PL^{pro}:oligopeptide MM/GBSA binding energy, from complex structures prior to MD and obtained from MD with frames being analyzed every 5 ns.

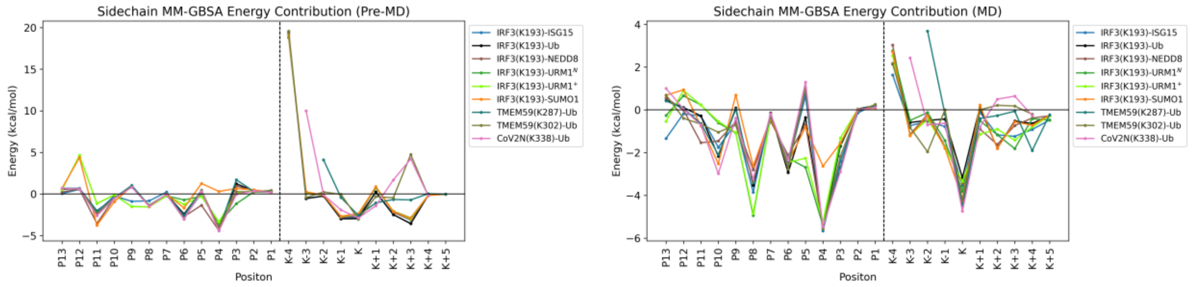

Supporting Figure S22. Contribution of the backbone of every peptide residue to the PL^{pro}:oligopeptide MM/GBSA binding energy, from complex structures prior to MD and obtained from MD with frames being analyzed every 5 ns.

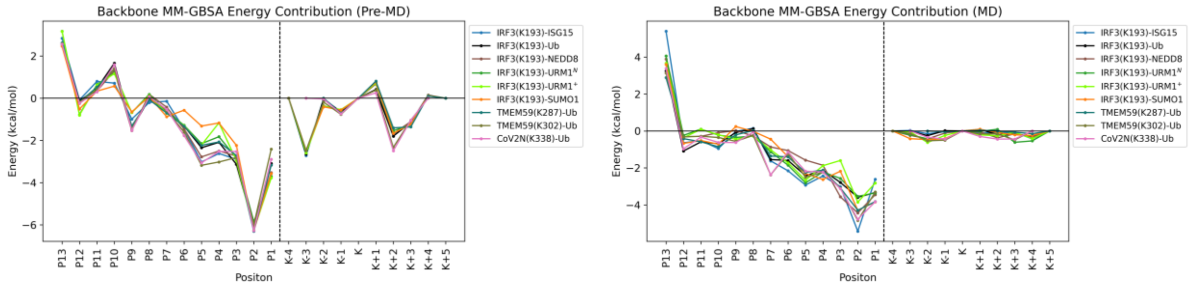

Supporting Figure S23. Contribution of the UBL-derived peptide fragment and the peptide fragment binding to the S′ sites to the PL^{pro}:oligopeptide MM/GBSA binding energy, from complex structures prior to MD and obtained from MD with frames being analyzed every 5 ns.

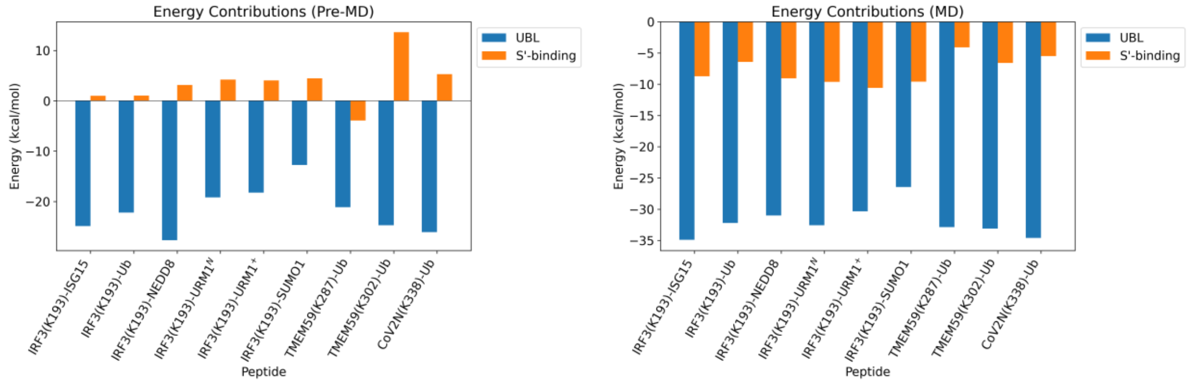

Supporting Figure S24. Contribution of the UBL-derived peptide fragment and the peptide fragment binding to the S′ sites (split into the individual contributions by the N^{ε}-branched lysine residue, and by the residues N- and C-terminal of this N^{ε}-branched lysine residue) to the PL^{pro}:oligopeptide MM/GBSA binding energy, from complex structures prior to MD and obtained from MD with frames being analyzed every 5 ns.

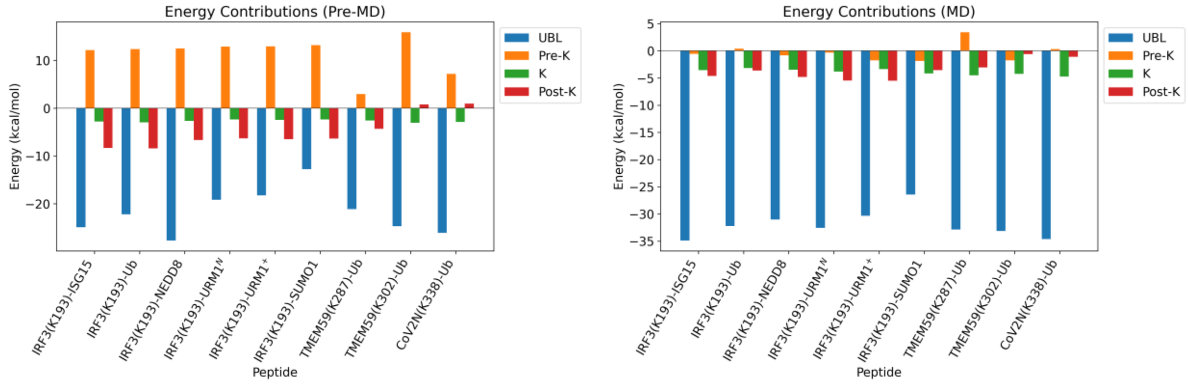

Supporting Figure S25. RMSF of the backbone atoms of PL^{pro} residues in the PL^{pro}:oligopeptide complexes in the combined 3 × 200 ns MD (left), and (right) focusing around the Y268-containing BL2 region.

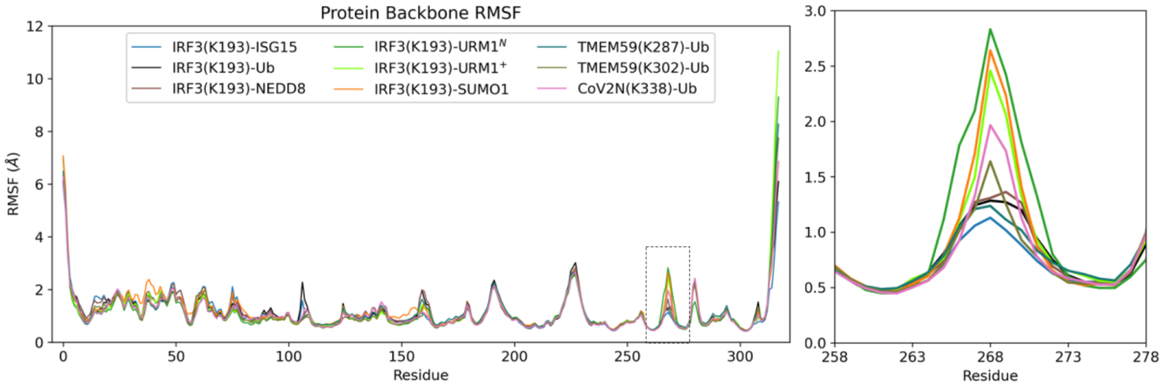

Supporting Figure S26. Views of the PL^{pro}:oligopeptide complexes representative of the most populated cluster (Supporting Figure S11) with PL^{pro} residues colored based on MD-derived backbone B-factors, when complexed with IRF3$_{189-197}$-(NEDD8/URM1^{N}/URM1^{+}) and (TMEM59$_{285-291}$/TMEM59$_{298-305}$)-Ub. BL2 (Gly266-Gly271) is shown as a thickened loop, with Y268 C$^{\alpha}$ shown as a sphere. The N-terminus of the UBL-derived fragment and the N- and C-termini of the S′ binding fragment are labelled N, N′, and C′, respectively.

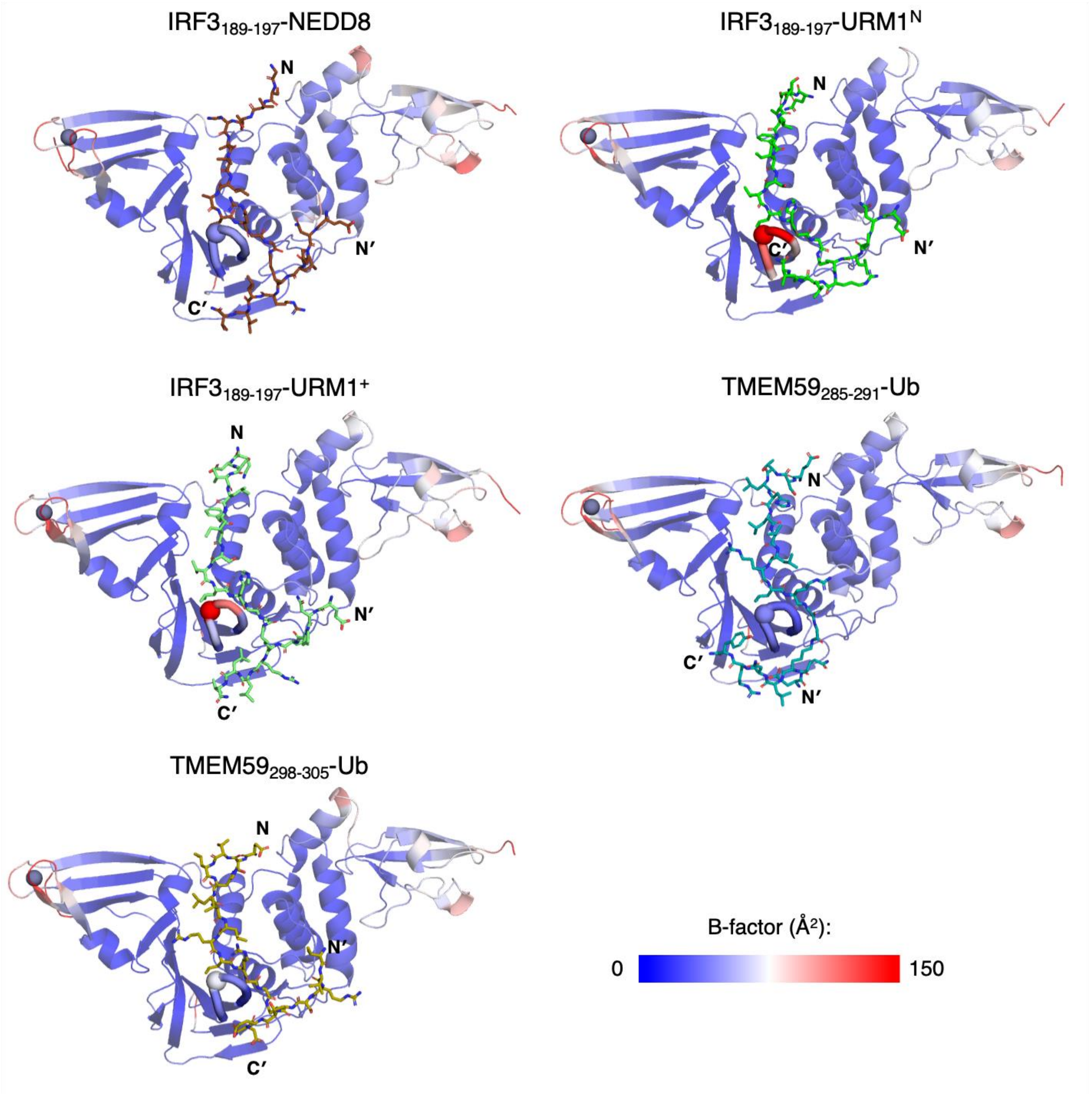

Supporting Figure S27. The distribution of the PL^{pro} P248–Y268 C$^{\alpha}$–C$^{\alpha}$ distance over combined 3 × 200 ns MD for each PL^{pro}:oligopeptide complex.

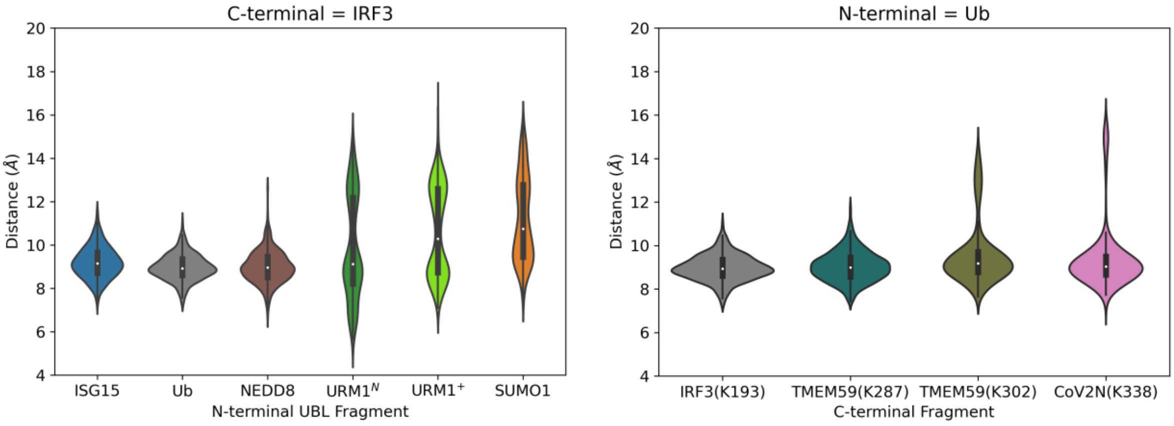

Supporting Figure S28. The evolution of the PL^{pro} P248–Y268 C$^{\alpha}$–C$^{\alpha}$ distance over combined 3 × 200 ns MD for each PL^{pro}:oligopeptide complex.

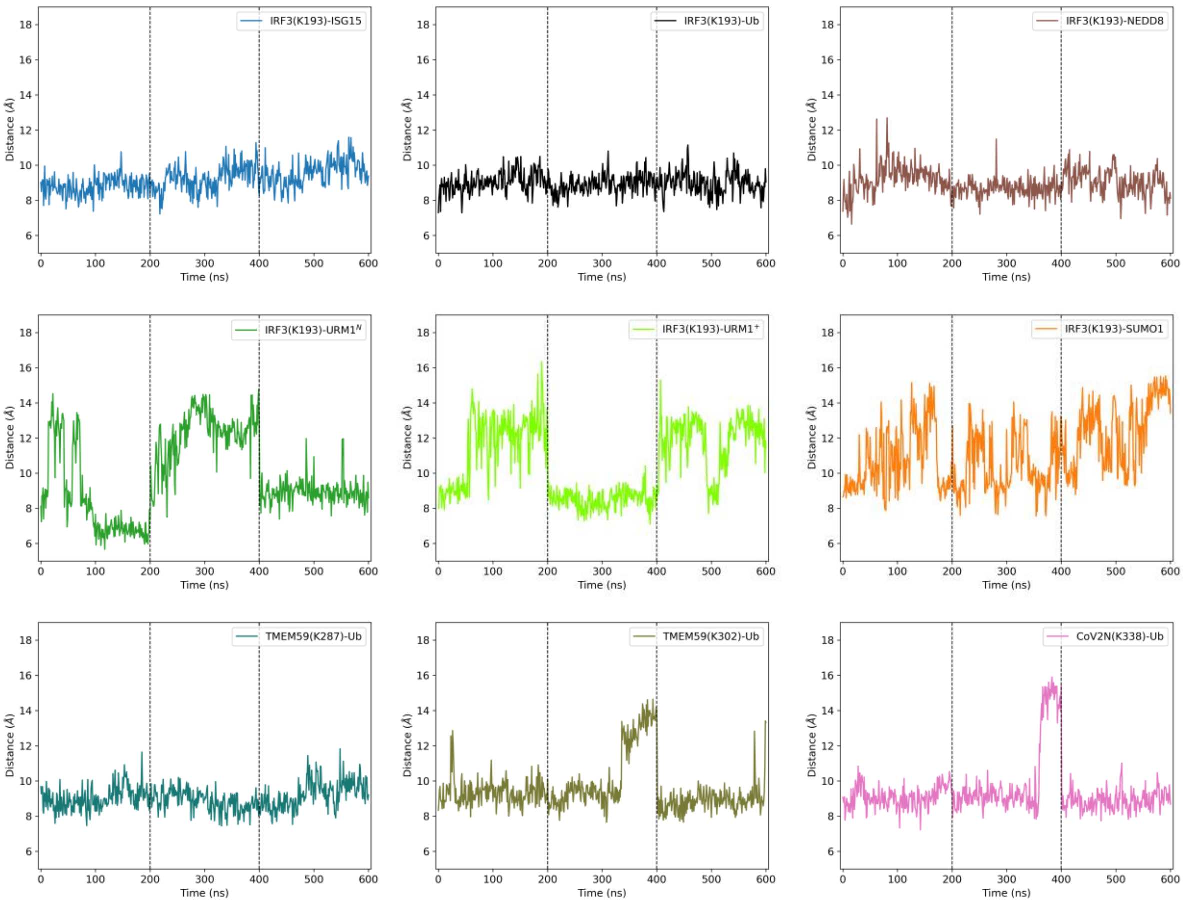

Supporting Figure S29. Pairwise contribution between Y268 and every peptide residue to the PL^{pro}:oligopeptide MM/GBSA binding energy, from complex structures prior to MD and obtained from MD with frames being analyzed every 5 ns.

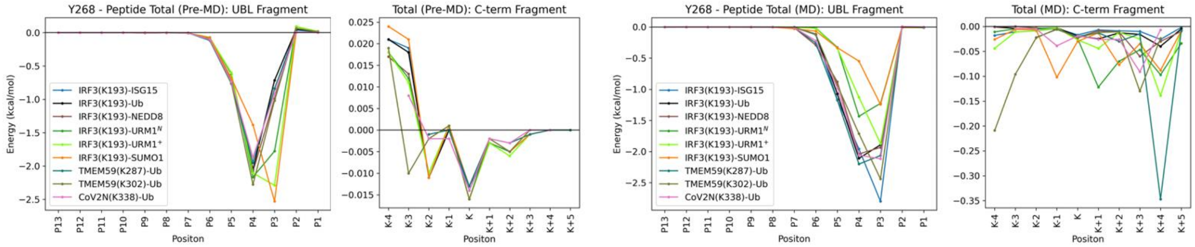

Supporting Figure S30. Pairwise contribution between Y268 and the sidechain of every peptide residue to the PL^{pro}:oligopeptide MM/GBSA binding energy, from complex structures prior to MD and obtained from MD with frames being analyzed every 5 ns.

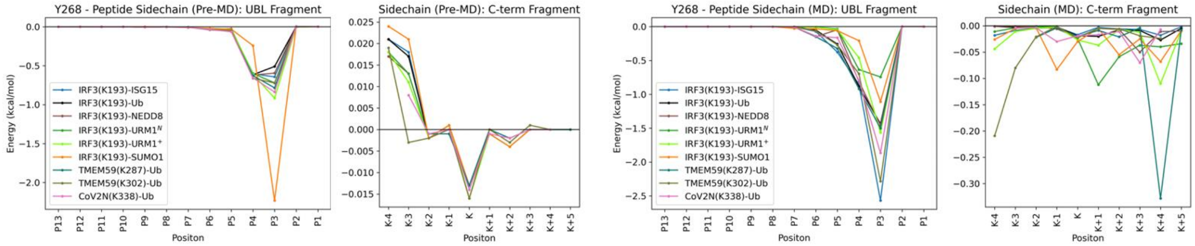

Supporting Figure S31. Pairwise contribution between Y268 and the backbone of every peptide residue to the PL^{pro}:oligopeptide MM/GBSA binding energy, from complex structures prior to MD and obtained from MD with frames being analyzed every 5 ns.

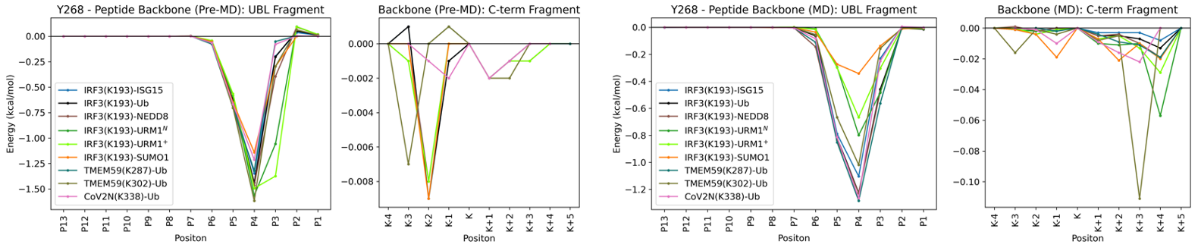

Supporting Figure S32. The structure of *N*-butyl acetamide was used as a model for parametrizing the N^{ε}-branched lysine residue (LYC). The mol2 file displays the determined AMBER atom types[6] and RESP charges[7,8].

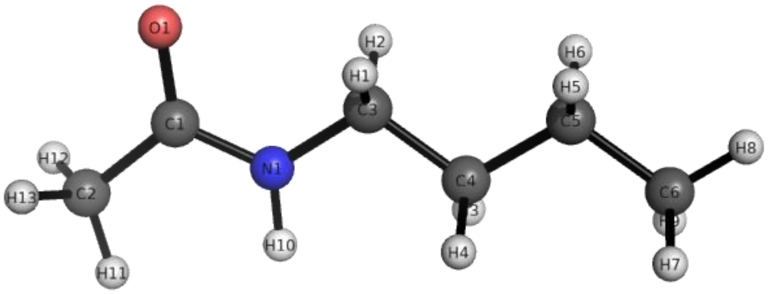


```
@<TRIPOS>MOLECULE
LYC
  21   20    1    0    0
SMALL
resp


@<TRIPOS>ATOM
      1 C1          2.2290     0.0980    -0.0000 C         1 LYC       0.811218
      2 O1          2.4440     1.3200     0.0000 O         1 LYC      -0.641673
      3 N1          0.9840    -0.4010    -0.0000 N         1 LYC      -0.509681
      4 C2          3.3530    -0.9010     0.0000 CT        1 LYC      -0.601831
      5 C3         -0.1910     0.4500    -0.0000 CT        1 LYC      -0.136733
      6 C4         -1.4590    -0.3840    -0.0000 CT        1 LYC       0.002717
      7 H1         -0.1660     1.1040    -0.8810 H1        1 LYC       0.104686
      8 H2         -0.1660     1.1040     0.8810 H1        1 LYC       0.104686
      9 C5         -2.7140     0.4790     0.0000 CT        1 LYC       0.121031
     10 H3         -1.4630    -1.0390     0.8820 HC        1 LYC       0.025164
     11 H4         -1.4630    -1.0390    -0.8820 HC        1 LYC       0.025164
     12 C6         -3.9900    -0.3510     0.0000 CT        1 LYC      -0.239027
     13 H5         -2.7000     1.1380    -0.8790 HC        1 LYC      -0.004123
     14 H6         -2.7000     1.1380     0.8790 HC        1 LYC      -0.004123
     15 H7         -4.0400    -0.9960    -0.8850 HC        1 LYC       0.055081
     16 H8         -4.8810     0.2860     0.0000 HC        1 LYC       0.055081
     17 H9         -4.0400    -0.9960     0.8850 HC        1 LYC       0.055081
     18 H10         0.8530    -1.4030    -0.0000 H         1 LYC       0.288578
     19 H11         3.0070    -1.9380     0.0000 HC        1 LYC       0.162901
     20 H12         3.9780    -0.7320     0.8830 HC        1 LYC       0.162901
     21 H13         3.9780    -0.7320    -0.8830 HC        1 LYC       0.162901
@<TRIPOS>BOND
     1    1    2 2
     2    1    3 1
     3    1    4 1
     4    3    5 1
     5    3   18 1
     6    4   19 1
     7    4   20 1
     8    4   21 1
     9    5    6 1
    10    5    7 1
    11    5    8 1
    12    6    9 1
    13    6   10 1
    14    6   11 1
    15    9   12 1
    16    9   13 1
    17    9   14 1
    18   12   15 1
    19   12   16 1
    20   12   17 1
@<TRIPOS>SUBSTRUCTURE
     1 LYC         1 TEMP              0 ****  ****    0 ROOT
```

Supporting Figure S33. The added N^{ε}-branched lysine residue (LYC) in GROMACS topology[4]. Standard charges from a neutral lysine residue (LYN) in the AMBER99FFSB-ILDN forcefield[9] were retained for atoms in the backbone and sidechain up to CG/HG1/HG2. Remaining atom charges were assigned based on RESP calculations, with residual charge correction applied on the NZ atom. No new atom types or bonded parameters were added. Two amide improper dihedrals (centered at GLY_C and LYC_NZ) involving this bond were added to the topology.

```
[ LYC ]
 [ atoms ]
    N    N         -0.41570     1
    H    H          0.27190     2
   CA    CT        -0.07206     3
   HA    H1         0.09940     4
   CB    CT        -0.04845     5
  HB1    HC         0.03400     6
  HB2    HC         0.03400     7
   CG    CT         0.06612     8
  HG1    HC         0.01041     9
  HG2    HC         0.01041    10
   CD    CT         0.00272    11
  HD1    HC         0.02516    12
  HD2    HC         0.02516    13
   CE    CT        -0.13673    14
  HE1    H1         0.10469    15
  HE2    H1         0.10469    16
   NZ    N         -0.43370    17
  HZ1    H          0.28858    18
    C    C          0.59730    19
    O    O         -0.56790    20
 [ bonds ]
    N    H
    N    CA
   CA    HA
   CA    CB
   CA    C
   CB    HB1
   CB    HB2
   CB    CG
   CG    HG1
   CG    HG2
   CG    CD
   CD    HD1
   CD    HD2
   CD    CE
   CE    HE1
   CE    HE2
   CE    NZ
   NZ    HZ1
    C    O
   -C    N
 [ impropers ]
   -C    CA    N    H
   CA    +N    C    O
```

2. Supporting tables

Supporting Table S1. Hydrogen bonding interactions between PL^{pro} and *N*$^{\varepsilon}$-lysine-branched oligopeptides in the modelled PL^{pro}:oligopeptide complexes that were consistently observed (occurrence ≥25% out of 600 frames analyzed every ns) over the combined 3 × 200 ns MD for each peptide, other than those already identified to be conserved in the core P5-P1′ region (Supporting Figure S18). Hydrogen bonding interactions involving equivalent carboxylate oxygens (in aspartate and glutamate sidechains) are combined, while salt bridges involving carboxylate oxygens and arginine sidechain guanidino groups are combined and halved to avoid double counting. Peptide residues are italicized, while PL^{pro} residues are not. Hydrogen bonding interactions in the core P5-P1′ region that are not shown in Supporting Figure S18 are in black. Outside this region, N-terminal hydrogen bonding interactions are in blue and C-terminal hydrogen bonding interactions are in red.

	Donor	Acceptor	%		Donor	Acceptor	%		Donor	Acceptor	%
ISG15	*LYC5N*$^{\zeta}$	ASN109O$^{\delta}$1	67	IRF3-Ub	*LEU4N*	GLN174O$^{\varepsilon}$1	48	NEDD8	GLU203N	*GLY1O*	34
	VAL4N	GLN174O$^{\varepsilon}$1	55		*ARG9N*$^{\eta}$	ASP164O$^{\delta}$	41		*ASN2N*$^{\delta}$*2*	GLY160O	31
	ARG11N$^{\eta}$	TYR268O	40		*ARG9N*$^{\varepsilon}$	ASP164O$^{\delta}$	32		ASN109N$^{\delta}$2	*PRO3O*	29
	GLN174N$^{\varepsilon}$2	*VAL4O*	39		*THR3N*	GLN174O$^{\varepsilon}$1	25				
					THR75O$^{\gamma}$1	*GLU1O*$^{\varepsilon}$	25				
URM1^{N}	*PHE6N*	TYR171O$^{\eta}$	72	URM1^{+}	*PHE6N*	TYR171O$^{\eta}$	72	SUMO1	*GLN8N*$^{\varepsilon}$*2*	LEU162O	66
	ARG166N$^{\eta}$	*THR9O*$^{\gamma}$*1*	61		*SER8O*$^{\gamma}$	GLU167O$^{\varepsilon}$	71		ARG166N$^{\eta}$	*GLU9O*$^{\varepsilon}$	64
	SER8O$^{\gamma}$	GLU167O$^{\varepsilon}$	60		ARG166N$^{\eta}$	*THR9O*$^{\gamma}$*1*	54		*ILE4N*	GLN174O$^{\varepsilon}$1	47
	ASN109N$^{\delta}$2	*PRO3O*	40		GLN174N$^{\varepsilon}$2	*VAL4O*	51		LYS157N$^{\zeta}$	*GLN8O*$^{\varepsilon}$*1*	32
	GLN174N$^{\varepsilon}$2	*VAL4O*	38		GLY271N	*GLY13O*	43		ASN109N$^{\delta}$2	*PRO3O*	29
	LYC5N	ASN109O$^{\delta}$1	37		ASN109N$^{\delta}$2	*PRO3O*	28		*VAL3N*	GLN174O$^{\varepsilon}$1	25
	THR9O$^{\gamma}$*1*	ASP164O$^{\delta}$	30		*LYC5N*$^{\zeta}$	ASN109O$^{\delta}$1	27		*GLN10N*$^{\varepsilon}$*2*	ASP164O$^{\delta}$	25
	GLY271N	*GLY13O*	27								
	THR75N	*ASP2O*$^{\delta}$	26								
TMEM59(K287)	*LEU4N*	GLN174O$^{\varepsilon}$1	34	TMEM59(K302)	ARG166N$^{\eta}$	*VAL7O*	47	CoV2N	ARG166N$^{\eta}$	*VAL7O*	54
	GLN174N$^{\varepsilon}$2	*LEU4O*	30		*ARG11N*$^{\eta}$	TYR268O	38				
					LEU4N	GLN174O$^{\varepsilon}$1	30				
					LEU6N	SER170O$^{\gamma}$	28				
					LEU162N	*ARG3O*	26				

Supporting Table S2. Assignment of ionic and protonation states of titratable residues of PL^{pro} (PDB ID: 6WX4[3]) analyzed in this study. The setup is identical to that previously reported.[2] Neutral histidine residues that are N^{δ}-protonated and N^{ε}-protonated are referred to as "HID" and "HIE", respectively, according to the AMBER force field nomenclature.[9] Doubly protonated, positively charged histidine residues are referred to as "HIP". The N- and C-termini of PL^{pro} were capped as *N*-acetyl and *N*-methyl amides, respectively. In the peptides, the N-termini were uncapped, while the C-terminus of the PL^{pro} S′ site binding fragment was capped with an NH_2 group. Peptide residues in the S′ binding fragment are labelled with a prime symbol (′), and for ease of setup and subsequent analysis, are numbered according to their positions relative to the N^{ε}-branched lysine, with the lysine always being residue 5′ regardless of the length of the S′ binding fragment.

Residue	LYS (+1)	ARG (+1)	ASP (-1)	GLU (-1)	CYM(-1)	HID (0)	HIE (0)	HIP (+1)	NT (+1)	ZN(+2)	**TOTAL**
PL^{pro}	6	3	12	1	111	89	47	17		1	
	43	65	22	51	189	175	50	272			
	45	82	37	67	192	275	73				
	53	138	40	70	224		255				
	91	140	61	124	226						
	92	166	62	143							
	94	183	76	161							
	105		108	167							
	126		134	203							
	157		164	214							
	182		179	238							
	190		286	252							
	200		302	263							
	217			280							
	218			295							
	228			307							
	232										
	254										
	274										
	279										
	292										
	297										
	306										
	315										
**Charge**	**+24**	**+7**	**-13**	**-16**	**-5**	**0**	**0**	**+2**	**0**	**+2**	**+1**
Peptides											
ISG15		9, 11, 6′		1′					1, 1′		
**Charge**		**+3**		**-1**					**+2**		**+4**
IRF3$_{189-197}$-Ub		9, 11, 6′		1, 1′			5		1, 1′		
**Charge**		**+3**		**-2**					**+2**		**+3**
NEDD8		11, 6′		1′			5		1, 1′		
**Charge**		**+2**		**-1**					**+2**		**+3**
URM1^{N}		6′	2	1′			11		1, 1′		
**Charge**		**+1**	**-1**	**-1**					**+2**		**+1**
URM1^{+}		6′	2	1′				11	1, 1′		
**Charge**		**+1**	**-1**	**-1**				**+1**	**+2**		**+2**
SUMO1		6′	2	1, 5, 9, 1′					1, 1′		
**Charge**		**+1**	**-1**	**-4**					**+2**		**-2**
TMEM59$_{285-291}$		9, 11, 8′		1, 3′			5		1, 1′		
**Charge**		**+3**		**-2**					**+2**		**+3**
TMEM59$_{298-305}$		9, 11, 3′	8′	1, 7′			5		1, 1′		
**Charge**		**+3**	**-1**	**-2**					**+2**		**+2**
CoV2N$_{335-341}$		9, 11	7′, 8′	1			5		1, 1′		
**Charge**		**+2**	**-2**	**-1**					**+2**		**+1**

3. References

1 Zhang Y, Sanner MF. AutoDock CrankPep: combining folding and docking to predict protein–peptide complexes. *Bioinformatics*. 2019;35:5121-5127.

2 Chan HTH, Brewitz L, Lukacik P, Strain-Damerell C, Walsh MA, Schofield CJ, Duarte F. Studies on the selectivity of the SARS-CoV-2 papain-like protease reveal the importance of the P2' proline of the viral polyprotein. *bioRxiv*. 2023;https://doi.org/10.1101/2023.07.11.548309.

3 Rut W, Lv Z, Zmudzinski M, Patchett S, Nayak D, Snipas SJ, El Oualid F, Huang TT, Bekes M, Drag M, Olsen SK. Activity profiling and crystal structures of inhibitor-bound SARS-CoV-2 papain-like protease: a framework for anti–COVID-19 drug design. *Sci Adv.* 2020;6:eabd4596.

4 Abraham MJ, Murtola T, Schulz R, Páll S, Smith JC, Hess B, Lindahl E. GROMACS: high performance molecular simulations through multi-level parallelism from laptops to supercomputers. *SoftwareX*. 2015;1-2:19-25.

5 Daura X, Gademann K, Jaun B, Seebach D, van Gunsteren WF, Mark AE. Peptide folding: when simulation meets experiment. *Angew Chem Int Ed.* 1999;38:236-240.

6 Cornell WD, Cieplak P, Bayly CI, Gould IR, Merz KM, Ferguson DM, Spellmeyer DC, Fox T, Caldwell JW, Kollman PA. A second generation force field for the simulation of proteins, nucleic acids, and organic molecules. *J Am Chem Soc.* 1995;117:5179-5197.

7 Bayly CI, Cieplak P, Cornell WD, Kollman PA. A well-behaved electrostatic potential based method using charge restraints for deriving atomic charges: the RESP model. *J Phys Chem.* 1993;97:10269-10280.

8 Cornell WD, Cieplak P, Bayly CI, Kollman PA. Application of RESP charges to calculate conformational energies, hydrogen bond energies, and free energies of solvation. *J Am Chem Soc.* 1993;115:9620-9631.

9 Lindorff-Larsen K, Piana S, Palmo K, Maragakis P, Klepeis JL, Dror RO, Shaw DE. Improved side-chain torsion potentials for the Amber ff99SB protein force field. *Proteins*. 2010;78:1950-1958.
